# Supplementary material for: Factors which Influence Risk Taking and the Evolution of Social-Identity in Stroke Narratives: A Thematic Synthesis
Source: Behav Sci (Basel). 2020 Jan 31;10(2):46. doi: 10.3390/bs10020046 (PMC7071490; doi:10.3390/bs10020046)

Supplementary File containing

1. **Example of a search**
2. **Demographics table**
3. **Processes of synthesis**
4. **Synthesis output**

Table 1 initial detailed MEDLINE search for stroke narratives synthesis

#	Search History	Results
1	(Stroke or cerebrovascular accident or CVA).mp. [mp=title, abstract, original title, name of substance word, subject heading word, floating sub-heading word, keyword heading word, organism supplementary concept word, protocol supplementary concept word, rare disease supplementary concept word, unique identifier, synonyms]	723010
2	(Narrative or Narratives or Storytelling or story telling or stories).mp. [mp=title, abstract, original title, name of substance word, subject heading word, floating sub-heading word, keyword heading word, organism supplementary concept word, protocol supplementary concept word, rare disease supplementary concept word, unique identifier, synonyms]	275626
3	Factors.mp. [mp=title, abstract, original title, name of substance word, subject heading word, floating sub-heading word, keyword heading word, organism supplementary concept word, protocol supplementary concept word, rare disease supplementary concept word, unique identifier, synonyms]	7118836
4	1 and 2	9011
5	3 and 4	5288
6	limit 5 to English language	5284
7	limit 6 to full text	1127
8	limit 7 to human	1118
9	limit 8 to peer reviewed journal [Limit not valid in Ovid MEDLINE(R); records were retained]	1118
10	limit 9 to year="2009 -Current"	657
11	limit 10 to last 10 years	657
12	limit 11 to "all adult (19 plus years)"	637
13	remove duplicates from 12	503

Demographics table

Study	Qualitative Methodology and Method	Participants	Sampling and Setting	Aims of study	Key themes
Bourland, Neville and Pickens, (2011)	*Methodology:*<br>Phenomenological design<br>*Method:*<br>Qualitative In-depth interviews	*Participants:*<br>6 participants<br>(3 male, 3 female)<br>*Age:* 58-79<br>Married (4)<br>Divorced (1)<br>Widowed (1)<br>*Post stroke:*<br>Between 21 months – 11 years	*Sample method:*<br>Purposeful sampling. recruited through word of mouth from local stroke support groups and occupational therapists(OT) practising in the community.<br>*Setting:* participants homes (1 interviewed in place of work)<br>*Country:* USA	To understand the lived experience of quality of life in 6 long term stroke survivors.	*Themes:*<br>1 main theme and 2 sub themes identified<br><br>-Main theme:<br>"it makes me feel like a human again<br>-Sub theme 1:<br>Activity loss "that's what I miss"<br>-Sub theme 2:<br>Awakening "Reality come to your world<br>*Recommendations:*<br>Further research to focus on identifying intervention programs that best facilitate occupational participation in valued activities.

Brown et al., (2010)	*<u>Methodology:</u>*<br>Interpretive Phenomenological design<br>*<u>Method:</u>*<br>Semi-structured in-depth interviews<br><br>Also asked to bring photographs of living successfully with aphasia means.	*<u>Participants:</u>*<br>25 participants<br>(13 male, 12 female)<br>*<u>Age:</u>* 38-86 (mean 63.2 -+ 12.2)<br>Living alone - 5<br>Living with 1 or more people - 20<br>*<u>Post stroke:</u>*<br>At least 2 years	*<u>Sample method:</u>*<br>Purposeful sampling. recruited through community-based stroke groups, the University of Queensland aphasia clinics and The Aphasia Registry.<br>*<u>Setting:</u>* The University of Queensland<br>And 1 in a public library.<br>*<u>Country:</u>* Australia	The aim of this study was to explore, from the perspective of people with aphasia, the meaning of living successfully with aphasia.	*<u>Themes</u>*<br>4 main themes<br><br>**Doing things.**<br>**-**Independence<br>-Ability and achievements<br>-purpose or usefulness<br>-pleasure and well-being<br>-stimulation for the brain<br>**Meaningful relationships.**<br>-support<br>-acceptance and understanding<br>-social companionship<br>-caring for others/ maintaining relationship roles<br>**Striving for a positive way of life.**<br>-positive attitude and perseverance<br>-acceptance

					-moving ahead, getting on with and enjoying life<br>-focusing on improvements<br>-appreciation<br>-trying new things and learning to adapt<br>-sense of humour<br>-it takes time<br>**Communication.**<br>-emphasis on verbal communication skills<br>-the integral role of communication across themes<br>-<br>*Recommendation*<br>Further research is indicated to develop the concept of living successfully with aphasia further and explore how best to work in partnership with individuals with aphasia to ensure their priorities of living with aphasia are addressed.

Cross & Schneider, (2010)	*<u>Methodology:</u>*<br>Did not specify - most likely Interpretive design<br>*<u>Method:</u>*<br>Semi-structured interviews	*<u>Participants:</u>*<br>10 participants<br>(0 male, 10 female)<br>*<u>Age:</u>* 71-100 (Average 81)<br>Widowed -4<br>Married -4<br>Religious sister - 2<br>*<u>Post stroke:</u>*<br>At least 6 months<br>(1 year-13 years(mean – 5years))	*<u>Sample method:</u>*<br>Purposeful sampling. From a medical clinic from a local convent within the community of southwestern Ontario<br>*<u>Setting:</u>* Local outpatients setting<br>*<u>Country:</u>* Canada	To explore the perceived influence of hope on stroke recovery in women who were at lest 6 months post stroke. What factors influenced stroke in the later stages.	*<u>Themes</u>*<br>1 main theme and 3 subthemes.<br>**Factors which influence stroke**<br>-internal factors<br>-external factors<br>-personal factors<br>*<u>Recommendations</u>*<br>Examine the influence of hop on individuals whoa re left with significant long-term impairments such as paralysis affecting mobility or dysarthria affecting communication.<br>Examine specific factors influence hope in individuals who are left with more severe impairments. Role of stroke support groups.
	<u>Methodology:</u><br>Qualitative descriptive analysis	*<u>Participants:</u>*<br>10 participants (blogs)	*<u>Sample method:</u>*<br>The data was gathered from the	Article addresses the following question: what the impact of	*<u>Themes</u>*<br>3 main themes, 7 sub themes

	<u>Method:</u><br>Internet based blog search. Self-reported	(4 male, 6 female, 1 unknown)<br>*<u>Age:</u>* 26-69 (mean)<br>All partners were Married or had a partner.<br>*<u>Post stroke:</u>*<br>>6 months	internet and analysed without the researcher directly with the participants<br>*<u>Setting:</u>* N/a<br>*<u>Country:</u>*<br>Blogs from:<br>America (7), Britain (2), Turkey (1)	aphasia on a person's social relationships as is evidenced in their blog posts.	**Impact of stroke and aphasia on social relationships**<br>-Family<br>-Friendship<br>-wider social network<br>**Support received**<br>-practical support<br>-emotional support<br>**Other people responses to the stroke and aphasia**<br>-responses of family member<br>-responses of friends and the wider community.<br>*<u>Recommendations</u>*<br>The use of social media by people with aphasia should be further explored , both as a therapeutic outlet and also as a way for people with aphasia to feel connected to a wider community.
France et al., (2013)	*<u>Methodology:</u>*	*<u>Participants:</u>*<br>18 participants	*<u>Sample method:</u>*	To provide an insight into men's and	*<u>Themes</u>*

	Interpretive Phenomenological design<br>*Method:*<br>Narrative interview lasting 1-5 hours. Narrative approach to limit interruptions. Audio and video recordings.	(9 male, 9 female)<br>*Age:* 44-85 (mean 67)<br>Married - 9<br>Widowed- 4<br>Divorced - 3<br>Single - 2<br>*Post stroke:*<br>6 months- 7 years	Diverse purposive sampling. recruited via support groups, charities, newsletters and web sites, general practitioners, physiotherapists, occupational and speech therapist, nurses, press advertisement and word of mouth.<br>*Setting:* patients home of their choice of location<br>*Country:* United Kingdom	women's experiences of life after having a stroke through examining the particular genres they adopted in their narratives. They set to answer 3 questions: which if any of Franks (1995) narrative genres fit these stroke narratives? Are there similarities or differences in the narratives forms presented by men and women? How do other factors shape the kinds of narratives presented?	Narrative genre:<br>Quest memoir<br>Restitution<br>Chaos<br>Despair<br>*Recommendations*<br>This and other studies have highlighted the similarities between men and women's experiences of certain serious illnesses. This highlights the value of comparing women's and men's experiences and challenges taken-for-granted tacit assumptions that they will react differently to ill health. From this analysis this seems less influential on a persons experience than a idiosyncratic combination of factors, including the

					severity and nature of the illness and he degree of anticipated or actual recovery
Glintborg & Krogh., (2015)	*Methodology:*<br>Qualitative research method - Part of a larger Mixed-method longitudinal study<br>*Method:*<br>Two semi-structured interviews once in hospital and once 1 year post stroke	*Participants:*<br>43 participants included but only 1 participate case study was reported<br>Female - 1<br>*Age:* 27<br>*Post stroke:*<br>1 year	*Sample method:*<br>From the Bronderslev Neurorehabilitation centre hospital admission between 2013-2014.<br>*Setting:* Bronderslev Neurorehabilitation centre<br>*Country:* Denmark	The aim of this study was to explore the identity formation process following an acquired brain injury (ABI).	*Themes*<br>-Physical construction of ABI<br>-Self positioning an implications for identity.<br>-Ambivalence of identity reconstruction.<br>-The challenges in negotiating identity dilemmas<br>-Negotiation and development of identity dilemmas<br>*Recommendations*<br>We have to reach a more widespread understanding of self identity, and more importantly, learn how to use and bring this knowledge into a more holistic understanding of ABI rehabilitation.

Green & King, (2009)	*Methodology:*<br>A Descriptive qualitative study<br>*Method:*<br>Semi-structured individual telephone interviews. 2 open ended questions.	*Participants:*<br>52 participants<br>26 males<br>26 wife-care givers<br>*Age:*<br>Males 39-83<br>Wives 33-75<br>All Married average years 29.7<br>*Post stroke:*<br>1 year post discharge	*Sample method:*<br>Patients were recruited from a larger descriptive study.<br>*Setting:* home telephone<br>*Country:* Canada	Th purpose of this study was to explore perceptions of factors that impacted patients quality of life ad wife care-givers strain over 12 months following minor stroke	*Themes*<br>2 Main themes<br>**Being Vulnerable**<br>-Changes to patients masculine image<br>-Wife care-givers assumption of a hyper-vigilance role<br>**Realization**<br>-shared loss<br>-Changing self and relationships<br>*Recommendations*<br>Exploration of the post-discharge experience of patients and their partners post-stroke is necessary to illuminate different problems and coping strategies.
Hersh, (2009)	*Methodology:*<br>Grounded theory<br>*Method:*<br>In-depth interviews (using verbal and non-verbal communication methods)	*Participants:*<br>21 patients (13 male, 8 female)<br>16 family members<br><br>*Age:* 44-85<br>Living alone – 6	*Sample method:*<br>Purposeful sampling. recruited through previous speech pathologists<br>*Setting:* not stated<br>*Country:* Australia	This article focuses on how people with aphasia view their discharge from therapying order to shed some light on this rarely explored issue.	*Themes*<br>*3 main themes*<br>-Their biographics<br>-Their notions of recovery<br>-Their feeling about their aphasia therapy<br>*Recommendations*

		All others live with spouses<br>*__Post stroke:__*<br>At least 6 months			That the finding reflect the disempowered position of our clients with aphasia and that a more open, shared process of decision making would not only be more satisfactory for all parties but also demonstrate better outcomes of therapy itself.
Kitzmüller, Asplund and Häggström, (2012)	*__Methodology:__*<br>A phenomenological and hermeneutical approach<br>*__Method:__*<br>Semi-structured interviews conducted either separately from spouses or together dependent on the patients preference. Audio recorded and transcribed verbatim by the first author. Observations and	*__Participants:__*<br>40 participants<br>23 stroke survivors<br>(males 13 female 9)<br>16 spouses<br>(male 4 female 12)<br>2 stroke survivors children<br>(male 0 female 2)<br>*__Age:__* 30-70<br>*__Post stroke:__*<br>3-25 years	*__Sample method:__*<br>Recruited by the local leaders of the Norwegian stroke and aphasia organizations and by local leaders of home care nursing<br>*__Setting:__* conducted in patients home bar one.<br>*__Country:__* Norway	This purpose of this study was to eliminate the long-term experience of family life after stroke of stroke survivors an their spouses and children	*__Themes__*<br>The family as a lifebuoy<br>Absent presence<br>Broken foundations<br>Finding a new marital path<br>*__Recommendations__*<br>Highlights the need for professional family support as families were unprepared for the life changes that occurred. Nurses and other healthcare

	disabilities m the home setting, facial expression emotions, gesture and actions were noted and taken into data analysis.				workers should examine family relationships and communication patterns and view the family as a unit composed of unique persons with different needs. More researched needed for stroke survivors children experience.
Morris et al., (2014)	*Methodology:*<br>Qualitative exploration<br>*Method:*<br>Semi-structured in-depth interviews. Focus groups including seven and eight stroke rehabilitation physiotherapists and carers.	*Participants:*<br>65 participants<br>38 Stroke survivors (19 males, 19 females)<br>15 Stroke rehabilitation physiotherapists (15 females)<br>12 carers<br>*Age:* 20-80+<br>*Post stroke:*<br>At least 6 months	*Sample method:*<br>Purposive sampling. Screened using Mini-mental state exam, Barthel index, Physical activity scale for individuals with disabilities and Carstairs index of deprivation for inclusion criteria. Recruited through community stroke liaison nurses working in two Scottish health board areas.	Aimed to explore and compare the views of stroke survivors, carers and physiotherapists about PA after rehabilitation to examine the contextual factors that are perceived to influence survivors PA participation. A second aim was to explore and compare how physiotherapists and carers support PA after	*Themes*<br>**Outcomes**<br>Preventative<br>Restorative<br>Participatory<br>**Control**<br>Capacity<br>Capability<br>Context<br>*Recommendations*<br>Person-centred PA interventions should be designed that develop shared perspectives among physiotherapists, carers and survivors

			Setting: interviewed at home or at local university, patients preference<br>*Country:* Scotland	rehabilitation, and to examine whether their support strategies facilitated or hinder survivors to participate in PA	of PA and related goals and that contribute to adjustment to life after stroke.
Mumby & Whitworth, (2013)	*Methodology:*<br>Interpretive design<br>*Method:*<br>Semi-structured interviews with reflection on experiences<br>28 semi-structure (5 each)	*Participants:*<br>6 participants<br>3 stroke patients<br>(2 male, 1 female)<br>1 carer<br>1 volunteer<br>1 Local government employee<br>(0 male, 3 female)<br>*Age:* 40- 76<br>All married and live with spouses<br>*Post stroke:*<br>>1 year	*Sample method:*<br>Purposeful sampling. recruited through community-based stroke groups, Stroke association.<br>*Setting:* choice of home, NHS premises, University (Tyneside university)<br>*Country:* England	This study addressed adjustment process in aphasia by exploring multiple perspective from people engaged in the communication hub for aphasia in north Tyneside (CHANT) a two-year community intervention for long term aphasia.	*Themes*<br>5 themes<br>Intervention type<br>Effectiveness<br>Barriers<br>Facilitators<br>QOL<br>*Recommendations*<br>Conclusions about adjustment to aphasia in general should be cautious as the models being proposed require corroboration from a larger sample of participants and within further settings ad intervention contexts.
Nanninga et al., (2017)	*Methodology:*<br>Qualitative<br>*Method:*	*Participants:*<br>33 participants<br>(13 male, 12 female)	*Sample method:*<br>Purposeful sampling. recruited through	To enrich the discussion on ability in stroke	*Themes*<br>-Social and material differences in clinical

	Semi-structured interviews	*Age:* 31-81<br><br>*Post stroke:*<br>Between 6 months – 6.5 years	acute stroke unit at a hospital and now being seen through the stroke rehabilitation unit.<br>*Setting:* interviewed at a location convenient for the participant. A family member was also present.<br>*Country:* Netherlands	rehabilitation by translating theoretical repertories of mobility from the context of geography to rehabilitation	private and public places<br>-Ambivalences and shifting tensions in bodily, family and community life<br>-Difference in access to resources for mobility<br>*Recommendations*<br>The current focus on adherence to mobility and exercise training at home needs to be critical reviewed as it does not capture the multiplicities embodied in real-life settings.
Norris, Allotey and Barrett, (2011)	*Methodology:*<br>Qualitative Ethnography and interpretive phenomenology<br>*Method:*<br>In-depth interview with photographic facilitated interviews	*Participants:*<br>29 participants<br>11 stroke survivors (4 males, 7 female)<br>18 carers<br>*Age:* 32-69<br>*Post stroke:*<br>"for purposive sampling a range of	*Sample method:*<br>Purposeful selection through sampling frame of people with disabilities in the two districts of Aceh Tengah and Bener Meriah.<br>*Setting:* not stated<br>*Country:* Indonesia	Aim was to explore the subjective experience of stroke in central Aceh. Specific objective s included a description of the impact of stroke on the lives of those affected, to identify	*Themes*<br>-burden of a disrupted life<br>-the rebalancing of life over time<br>*Recommendations*<br>A need to capture the breadth of influences, including the disabling capacity

	over a 9 month period.	time since stroke" Suggesting >6/12		the mediating factors in that experience and to consider the findings in light of the international literature pertaining to stroke experience	of social structures, but without losing sight of the specific context and biography of the individual involved.
Price et al., (2010)	*Methodology:* Qualitative case study design *Method:* Two face-to-face semi-structured interviews open-ended questions	*Participants:* 1 participants (1 male, 0 female) *Age:* 70's (Specifics not stated) *Post stroke:* Approx 20 years	*Sample method:* Purposeful sampling. recruited through inclusion criteria. Specific recruitment not stated *Setting:* Not stated *Country:* Not stated	The aim of this study was to generate understanding of the phenomenon of resilience following a stroke and its role in supporting continuity of identity and ways in which occupational therapists might foster resilience	*Themes* Resilience through: -Drawing upon positive social support -Accessing spirituality -Having an internal locus of control -Building on past successes -Commitment to success -Having an action-orientated approach and positive personal goals for the future. *Recommendations* Further research is needed to study facilitation of resiliency as a part of intervention to promote

					occupational engagement
Radcliffe, Lowton and Morgan, (2013)	*Methodology:*<br>Not stated – most likely Interpretive Phenomenological design<br>*Method:*<br>Joint interviews with spouses and stroke survivors. Open ended question following Rosenthal's (2004) biographical narrative method	*Participants:*<br>26<br>13 stroke survivors<br>(8 male, 5 female)<br>13 spouses<br>(5 male, 8 female)<br>*Age:* 68-85<br>*Post stroke:*<br>At least 1 year	*Sample method:*<br>Participants were recruited from south London stroke registration, an ongoing population-based register.<br>*Setting:* Took place within the participants home<br>*Country:* England	This article builds on prior studies of couples co-construction and presentation of joint biographical narratives and aims to explore how older couples used narratives to make sense of a stroke and how a stroke impacted on their relationship and identities, contributing towards an understanding of how older couples assign meaning to stroke through narratives.	*Themes*<br>The "united couple"<br><br>"positive" caring relationships<br><br>"frustration" finding difficulties<br>*Recommendations*<br>Further studies are require to examine variations in the content and style of interaction that may occur at different stages of managing the demands of chronic illness and disability and for different age, socioeconomic and cultural groups
Satink et al., (2016)	*Methodology:*<br>Interpretive Narrative theory, Qualitative study<br>*Method:*	*Participants:*<br>2 stroke survivors<br>(1 male, 1 female)<br>*Age:* 67 and 68<br>*Post stroke:*	*Sample method:*<br>Drawn from a larger longitudinal study.<br>*Setting:* Took place at the participants homes	This study explores how stroke survivors act as a role managers with their spouses in the	*Themes*<br>3 main themes: -<br>Performing daily tasks<br>-Role management<br>-Co-management

	6 separate interviews with 3 months apart	21 months post stroke	*Country:* Netherlands	context of everyday activities.	*Recommendations*<br>Encourage healthcare professionals to deliver stroke self-management interventions not only to assist stroke survivors and their spouses in their role management.
Shannon, Forster and Hawkins, (2016)	*Methodology:*<br>Qualitative exploration study<br>*Method:*<br>Semi-structured interviews	*Participants:*<br>10 participants (male 8, 2 female)<br>*Age:* 76- 95<br>Living alone - 1<br>Living with 1 or more people - 9<br>*Post stroke:*<br>8-12 months	*Sample method:*<br>Purposeful sampling using LUNS tool consisting of 22 statements relating to unmet needs. recruited through geographical location (Yorkshire) due to location of researchers.<br>*Setting* – participants homes<br>*Country* – England	This study aims to gain insight into why stroke survivors report low/no unmet needs	*Themes*<br>Themes 4<br>Sub themes 5<br>**Level of acceptance**<br>-Acceptance through changed circumstance<br>**Relative situation**<br>-Making comparisons with other people and circumstances<br>**Presentation of character**<br>-Valuing pride<br>-determination or independence<br>**Expectations and experiences of service**

					-viewing issues in the context of their service.<br>*__Recommendations__*<br>Requires further research to tease out barriers of identifying unmet need and facilitators of identifying unmet need.
Young et al., (2013)	*__Methodology:__*<br>Interpretive Phenomenological design<br>*__Method:__*<br>A single Open ended interview	*__Participants:__*<br>10 participants (6male, 4 female)<br>*__Age:__* mean age 52 years<br>*__Post stroke:__*<br>>26-52 weeks	*__Sample method:__*<br>participants were recruited through the hospital there were admitted to post onset and a leaflets with inclusion criteria for participants.<br>*__Setting:__* at the local hospital<br>*__Country:__* England	The purpose of this study is to explore the patient perspective of this symptom, how it is experience, and its subjective impact on the patient.	*__Themes__*<br>6 main themes, 15 sub themes:<br>**Tiredness/sleep**<br>-Sleep<br>-tiredness<br>-Exhaustion<br>**Restriction**<br>-Limitations<br>**Frustration**<br>-poor coping<br>-boredom<br>-lack of control<br>**Depression/ motivation**<br>-mood swings<br>-poor motivation<br>-doing nothing<br>-depression

					**Determination /coping**<br>-achievement<br>-goal setting<br>-control<br>**Support**<br>-Mood - Advice<br>*<u>Recommendations</u>*<br>A dynamic thematic framework for understanding poststroke fatigue is a prerequisite for quantifying fatigue in stroke patients and designing potential interventions. Such a frame work would facilitate the development of complex interventions aimed at reducing fatigue in this population

Appendix C The Completed modified COREQ Soundy et al (2016)

**Domain 1: Research team and reflexivity**			Bourland, Neville and Pickens, (2011)	Brown et al., (2010)	Cross & Schneider, (2010)	Fotiadou et al., (2014)	France et al., (2013)	Glintborg & Krogh., (2015)	Green & King, (2009)	Hersh, (2009)	Kitzmüller, Asplund and Häggström, (2012)	Morris et al., (2014)	Mumby & Whitworth, (2013)	Nanninga et al., (2017)	Norris, Allotey and Barrett, (2011)	Price et al., (2010)	Radcliffe, Lowton and Morgan, (2013)	Satink et al., (2016	Shannon, Forster and Hawkins, (2016)	Young et al., (2013)
Personal Characteristics		*Study 1*																			
1	Interviewer/facilitator Which author/s conducted the interview or focus group?	If they have identified give a point, if they haven't give 0, if you unclear type U.	0 – not stated	1 – first author	1 – first author	1 AC and DF	1 – Clare Dow	1 First author with second author present	0 – not stated	1 Main author	1 First author	1 researcher(T.O.),an experienced qualitative researcher.	U- Says students undertook interview but not specific	1 first author (a movement scientist and physiotherapist	1 This was completed by MN	1 two graduate students and their faculty advisor	1 Interviews were carried out by ER, a young researcher	1- main researcher (TS) generated the data through participant observations	0 – not stated	1
2	Experience and training What experience or training did the researcher have?	AS had training and post-doctoral experiences in qualitative research	U – Research team experience provided, not specific interviewer	1 Qualified speech and language therapist	0 - Not stated	0 - Not stated	U – Simply states she is not a health care professional	0 - Not stated	0 - Not stated	0 - Not stated	0 - Not stated	U – Just says he is experienced	1 Six graduate students in their final year of SLT training were recruited and trained in "total communication"	1 a movement scientist and physiotherapist	0 - Not stated	0 - Not stated	U- just a young researcher	1 – TS is an occupational therapists	0 – as above	0 - Not stated

													techniques							
Relationship with participants																				
3	Relationship established Was a relationship established prior to study commencement?	No relationship was established.	0 – Not discussed	1 – stated rapport was built through discussion	1 – rapport was built prior to interview with outcome measures of hope	0 no direct contact with participants	0 – Not discussed	1 established	0 – Not discussed	0 – Not discussed	0 – Not discussed	1 phone call follow ups and testing prior to interview	U – stated they had the same interviewer per participant	0 – Not discussed	0 – not discussed prior to the study	1 mutual participation in a stroke camp	1 methods of contact explained	1 – This is described in detail	1	1

4	Participant knowledge of the interviewer. What did the participants know about the researcher? e.g. personal goals, reasons for doing the research	Just the focus of the work and information provided on the information sheet.	0 - - Not stated	1 – information was provided to the participants via a gatekeeper	1 This was provided on first encounter	1 Participant blogs sent an email with information regarding study	1 Given an information pack prior to interview	0 - Not stated	0 - Not stated	1 This is reported	0 - Not stated	1 informed survivors about the study by letter	U- said they got to read previous interviews but nothing specific	0 - Not stated	0 - Not stated	0 - Not stated	0 – Not stated	0 – not stated	1	1 leaflet with information provided
5	Interviewer characteristics What characteristics were reported about the interviewer/facilitator? e.g. Bias, assumptions, reasons and interests in the research topic	White (30 year old male) with bias included past experience and focus on the area of hope.	0 - Not stated	1 Author recognised being a speech and language therapists may have bis to influence interpretation	0 - Not stated	0 - Not stated	1 informed she is not a health profession so no bias	0 - Not stated	0 - Not stated	0 - Not stated	0 - Not stated	0 - Not stated	0 - Not stated	0 - Not stated	1 stated in detail and limitation this may have to data collection	0 - Not stated	0 – Not stated	0 – Not stated	0 – Not stated	0 -Not stated
**Total Score for Domain 1.**			**0/5**	**5/5**	**3/5**	**2/5**	**3/5**	**2/5**	**0/5**	**2/5**	**1/5**	**3/5**	**1/5**	**2/5**	**2/5**	**2/5**	**2/5**	**3/5**	**2/5**	**3/5**

**Domain 2: study design**																				
Theoretical framework																				
6	Methodological orientation and theory. What methodological orientation was stated to underpin the study? e.g. grounded theory, discourse analysis, ethnography, phenomenology	Subtle Realist paradigm considering common experience and development in understanding that others could relate to.	1 Phenomenological design	1 large scale qualitative methodology	1 A phenomenological theoretical approach	0 – No consideration	0 – No consideration	U unclean? Qualitative	0 – No consideration	1 Grounded theory	1 A phenomenological and hermeneutical approach	U unclean? Qualitative exploration	1 using a systematic interpretive approach	1 Qualitative research methodology, ethnographic approach	1 - ethnography and interpretative phenomenology	0 – No consideration	0 – No consideration	1 – Interpretive data analysis, narrative theory	0 – No consideration	0 – No consideration
7	Non-participation How many people refused to participate or dropped out? Reasons?	Of those contacted by AS non refused.	1 – participant unable to attend final interview	1 – exclusion was included	1 Withdrew due to health reasons	1 – no one dropped out	0 - Not stated	1 –one declined	0 - Not stated	0 - Not stated	0 - Not stated	0 - Not stated	0 - Not stated	0 - Not stated	0 - Not stated	0 - Not stated	1 seven stroke survivors declined to participate, mainly due to their ill health	0 – Does not say	1 –two declined	0
Data collection																				

8	Interview guide Were questions, prompts, guides provided by the authors? Was it pilot tested?	Interview schedule is provided.	1 – guided by scoping questions and further analyse in subsequent interviews	1 Photographs helped guide due to research into social science	1 – personalised and guided by participants responses to questionnaires	U -Not applicable	1 Franks 1995 narrative genres	1 Piloted with initial seeking interview	1 As part of a validated QOL questionnaire	0 – No testing stated	0 – No testing stated	1 Literature guided then addition question in action	1 via a guided protocol	0 – No testing stated	0 – No testing stated	1 used content analysis to apply the data to the characteristics of resilience identified by Connor and Davidson	1- Rosenthal's (2004) biographical narrative method	0 – No testing stated	1 A topic guide, generated through discussion of research aims	U- interviews were based on topics with no specific sample question.
9	Field notes - Were field notes or reflective diary made during and/or after the interview or focus group?	No.	1 journal to write in-between interviews	1 video recording to add context	1 – Yes stated	U -Not applicable	0 – not reported	1 – Yes stated	1 – Yes stated	1 – Yes stated	1 – Yes stated	0 – not reported	0 – not reported	0 – not reported	1 . A reflective diary was maintained by the researcher	0 – not reported	1 brief notes taken during the main narration.	1 – Yes stated	1 This data was used to inform subsequent interviews and to provide contextual information during analysis	1 -They made notes, including comments on nonverbal information
10	Data saturation Was data saturation discussed?	Yes in relationship to combining findings with past literature	1 research was reviewer	1 research was reviewer	0 -no mention of sample size related to literature	0 -no mention of sample size related to literature	0 -no mention of sample size related to literature	0 -no mention of sample size related to literature	0 -no mention of sample size related to literature	0 -no mention of sample size related to literature	0 -no mention of sample size related to literature	1 research was reviewer	1 as suggested by Green & Britten (1998),	0 -no mention of sample size related to literature	0 -no mention of sample size related to literature	0 -no mention of sample size related to literature	0 -no mention of sample size related to literature	0 -no mention of sample size related to literature	0 -no mention of sample size related to literature	0 -no mention of sample size related to literature
**Total Score for Domain 2.**			**5/5**	**5/5**	**4/5**	**1/5**	**1/5**	**3/5**	**2/5**	**2/5**	**2/5**	**2/5**	**3/5**	**1/5**	**2/5**	**1/5**	**3/5**	**2/5**	**3/5**	**1/5**

**Domain 3: analysis and findings**																				
Data analysis																				
11	Description of the coding tree. Did authors provide a description of the coding tree?	Complete audit trail is available in the supplementary file	1 – Van Manen for phenomenology data analysis	1 explained coding process in detail	0 -no detail given	1 frame work analysis and matrix-based analytic method	1 explained coding process	0 -no detail given	0 -no detail given	1 typical of analysis employing NUD.IST	1 – Yes discussed	1 NVivo qualitative data analysis software	1 - included	1 Coding and analysis were carried with inter-coder reliability	1 thematical analysis following the approach described by Braun and Clarke (2006	1 multi-step process over several meetings, using several methods of qualitative analysis	1 Rosenthal's (2004) biographical case reconstruction method	1 - followed the principles of the hermeneutic circle	1	0 -no detail given
12	Derivation of themes Were themes identified in advance or derived from the data?	Both. See supplementary file	1 details given	1 details given	1 details given	1 details given	U – They descried following genres rather than themes	U – Themes could be assumed but not stated outright	1 details given	1 details given	1 details given	1 details given	1 details given	U – they looked at 3 contexts related to mobility specifically	1 details given	1 details given	1 details given	U – Themes could be assumed but not stated outright	1	1 details given
Reporting																				
13	Clarity of minor themes Is there a description of diverse cases or discussion of minor themes?	Yes. Consider both results and supplementary file	1 sub themes given	1 sub themes given	0 no sub themes given	0 no sub themes given	U – Same as above	U – Themes could be assumed but not stated outright	1 sub themes given	0 no sub themes given	0 no sub themes given	1 sub themes given	1 sub themes given	0 no sub themes given	0 no sub themes given	0 – No sub themes given	U – Themes could be assumed but not stated outright	0	0 – No sub themes given	1 sub themes given
**Total Score for Domain 3.**			**3/3**	**3/3**	**1/3**	**2/3**	**1/3**	**0/3**	**2/3**	**2/3**	**2/3**	**3/3**	**3/3**	**1/3**	**2/3**	**2/3**	**2/3**	**1/3**	**2/3**	**2/3**

Figure 2. ***Modified 13-item COREQ checklist (Soundy et al., 2016).***

Author	**Domain 1: Research team and reflexivity**	**Domain 2: Study design**	**Doman 3: Analysis and findings**	**Total**
1. Bourland, Neville and Pickens, (2011)	0/5	5/5	3/3	8/13
2. Brown et al., (2010)	5/5	5/5	3/3	13/13
3. Cross & Schneider, (2010)	3/5	4/5	1/3	8/13
4. Fotiadou et al., (2014)	2/5	1/5	2/3	5/13
5. France et al., (2013)	3/5	1/5	1/3	5/13
6. Glintborg & Krogh., (2015)	2/5	3/5	0/3	5/13
7. Green & King, (2009)	0/5	2/5	2/3	4/13
8. Hersh, (2009)	2/5	2/5	2/3	6/13
9. Kitzmüller, Asplund and Häggström, (2012)	1/5	2/5	2/3	5/13
10. Morris et al., (2014)	3/5	2/5	3/3	8/13
11. Mumby & Whitworth, (2013)	1/5	3/5	3/3	7/13
12. Nanninga et al., (2017)	2/5	1/5	1/3	4/13
13. Norris, Allotey and Barrett, (2011)	2/5	2/5	2/3	6/13
14. Price et al., (2010)	2/5	1/5	2/3	5/13
15. Radcliffe, Lowton and Morgan, (2013)	2/5	3/5	2/3	7/13
16. Satink et al., (2016)	3/5	2/5	1/3	6/13
17. Shannon, Forster and Hawkins, (2016)	2/5	3/5	2/3	7/13
18. Young et al., (2013)	3/5	1/5	2/3	6/13
TOTAL AVERAGE	2.1/5	2.5/5	1.8/3	6.4/13

Synthesis Table
Stage a Opening Coding

Author	Results	Comments
1. Bourland, Neville and Pickens, (2011)	"Quality of life is doing what you want to"<br>As participants defined quality of life, they consistently indicated that the ability to participate in meaningful activities was what gave their life quality. Often, this meant participating in the same activities that they had participated in prior to stroke. Nina commented, "You didn't think about it before you had the stroke, but, now, after the stroke, it means a lot to be able to do the things you could do before." This statement not only indicates the importance of returning to previously valued activities but also reflects a change in awareness that the things she used to do added quality to Nina's life. That awareness resulted in a new appreciation for activities she had participated in before her stroke. Staying busy and active, getting out of the house, and simply having the option to come and go as they pleased were other important aspects of quality of life. For these participants, the freedom to move around their community independently gave them control over their day-to-day lives. When asked about what made her want to get up and live life each day, Ann replied, "That I can if I want to. That I can go to the store if I want to, that I can go to church if I want to, that I can do the things I want to." Participants conveyed that autonomous choice in activities was an important part of quality of life. Ann's statement reflects 2 important issues for stroke survivors when defining quality of life: first, "I can," and second, "if I want." These 2 phrases constitute the essence of how stroke survivors defined quality of life – the ability to participate in the activities they choose.<br><br>Experiencing quality of life<br>As interviews progressed, it became clear that quality of life was not experienced in a static fashion; rather, participants' experience of quality of life was dynamic, shifting along with changes in level of participation and changes in their perspectives. The dynamic characteristic of quality of life is reflected in the finding's main theme – "It makes me feel like a human	Participating in activities was important to the individual and their quality of life.<br><br>Independence gave the stroke survivors a sense of freedom and improved QOL

	again." The experience of quality of life is explored in greater depth by 2 subthemes – activity loss: "That's what I miss," and awakening: "Reality comes to your world."<br><br>"It makes me feel like a human again"<br>After stroke, survivors progressed through states of physical and psychological recovery that influenced their quality of life. As these survivors described the stroke experience, the ability to participate in "the things [the survivor] did before the stroke" was interrupted. As survivors reengaged in meaningful activities, their experience of quality of life changed over time. Ann explained this experience, describing changes in her perception of self as she regained her ability to participate, "I guess it just makes me feel like a human again, you know? Just being able to walk and talk and stand up and everything makes it feel real. Before I felt like a ghost or something." To Ann, there is a sense that in early stages of recovery she was less than human. Her state of being was not "real" from her own perspective. Her experience of recovery as she regained physical skills enabled her sense of "human-ness" to remerge. As activities were resumed or replaced, survivors also regained a sense of their self-confidence, competence, personal influence, and meaning. The dynamic nature of this process is aptly summarized in a quote from Nina, who commented: I used to do a lot of stuff, like I made cards and I made dolls, porcelain dolls and stuff. Well, I can't do those things, so the quality of life now is less than it was, and I have to work to get it back …. I can't do the porcelain dolls right now because I can't make the stroke and stuff. But, I will. Sooner or later I will. Nina was aware of her limitations but was equally hopeful that her limitations would not last. As she was able to resume doll making, the presumption was that her quality of life would be affected. In her mind, she was not stuck at one place in time but rather was working along an ever-changing continuum toward a better quality of life.<br><br>Activity loss: "That's what I miss"<br>This subtheme explores the loss of meaningful activity and personal identity as it relates to the dynamic experience of quality of life. All participants experienced the loss of some valued activity. Jim shared his experience:	Ability to participate in meaning full activities influenced change over time<br><br>At the beginning physical recovery of skills influence feeling human again<br><br>Being aware that recovery is progressive and that things they cannot achieve now they may achieve – having Hope<br><br>Identifying loss of identity and because of

	Whereas before, you know when getting up, you know and you get ready, take a shower and you get gone. And that's what I miss. Um, that was good. That since it's now, and just here, and that's it. There's nothing … everything is the same nothing. And I go in my, the uh, office and turn the TV. That's what I do. And, and that's it.<br>Sometimes relationships were lost along with specific activities. For example, when Jim retired as a result of his stroke, contact with his co-workers also stopped.<br>Um, guys I was doing that, wuster [sic], uh, company, uh I don't nothing. Uh I don't talk to them or nothing and they haven't done anything to me …. And I, I think that was a little bit of a, uh, one thing of that that I thought that would would be a bit a, uh, talked to call. I mean I would've thought that that would be OK. I mean, I mean it's not that uh I been dead. I'm not dead so, so uh, but uh, I don't know.<br>There was a sense of frustration in Jim's descriptions of days before and after the stroke; the loss of previously valued activities left a void that had not been filled with new, meaningful activities. In addition, the loss of his job led to decreased social contact, a consequence that may have further affected his quality of life. When talking about the loss of valued activities, participants also spoke about changes in the way they thought of themselves. Brister commented, "There's more to me than what you see now. I used to get out, could outrun anyone, most of the average person, I played ball and I was a typical teenager, and I was such a vibrant person." When survivors' ability to participate in valued activities was altered, they sometimes experienced incongruence between pre- and poststroke identity. Brister in particular conveyed such an incongruence: "I was such a vibrant person" before the stroke. His use of the past tense indicated changes between his pre- and poststroke identity. The loss of personal identity that accompanied the loss of meaningful activity may have also created a fear that other people viewed stroke survivors differently, as being less capable or intelligent. Brister commented:<br><br>Monday afternoon I went to see a second grandson play tennis…and as I approached every eye is on me, and here I am walking with a limp, and have to be helped to my seat, and uh, and suddenly I just have the feeling that all of them said, "look at that man who is cripple, who is lame and cannot do for himself," and then I start crying.	this resorting to doing nothing.<br><br>Lack of acceptance and adaptation to new circumstance. loss of purpose<br><br>Comparing where they were before stroke to where they are now – narrative of impossibility<br><br>Fear of others perspectives of judgement of stroke survivors being less capable or dependent – lost independence

	In retelling this story, Brister appeared humiliated by his need to be helped to his seat. Further, Brister expressed sincere grief that others might judge him as dependent. He seemed to mourn his lost independence. Activity loss was as central to a dynamic quality of life as activity gain. Whereas some participants found enhanced quality of life in new activities of meaning, others had not replaced previous activities. For those experiencing the greatest amount of loss, their statements reflected a sense of being lost, with a lack of clear direction of where to go and what to do with their lives each day. Survivors who had reintegrated meaningful activities experienced quality of life differently. Some excellent examples of this were illustrated in the words of 1 participant who found meaning as a caregiver. Mimi shared, "By giving myself to them, my selfish side is saying I'm trying to prove to myself that I'm okay, that I'm in good health, that I'm able to take care of people, that I am strong, and I, I can go on with my own life." Mimi was not just saying that caring for her family makes her happy, although she stated that it is "the greatest joy" she has. She was making the point that caregiving makes her feel healthy, capable, and accomplished. Her quality of life is enhanced. Different participants illustrate steps along the continuum of quality of life and evince the dynamic nature of the stroke survivors' experience.<br><br>Awakening: "Reality comes to your world"<br><br>For the stroke survivors who participated in this study, awakening conceptually describes a gradual realization or change that took place as a result of stroke. Although awakening was not experienced by all the survivors in the same way, changes in values, perspectives, and behaviours emerged as a common experience. Mimi shared the following:<br><br>In fact, when you have a stroke, and this is very important, when you have a stroke reality comes to your world….You know what is the most important that you treasure. I would sit out there on the patio…and I would look at the air and the trees and the and the birds and the sky, whereas before I was too busy to do all this. Then I figured, you know, what are we doing?	Narratives of being lost and not having hope for enhancing QOL.<br><br>Focusing on new meaningful activities to gain back a sense of purpose and not being a burden.<br><br>Realise the gains to happiness from opening eyes to new experiences and slowing down-acceptance of condition and capabilities,

	Why don't we slow down and look at what is real?...That, to me, brought it all to my attention, my stroke.<br><br>Mimi's stroke was a catalyst for reprioritizing her life. Post stroke, she took time to enjoy small things and became a caretaker for ill family members. Her awakening led to an increase in her quality of life after stroke, which she described as "beautiful." For others, awakening was reflected in thankfulness for family or the ability to continue participating in valued activities. Sometimes, awakening illustrated the participant's loss, as when Nina acknowledged missing abilities she had previously taken for granted ("You don't miss it till you don't have it"). At times, awakening resulted in a greater appreciation for the reality of mortality ("I thought I was bullet-proof"), changes in health behaviours, or the awareness of how one's behaviour affects others. Sam commented, "I have a better attitude towards myself and other people, and um, you know, for that I'm thankful. I guess I just pay attention to more details and the quality of things that you do and go from there." Sam, like Mimi, found he appreciated the details of life more. He also adjusted his attitude and behaviours as part of a greater effort to influence the quality of his life and relationships. Sam's was not the only case in which awakening led to changed relationships with others. Although he lost contact with co-workers after his stroke, Jim rekindled his relationship with his son.<br><br>Well, what I have seen is that uh, that, that what I have I have really seen at my boy….Uh, see and talk to him uh that I have, have been more…I haven't seen him like that for years because I haven't done it, 'cause I always working. And now I see much and I see a lot more and it's, and I think that's the one thing out of it, that I really see him.<br><br>For stroke survivors in this study, strengthened familial relationships were important anchors for quality of life. Awakening was often associated with reclaiming lost activities with a different perspective. For example, Sam exercised greater control over frustration with his family. Others established entirely new activities, as with Mimi, who found better quality of life as a caregiver for her family than she had as a paid worker. For survivors, the quality of their lives was reframed by their experience with stroke. Many of the changes they experienced as a	Changes in behaviour to see the world differently as a coping mechanism<br><br>Importance of support network and family for QOL

	part of awakening led to positive results. Regardless of the specific way in which each participant experienced awakening, it seemed to be an essential part of how the survivors coped with changes resulting from stroke. Awakening was an integral component in how participants perceived poststroke quality of life.	
2. Brown et al., (2010)	Placing participant responses in context: The negative and positive lived experience of aphasia and participants' self-ratings of success Participants referred to both negative and positive experiences to inform their concepts of living successfully with aphasia. It was clear that many participants did not distinguish between the impact of stroke and that of aphasia, and so comments reported reflect participants' perceptions of aphasia, which may in reality encompass broader stroke-related issues. For a few participants the overall lived experience of aphasia was strongly negative. A participant who rated herself as living "somewhat successfully", expressed her view that living successfully with aphasia was an impossibility for herself and others: "I can't be successful . . . Doesn't matter which . . . [person with aphasia] you're speaking to. They can never be successful . . . Impossible. Nobody can . . . It will always be there in the back of their mind." Other participants provided evidence that some people with aphasia do perceive themselves to be living successfully with it. One participant described a self-portrait taken for the photo interview: "There's me. And I am a success with aphasia." Others commented, "I can live . . . successfully with aphasia. All my family . . . everybody jumps in and I find aphasia—I can . . . very confidently live with it," and "I'm most happy with my life—yeah." The self-ratings of living successfully with aphasia indicated that the majority of participants recorded a rating somewhere in between these two extremes. A total of 13 participants rated themselves as living "somewhat successfully" with aphasia, 8 rated themselves as living "successfully," 3 rated themselves as living "very successfully", and only 1 participant gave a rating of "not successful". Thus both negative and positive experiences with aphasia informed people's definitions of living successfully with their communication disability. Overall, when talking about living successfully with aphasia, participants' comments referred both to those factors that were currently present in their lives, and to things that were desired for the future, or missed from their past. Factors that were currently present in their lives were seen as contributing to living successfully with aphasia (e.g., "The most important thing is my partner"). The second group of factors was represented by participants through statements	An inability to live successful. A narrative of impossibility. Definition of successful living is based on able bodied living. Emphasis on loss.<br><br>Part of the definition of successful living is embracing it<br>The barriers to aphasia can be overcome<br>Being part of a supported situation may help living or facilitate what being a success looks like.<br>The embracement and living 'successfully' is possible for most.<br><br>Important social relationships represent successful living

	reflecting what it would be like if they were living successfully with aphasia (e.g., “If I was . . . painting and driving”) or describing elements absent from their lives which therefore detracted from them living successfully with aphasia (e.g., “I don’t have the girls [girlfriends] . . . that I can talk about stuff [with]”). Throughout the results, the contrast between these two groups of factors is highlighted.<br>LIVING SUCCESSFULLY WITH APHASIA 1277	Needs including meaningful activities and relationships
	Thematic analysis<br>Analysis of participant transcripts revealed that the concept of living successfully with aphasia was multifaceted and highly individualised from participant to participant; however, threads of commonality across the participant group were also identified. A number of core themes relating to the meaning of living successfully with aphasia included the importance of doing things; meaningful relationships; and striving to maintain a positive way of life. A fourth core theme of communication was also identified. Within the theme of communication a strong emphasis was placed on verbal communication, but analysis also revealed that communication was interwoven through the three other core themes; playing a role in doing things, relationships, and striving for a positive way of life. Alongside the four central themes above, four other themes also emerged from the data: health and physical capabilities; external support; living in one’s own home; and lack of public awareness and a need to tell people about aphasia. Themes identified are described in further detail below.	
	Core theme 1—Doing things: “Do as much as you can.”<br>“Be involved with everything.” “Have a hobby.” “Live as much as you can; do as much as you can.” These participant statements illustrate clearly one predominant theme that emerged from the data: the central role of “doing things” as a core component of living successfully with aphasia. Activities of importance for living successfully with aphasia fell broadly into three categories: activities centred around the home and household management; hobbies or activities of leisure; and work or further education activities. Participants described continuing activities from before their stroke, adapting old activities to enable continued participation, and finding new interests and activities. It was evident that participants engaged in a wide	Meaningful activities that contribute to the importance of successful living<br><br>Enablement of living including adapting old activities and finding new interests

	variety of meaningful activities, and Table 2 provides an overview of these. Participants contrasted doing things as part of living successfully with aphasia with sitting at home doing little. Doing little was described as counteractive to living<br>1278 BROWN ET AL.<br><br>successfully with aphasia: "The worse thing they could do . . . You sit at home in a house . . . watch TV—no no. That's not right (laughs)"; "If there's most people around that say, oh I'll do nothing. They're going downhill. Actually going downhill (participant discussed significance of photos of his vocational training)." This contrast also highlighted the value placed by participants on doing things. In expressing the importance of "doing things" participants were not only able to describe those particular activities in which they engaged, but in some cases, provide further details about why doing the activity was of significance. Further analysis of participant statements coded under the theme of doing things revealed that doing things was significant for living successfully with aphasia largely because it provided participants with a sense of:<br>• Independence<br>• Ability and achievement<br>• Purpose or usefulness<br>• Pleasure and well-being<br>• Stimulation for the brain.<br>Less predominantly, doing things also served as a way to prevent boredom or keep their mind off troubles; assisted participants in meeting people; and, for one participant, highlighted the role of her work activities in her self-identity: "that's important because that's what I do . . . pertinent to me."<br>Doing things – Independence: "By myself." The sense of independence gained from being able to do things "by myself", was revealed to be an important reason why participants valued "doing things" so highly, in order to feel like they were living successfully with aphasia. Participants sought independence across all arenas of their lives. This encompassed both activities that would be impacted on greatly by their aphasia, for example, independence in ordering in a restaurant: "If you're going out for dinner . . . make sure that you are . . . you do	Critical to viewing living as successful or meaningful is engagement in activities that have a purpose<br><br>Recognising that sitting at home and doing nothing is a factor for regressing and "going down hill"<br><br>Value of doing thing was expressed to improve ability and allow for achievement<br><br>Self identity recognised by doing things<br><br>Independence gaining ability to do things by their self – participants sought after independence across all areas of life.

	it. With yourself," and activities with no relation to communication, for example, one participant described a photo of her putting on make-up: "I'm learning how . . . want to have my face done . . . very important. Because he's [my husband's] good. But he's not a woman. So I want to do myself. Very important." Other examples included independence in making decisions regarding their lives: "Be involved in making decisions . . . about the family and about them"; managing household jobs: "I can shop by myself (comment referring to photo of a shopping centre)"; and in travelling locally: "I get a bus to my stop and then I have ownership of it . . . But I do it myself."<br>There was a strong contrast between participants who perceived they had some level of independence in their lives and those who did not. For some participants who felt they lacked independence, this absence marked a significant factor preventing them from hopes of living successfully with aphasia, with one participant commenting, "I need help with anything I do," and another participant describing why no one she knew with aphasia was living successfully with it: "They're always (with emphasis) dependent." A desire for greater independence in the future in order to be living more successfully with aphasia was thus, felt strongly: "Want. To. Do. Things. Myself."<br><br>Doing things – Ability and achievement: "I can." Another feeling engendered by participants engaging in "doing things" was a sense of ability and achievement. A strong focus on "I can" statements in participant transcripts highlighted the importance of recapturing feelings of ability for living successfully with aphasia: "I can do that"; "There's heaps of things round the place I can do"; "That's being able to do things . . . so I can—yeah." Further illustrating an emphasis on ability, many participants chose to take photographs of things they were able to do, with one participant commenting, "With the camera you made me see what I can do." Participants' derived a sense of ability from a wide variety of everyday activities. For a subset of participants larger milestones were the reinstatement of their driver's licence, returning to work or volunteer work, and completing further education. A real sense of achievement was evident for participants in describing these milestones: "I got the job . . . I accomplished something that I didn't think I'd be able to do"; "That's an award (referring to photo of a certificate) I got for . . . volunteer[ing] . . . I do my best—quite [an] achievement . . . (laughs)."	Still being able to identify as a wife within social living by being able to do activity – makeup<br><br>Importance of independence<br><br>Being dependent and expressing desire to be independent but barriers to this.<br><br>"I can" positive attitude to situations being a factor for achievement and ability.<br><br>Goal setting and achieving milestones facilitated living successfully with aphasia.

	Participants also reflected that their sense of achievement for completing even simple activities was heightened because of the increased difficulty they experienced because of their aphasia and/or physical impairments: “Cooking meals. Like my kids don’t even recognise that it’s anything different. But it is—it took a lot”; “To fill out the paperwork (for workrelated application) . . . it’s hard (with emphasis)—you know—but I did it.” Again, participants who described an inability to take part in the activities they wished to, felt that this impacted negatively on their hopes of living successfully with aphasia: “Because I can’t get out there and do what I want to do. So—just got to stay here to do nothing.” For others, there was a balance between those things they could no longer do, and finding things they were still capable of: I can’t read anymore . . . spelling is horrible since my stroke . . . I can’t do whatever I used to do. And I would—I feel that I’m useless . . . [But] I’m not depressed and . . . I laugh . . . And I am finding that I am living successfully with the stroke. Yes . . . I go for a walk. I ride the bike (indicates to exercise bike in lounge) . . . go out shopping with my wife. And go for an overseas trip. And I feel alright—yes. Thus a sense of ability and achievement from doing things was seen as a positive contributor to living successfully with aphasia, but its absence was seen as a detractor; while some participants adapted by finding a balance between things they could do and those they could not.<br>Doing things – Purpose and usefulness: “You’re not useless.” Alongside a sense of independence and a sense of ability and achievement, participants also found a sense of purpose or usefulness in being able to do things. This was described aptly by one participant who commented, “‘Cause you can realise . . . you’re not useless. You can get up and do something.” Activities centred around household management or work and further education were cited most frequently as contributing to a sense of purpose or usefulness, but hobbies/leisure activities were also commented on as providing purpose: “You gotta have an interest like carving or reading . . . it gives you something to work for. It gives something to try and succeed with something . . . You got to work towards something.”<br>Doing things – Pleasure and well-being: “Makes me happy.” Participants also gained pleasure and well-being from engaging in meaningful activities. Time spent on hobbies or leisure activities offered participants time for relaxation or solitude: “Time for myself (referring to photo of meditation CDs)”, “This is releasation [relaxation] (describing photo of dart-board)”;	Not managing expectation of unrealistic goal setting causing negative feedback from activities – “so do nothing”<br><br>Finding a balance between what can and cant be achieved facilitated living successfully with aphasia. Priorities<br><br>Realising that they are not useless if they can do things or activities gaining back purpose and usefulness (hobbies/leisure activities)

	enjoyment: “It’s fun. It’s fun”; and for maintaining a positive mood: “Makes me happy (discussing photo of gardening)”, “Keep you—spirits up—yeah.”<br>Doing things – Stimulation for the brain: “Keep your brain working.” Lastly, doing things contributed to living successfully with aphasia by providing participants with stimulation for their brains. Participants suggested that keeping your mind working was essential for living successfully with aphasia: “It’s important to get out and use your brain”; “They’ve got to have stimulation in their brain.” A variety of activities were suggested for this purpose, including: sewing, knitting, puzzles, playing bridge, and work or volunteer work.<br><br>Core theme 2—Meaningful relationships: “It’s really people.” A second theme identified by participants as central to living successfully with aphasia was meaningful relationships. As one participant stated: “I think it’s really people . . . that make you feel successful.” Another described a photo of his friends and family by saying: “Living cessfully [successfully]—friends and family . . . important.” Those relationships highlighted by participants as being of value for living successfully with aphasia included relationships with family, friends, other stroke and/or aphasia survivors, and neighbours, with some participants also describing their relationships with pets as contributing towards living successfully with aphasia. Meaningful relationships contributed to living successfully with aphasia in a multitude of ways; however, those themes identified most strongly by participants included the importance of support from other people, acceptance and understanding, social companionship, and caring for others /maintaining relationship roles. These areas are expanded on further below.<br>Meaningful relationships – Support: “Don’t do it alone.” Overwhelmingly, participants highlighted that the support offered through meaningful relationships was a key component contributing to living successfully with aphasia. These participants’ statements illustrate the emphasis placed by participants on the value of support: “Don’t do it alone. Friends and family need to support them.” “Support you . . . is a big thing.” Two main types of support identified were practical support, in the form of assistance with household chores, and emotional support, mainly in the form of ongoing encouragement. Examples of practical support provided included assistance with tasks such as lawn mowing, shopping, and housework, while	meaningful activities correlates to improved mood and enjoyment<br><br>living successfully with aphasia by providing participants with stimulation for their brains<br><br>meaningful relationships aiding with feeling of success and living successfully.<br><br>Even living with pets can aid this<br><br>Meaning full relations ships to both encouragement towards achieving

	participants described emotional support or encouragement both in terms of encouragement towards achieving specific goals (for example, encouragement provided by one participant's mother to read a book: "My mother kept on me and on me and on me . . . She made sure I read a book. Although it took me a year (laughs) I read a book . . . it was just the little things that she made me do") and more general overall encouragement (for example one participant describing how her friends helped her to live successfully with aphasia, "People around you . . . pulling them [you] up . . . Come on. You can do it. You can do it. Yeah.") A key feature of the support provided that was valued most highly by participants was its constancy, with participants accentuating through their descriptions of photos of friends and family, the fact that those closest to them were "always there" when they needed them. "Mum and Dad . . . They have [been there through the] . . . ups and downs"; "[My wife] and those sorts of people . . . you know they're always there"; "Friends are all important . . . they were there for me." In terms of emotional support provided, a distinction was made by some participants between support from family members, which was welcomed, and sympathy, which was not. As one participant stated, "I didn't have any pity from my family which was the best thing. Just support. And that's what I think if someone has aphasia you need—you need support. You don't need pity. You don't need someone, oh I'm so sorry. That doesn't get you anywhere." Meaningful relationships – Acceptance and understanding: "Understanding." Alongside support, acceptance and understanding from those closest to participants were valued highly as contributing to living successfully with aphasia. The inherent acceptance and mutual understanding between fellow stroke/aphasia survivors was of particular significance to participants. In addition an emphasis was placed on family and friends treating participants no differently than before their stroke. One participant described his relationship with a group of friends in terms of their tolerance and understanding: "[Friends at my group for retired businessmen] are understanding . . . tolerance . . . that's how I found those people." Another participant, who was a mother of two school-aged children, shared her fears that her children would treat her differently, and how their acceptance was so meaningful. She described a photo of her children by saying:<br>[My daughter] treats it like everything else . . . nothing wrong with Mum . . . [My son]—I thought he would faze to the back—faze me to the background, but he doesn't . . . His	specific goals and assistance with daily chores.<br><br>Having people constantly around all the time knowing there is always support peace of mind.<br><br>Support groups or not having pity from people but rather understanding and acceptance is important to living successfully.<br><br>Acceptance from family and understanding

	mother’s day card to me, it said—I know you’ve had a stroke, but (breaks into tears) you’re still the best Mum ever . . . That meant the world to me (crying), ‘cause coming from him . . . To them I’m just Mum . . . They don’t care [that I’ve got aphasia].<br>A few participants reported instances where a lack of understanding from family and friends had caused upset for them. “Friends . . . they didn’t know about aphasia . . . the misunderstanding of what it was . . . you had a mental problem . . . it’s very upsetting.” This contrast again illustrated the importance of acceptance and understanding from those in meaningful relationships with participants. Other stroke survivors, in particular those with aphasia, were identified as a special source of acceptance and understanding because of the inherent nature of their understanding through mutual experience. A number of participants chose to discuss photos of friends with aphasia for their photo interview. As one participant stated: “When you’ve had something wrong with you . . . you need to get friends who have the same problem . . . this is one thing that we’ve all got in common.” Another commented: “It’s good to relate to other people who have the same situation—you know . . . And it’s just nice to talk with people who . . . he knows what I have . . . And things flow that way.”<br>Meaningful relationships – Social companionship: “Socialising.” Social companionship was another strongly valued benefit of meaningful relationships. Participants’ comments suggested that proactive efforts to get out of the house and to meet and socialise with others were a requirement for living successfully with aphasia. “Mix . . . with people as much as I can . . . socialising. It helps quite a lot. Unless you’re . . . prepared to do that it makes you . . . very hard for yourself.” Time spent together with family and friends was found to be of great importance, and participants described a range of social occasions they enjoyed, for example, family meals or birthdays and holidays with friends. Keeping in touch with family and friends who lived interstate or overseas was also of importance, with participants taking photos of and describing how they used phone calls, email, and web cameras to stay in contact with family and friends. While an emphasis was placed on friendship as contributing towards living successfully with aphasia, some participants reported difficulties in maintaining existing friendships following their strokes. One participant statement illustrates clearly the perception that many friendships are lost post-stroke, increasing the value of those that remain. She described the significance of a photograph taken for the photo interview which showed her	within close social relationships is important<br><br>Surrounding self with support and friendships from people who also have aphasia as they understand – social companionship<br><br>Loss of some previous relationship due to understanding increases the bond with remaining relationships

	friend: "It's really hard when you can't talk and when you've had a stroke you lose a lot of your friends (spoken with tears in eyes). And I didn't lose her (cries silently)." Participants expressed a desire to meet more friends and also reported they did not see existing friends as frequently as they would like. Again, fellow stroke/aphasia survivors were valued highly as contributing towards living successfully with aphasia through the formation of new friendships. "Now my troke—stroke survivors are my friends." In particular participants commented on the opportunity to have fun and laugh with other stroke/aphasia survivors: "We laugh. And . . . it's just fun"; "We've all had a stroke. Been there, done that. Um—we can't talk, but we laugh." For some participants, joining other community groups (such as Rotary or Probus) also offered opportunities for new friendships. Thus while participants experienced changes in friendships following their strokes, social companionship from family and both new and old friends was seen as contributing significantly to living successfully with aphasia. Time spent socialising with family and friends was valued highly.<br>Meaningful relationships – Caring for others/ maintaining relationship roles: "[s]he got to be all right." Finally, participants described how living successfully with aphasia involved caring for others and maintaining relationship roles (for example the role of parent or husband). One participant described how his wife's well-being was linked to his own ability to be living successfully with aphasia: (Indicates by pointing to his wife) . . . [s]he got to be . . . all right."<br>Other participants described the importance of still being able to contribute by doing things for their families or partners, for example shopping for the family, organising birthday parties for a son/daughter, or looking after a partner's rose garden. For one younger participant the importance of maintaining custody of her children and her role as a mother was paramount to her concept of living successfully with aphasia:<br>I've got my kids with me part-time . . . because of the stroke and stuff . . . I wasn't sure whether I was going to get them at all . . . two kids, bringing them up successfully . . . I wanted to see them grow up. And seven years later I have (crying) . . . And, they've grown up to be good kids.<br>As these examples illustrate, caring for others and maintaining relationship roles therefore contributed towards living successfully with aphasia.	maintaining relationship roles and ensuing spouses is also happy within the relationship

	Core theme 3—Striving for a positive way of life: "Be positive." A third theme central to participants' descriptions of what it meant to be living successfully with aphasia was the importance of striving for a positive way of life. This theme represented data from participants highlighting that although the lived experience of aphasia included many negative consequences, living successfully with aphasia entailed efforts to maintain positivity and move on with life. Sub-themes identified included references to the internal personal resources or strategies participants employed to strive for or maintain positivity, such as having a positive attitude; acceptance; moving ahead, getting on with and enjoying life; appreciation; trying new things and learning to adapt; and a sense of humour. Participants also stressed that "it takes time" to learn to live successfully with aphasia. The following sections draw on data from participants illustrating the endeavour to strive for a positive way of life, despite the sometimes-negative consequences of aphasia.<br>Striving for a positive way of life – Positive attitude and perseverance: "Have a positive attitude." One factor that emerged strongly for living successfully with aphasia by striving for a positive way of life was having a positive attitude, alongside ongoing perseverance. As these participants emphasised, "I think that—important thing with any sort of disability is your attitude towards it"; "Have a positive attitude"; "It's simple—it's be positive." In addition to a positive attitude, when asked about advice they would give other people for living successfully with aphasia invariably participants stressed the importance of perseverance (also described by participants as determination, bloody-mindedness and resilience): "Never give up. Never. Never"; "When you give up . . .you might as well find a coffin as far as I'm concerned"; "It's called stick-to-it-if-its-ness . . . you can't give up."<br>Striving for a positive way of life – Acceptance: "I've got to accept it." For many participants acceptance was also a component of striving for a positive way of life. One of the most challenging things for a number of participants to accept was not returning to their pre-stroke level of functioning. One participant described his acceptance of his changed communication: "I'm trying to . . . get as close as I [can to what I was]—before the stroke . . . I know that that's not possible, but . . . as good as I can get and I'm happy." Other changes that participants described as necessary to accept in order to live successfully with aphasia included specific	Having a positive attitude impacted positively on the lives of participants with aphasia.<br><br>Realising capabilities an accepting that they may not be the same as before and accept that and move on

	inabilities following their stroke (for example, driving: “I can’t drive . . . To me that’s a big negative. [But] It’s not negative thinking. It’s . . . accepting I suppose”); and changes in lifestyle (for example, a description of a photo showing participant relaxing with his wife: “I was always a hundred miles an hour and now I have to say, well—you know—I can’t go a hundred miles an hour”). In some instances, participants were reluctant to accept changes, and still hoped for or wanted to regain functioning, at the same time as acknowledging that this might not be possible and advocating for acceptance as a positive factor for living successfully with aphasia. “And I know it’ll never be the same as what I was before I had the stroke . . . And as I say I hate to accept it, but I’ve got to accept it.”<br>Striving for a positive way of life – Moving ahead, getting on with and enjoying life: “Look forward.” Following on from acceptance, participants also described a mind-set of “moving ahead,” “getting on with life (referring to a photo of a holiday),” and aspiring to “be as happy as you can and . . . enjoy life” in order to be living successfully with aphasia. Moving ahead encompassed the idea of focusing on the future rather than the past. Participants espoused “having a vision,” or “new plans ahead,” and “look[ing] forward to what I can do,” whilst cautioning, “don’t look back,” and “you mustn’t do that . . . constantly comparing yourself [to before your stroke].” One participant commented, “It’s all moving ahead. Moving ahead. You can’t really go backwards. You can go ahead.” Getting on with life was linked closely to the theme of doing things, with participants describing this idea mainly in terms of still being involved in activities and not letting aphasia prevent them from “living”, for example: “Have a hobby . . . live life. Live life. Yes. Yes.” For some participants this included maintaining a sense of normality: “I’m living my own life the way I normally want to be.” Participants also stressed the importance of making an effort to continue enjoying the remainder of their lives.<br>Striving for a positive way of life – Focusing on improvements: “I know I’ve improved.” Participants accentuated the importance of focusing on improvements (across all areas of their lives, but in particular in communication skills) for living successfully with aphasia. As one participant stated, “I’m learning to speak. And that is good for me . . . I am relearning everyday. And that’s why I like—about myself.” Another added, “Success is . . . to see that I am improving. And I think that would be a major thing. And I know I’ve improved . . . I know I have progressed.” Participants compared their current level of communication skills to when they	Understanding their situation and acknowledging they will not be where they were prior to stroke thus accepting and moving on to look to the future positively – not comparing themselves to before

	first had the stroke to provide further clarification on this point: "When I had my stroke—two years [ago]—I can see how far I've come . . . a gauge . . . I had no voice and couldn't speak. Two years—I can talk properly"; "I feel quite . . . successful from where it started [when I first had my stroke]."<br>Striving for a positive way of life – Appreciation: "I'm still alive." For some participants, striving for a positive way of life was achieved through a sense of appreciation for their life and those things they still valued. A few participants expressed gratitude at having survived their stroke. One participant remarked, "I'm still alive," and another, when asked what made him feel like he was living successfully with aphasia, stated he felt successful "every day" because "every day's [a] new experience." Other participants talked about being thankful for what still remained in their lives and in some instances re-evaluating their priorities and learning to appreciate the slower pace of living: "Organising priorities in life"; "Before my stroke . . . deadline . . . quickly. And after my stroke . . . slow down . . . good."<br>Striving for a positive way of life – Trying new things and learning to adapt: "Try and do it." A further strategy participants identified to assist in striving for a positive way of life was trying new things and learning to adapt. Participants emphasised the importance of not being scared to try things (including things that were completely new to them, or trying to do things they had done previously, for the first time following their stroke.) "Try and do it," one participant suggested. Another said, "I have to take risks so I can get further on" and when asked to elaborate explained that "taking risks" meant trying things again, such as "Driving . . . One of the risks I had to take." Learning to adapt was described in terms of adopting a general problem-solving attitude or approach to things participants found difficult or could no longer do:<br>"You want something just gotta find a way around it (makes circular motion with arm)"; "If you think it's un—impossible walk around it." Participants also shared ways in which they had adapted specific activities. For example, one participant explained the significance of a photo of her iPod, by describing how she had found a way to still enjoy her love of reading by listening to audio-books: "I wead [read] with my ears." Finally, a number of other participants discussed how they had adapted by trying something completely new and different: "I have to [change my] ideas of life— I'll have to do something else, instead of what—I was a truck driver	Concentrating on improvement. Comparison to when they had their first stroke to how they are now.<br><br>Just knowing that they are still alive and appreciating this and embracing a slower pace of life<br><br>Trying new things and taking risk, understanding that this need to happen to continue to adapt and improve to reduce fear<br><br>Adapting hobbies or choosing different hobbies around their specific capabilities

	. . . that's out. Do something with myself . . . like forestry . . . camp[ing]"; "I wouldn't have thought about floristry until after my stroke . . . I was . . .working in the office before my stroke (referring to photo showing a flower arrangement)."<br>Striving for a positive way of life – Sense of humour: "I just laugh." A sense of humour was another internal personal resource enabling participants to strive for or achieve a positive way of life in order to live successfully with aphasia. Participants commented: "And I laugh. I just laugh"; "You can sort of take funny—funny sides of it . . . make a joke out of it"; "But laugh. You've got to laugh."<br>Striving for a positive way of life – It takes time: "Gradually." Lastly, participants noted that living successfully with aphasia and developing and maintaining a positive outlook is something that takes time. For some, there was a perception that over a passage of time things gradually got better: "Just gradually changing and gradually getting better." In other cases, participants described living successfully with aphasia as something that presented an ongoing challenge, and advocated taking a day by day approach: "I think you've just got to . . . take every day after the next one. . . Sometimes . . . you take three forward and then you drop back two. But then you sort of pick it up again. So it is . . . an ongoing problem." Another participant illustrated that in some instances living successfully with aphasia can be a journey encompassing large changes over a longer time period. He described himself as living "very successfully" with aphasia at the time of the interview, but shared how he had initially gone through a deep depression and contemplated committing suicide: "Depression . . . It's hellhole . . . it is actually the lowest you can get. There's people around . . . they were depressed. And they took their own life. I was in the same . . . thought about that . . . tough stage. But it's gone." Over time, a combination of factors, including medical management of his depression, support from his partner and others, and finding a new career path had enabled him to now perceive himself as living successfully with aphasia. His story demonstrated how participants' self-perceptions could change dramatically over time.<br><br>CORE THEME 4—Communication: "If I could speak right." Finally, communication was identified as the fourth major theme central to living successfully with aphasia. Participants'	A sense of humour was another internal personal resource enabling participants to strive for or achieve a positive way of life<br><br>living successfully with aphasia is something that presented an ongoing challenge or understanding that it's a gradual process of recovery

	comments about communication highlighted a strong emphasis on verbal communication, with all participants expressing a desire for improvement in their “talking” or “speech”. Participants differed in their focus when discussing communication in relation to living successfully with aphasia. For some participants, difficulties with communication acted as a significant barrier to living successfully with aphasia, while for others a positive focus on improvements in communication facilitated living more successfully. In many cases comments about communication were interwoven through the three other core themes (doing things, meaningful relationships, and striving for a positive way of life), revealing the integral role of communication in the broader context of participants’ everyday lives. Lastly, participants also identified many individual communication strategies that were perceived as necessary for learning to live successfully with aphasia.<br>Communication – Emphasis on verbal communication skills: “Talking.” Participants’ comments highlighted the relative importance of verbal communication (“speaking” or “talking”) over other communication skills for living successfully with aphasia. These participant statements illustrate this emphasis and provide examples of both a positive and a negative outlook: “Now I can . . . talk . . . That’s a very important thing . . . Being able to talk is the main thing”; “I get upset . . . frustrated . . . I can’t get words out . . . I want to say the things that I want to say . . . and I can’t get it out.” Verbal communication was thus identified as central to living successfully with aphasia, alongside the other major themes already reported. Many participants equated living very successfully with aphasia with complete recovery of verbal skills, for example, “If I could speak right . . . If I went back to normal.” A large number of participants expressed the desire for complete recovery of their “speech,” despite conceding in many cases that they knew this might not be possible.<br>Communication – The integral role of communication across themes. Noticeably, although there was an emphasis on “speaking” or “talking” as a distinct component of living successfully with aphasia in its own right, other comments from participants revealed that communication was inseparable from the everyday contexts in which it occurred. Communication played an underlying role across many areas of participants’ lives, which became evident through their statements related to other major themes already identified. For example, in the context of describing doing things, participants referred to the importance of a range of communication	Aphasia being a barrier to success in communication.<br><br>Verbal communication was identified as central to living successfully with aphasia<br><br>Communication difficulties made it difficult to achieve

	based activities, for example reading “newspapers” and “books”; being able to give a “talk” or “speech” at a church or community group; writing a signature “so that I can go shopping” or “at work . . . sending emails”. In many cases participants reported restrictions in being able to carry out activities because of communication difficulties. Communication was also interwoven through participant comments about meaningful relationships, for example, “talk[ing] on the telephone . . . [to] old friends . . . that’s very important” or “I don’t write to Mum . . . being able to write letters would be [good].” In particular, the strong impact of communication on the loss of friendships was evident, with one participant commenting on the loss of conversation with friends, “Even if it’s friends coming here. You don’t know how to (indicates to mouth) . . . they’re coming and say hello [but not the same conversation].” The underlying role of communication throughout the theme of striving for a positive way of living was likewise evident in participant transcripts, for example in the sub-themes of acceptance and focusing on improvements. Other examples can be found in the descriptions of the major themes already reported above. Communication was thus closely interwoven into the broader fabric of participants’ concepts of living successfully with aphasia, which encompassed doing things, meaningful relationships, and striving for a positive way of living. Participants’ comments suggested communication played an integral role across all of these major themes. Communication – Adoption of communication strategies for successful living. Finally, participants’ transcripts also revealed the adoption of communication strategies was perceived as a necessary part of living successfully with aphasia. Participants described a wide variety of communication strategies across a range of communication activities. These included strategies for improving communicative success when talking to others (for example, using circumlocution: “Give a meaning of that word. Many, many words to explain that word . . . paint a picture”; or using communication aids: “With iPod (referring to photo of participant with iPod). Got photos in it . . . So I can show you. I’m going to talk about my doggies or family”). Specific strategies for word finding were also described (for example, perseverance: “If you don’t get the word out keep trying”; and patience with yourself: “Instead of giving up when I can’t think of a word . . . Just a bit more patience . . . eventually get there”). In addition, participants shared how they adapted their environments to assist understanding—for example, captions on the TV, or “big books [large print books](description of photo taken at	participation in activities and tasks – barrier to achievement<br><br>Communication difficulties strain on social inclusion and relationships<br><br>How using different strategies for communication is needed for success<br><br>Adapting the world around them to aid their daily living

	local library)”. The strategies identified were often raised in response to the question about advice participants would give others for living successfully with aphasia, and reflected strategies participants believed helped people to live more successfully.	
3. Cross and Schneider, (2010)	Hope is a multidimensional concept that has a subconscious and ongoing impact on stroke recovery. It is a silent motivator that keeps individuals fighting and maintains their spirits. The novel finding from this study was that hope has an influence on the later stages of stroke recovery. Individuals up to 4 years post stroke remained hopeful for future improvement in both specific and general areas. The path of recovery among individuals was extremely varied, depending on the severity of the stroke experienced, individuals’ personal limitations, and their external environment. Hope, however, remained a constant factor for the majority of the participants. In terms of the general recognition of hope’s role in recovery, the majority of these women described hope as an unspoken necessity in life that is within everyone but that only appears when absolutely necessary.<br><br>“If you don’t have hope, there is no life.”<br>“If you don’t have hope you have nothing, it just gets hold of you and that’s it. You wouldn’t have anything to fight for.<br>“What would you do without hope? It’s a thing that you live with.”<br><br>When faced with a sudden life-altering event, such as a stroke, participants alluded to the fact that hope was the backbone to their ultimate recovery.<br><br>“[Hope has to be the backbone.] It has to be, because if you have a negative attitude you are not going to get better. You are just going to dwell on your problems and they will get worse.”<br>“Well yeah you got to have hope or you got to have that inner feeling that you are going to work for it.”<br><br>Whether reflecting on past hopes or thinking about future hopes, the majority of the participants shared the common thought that hope was a necessity. Women who had	Identified importance of having hope and the detrimental effects of life without hope<br><br>Recognising that having a negative attitude and no hope will impact recovery

	experienced more severe limitations found it harder to remain hopeful for future recovery. Without progress, participants stated that hope eventually goes "by the board."<br>"You lose hope because you can't get better. You know you can't. So it goes by the board."<br>"Hope is something, you want to hope for the best for yourself and for people around you. But sometimes it can't be done.<br>One prominent theme among those women who had apparently lost hope was acceptance. Although the loss of hope is often associated with depression, these women described an acceptance of their limitations.<br>"I am happy as can be. There is nothing more that I can do so I put up and enjoy life."<br>"I guess I just accepted it, because you cannot do anything about it, I guess I had to accept it."<br><br>Acceptance was an attitude that participants developed over time. After experiencing periods of depression, these women tried to refocus their thoughts and be thankful for the abilities they retained.<br>Factors influencing hope<br>The main aim of this study was to determine the factors that influence the perceived hope of individuals in the later stages of stroke recovery. Through the use of a background questionnaire<br>2 hope scales (HHI and GHS), and a semistructured interview, we sought to gain valuable insight into the perceived role of hope in recovery. Three factors emerged from the data and were found to have an interactive influence on the perceived hope of these women. These external, internal, and personal factors were described as influencing hope along the illness trajectory.<br><br>External factors<br>Medical support systems, rehabilitation programs, and other stroke survivors were all described as playing an integral role in influencing hope and fostering recovery in these women. In the first few days, weeks, or months following the stroke, stroke survivors are constantly relying on others to guide them through the recovery stages and to help them	Loss of Hope narrative due to inability<br><br>Stroke survivors who accept their limitation present as happier due to less disappointment

	adapt to their new life circumstances. As they move into the later stages of stroke recovery, they still needed a social network of support to sustain hope and foster further recovery.<br>Support systems. The importance of support systems in sustaining hope in individuals has been frequently documented in the nursing literature. Support systems include medical staff, rehabilitation therapists, family, and friends. They are an intricate network that work together to motivate, inspire, guide, and care for the stroke survivors.<br>"I felt quite hopeful because the doctor kept telling me it was hopeful. I think that counted for a lot. I know that's a pretty important aspect of it all, if you have that kind of supportive care."<br>"I think knowing I have such support. Just knowing that if I am having a problem all I have to do is pick up the phone, so you're not alone."<br>"[My granddaughter and friend] give me hope."<br>"[My husband] is always there for me."<br><br>All participants felt that this continuous feedback and constant care (in the hospital and after discharge) were important for sustaining hope in the short and long term.<br>Rehabilitation programs. Stroke survivors often rely on rehabilitation programs to minimize their impairments and maximize their ability to regain independence. The majority of the women in this study participated in a rehabilitation program after their stroke. The duration and type of program depended on the individual's limitations and needs. Some participants had therapists come to their homes, whereas others attended rehabilitation facilities. All participants valued their time in the rehabilitation programs and commented on the positive impact that their therapists provided.<br>"When you have a stroke so many things happen at once that you are incapable of doing so much and when you do follow the advice of your therapist then there is always hope that things are going to get better. Every time I came away from a session I always felt better."<br>"I learned [through] going to therapy…that you have to keep going. You can't give up or…you'll just waste your life." Participants felt that rehabilitation programs not only helped with their physical functioning but also provided psychological and emotional support. Other stroke survivors. Other stroke survivors have a prominent influence on an individual's level of hope poststroke. Participants who were having difficulties coping poststroke relied on other	Importance of support systems including emotionally, motivationally and physically supportive networks.<br><br>Highlighting the benefits of Therapy as a driver for hope. Helping with physical functioning, psychological and emotional support.<br><br>Relying on other survivors for motivation and a symbol of hope. Viewing others situation and

	survivors for motivation and a symbol of hope. Interactions with other stroke survivors was a reality check for those individuals who were recovering quickly, as they provided a comparison for their own rate of recovery. They felt hopeful that things would continue to improve over time.<br>“To see other people come into the hospital and in a week they are up and walking. I said, ‘Now why shouldn’t I?’”<br>“I have 11 siblings and 8 of them had strokes and I am determined that…I won’t be the only one who is not completely [recovered].”<br>“You realize what could have happened and didn’t. You are hopeful that you will get over what did happen.”<br>“You see other people with devastating residuals and you think to yourself, God, I am very fortunate. I felt I was doing so much better than other people that were in there and of course when you get out, then you feel very hopeful because you think well you know they wouldn’t be letting you out if you weren’t doing alright.” Other stroke survivors tended to have a more pronounced impact on hope during the acute stages poststroke, when individuals were hospitalized or in rehabilitation settings and therefore were in contact with other stroke survivors. Depending on the individual’s speed of recovery, other stroke survivors may have played a role into the later stages of stroke recovery.<br>Internal factors A stroke is an individual experience and has a unique impact on each person. Once a stroke has occurred, it is up to the individual to take control of his or her own recovery. Without a positive attitude and self-determination to get better, an individual may fight against progress. All of the participants stated that their hope and motivation to recover came from deep within themselves. In some cases, their spirituality and sense of faith were also described as having an influence on recovery. Determination. Many participants alluded to their determination as a major influence of hope. Their relentless will to keep fighting despite setbacks inspired their sense of hope for the future.<br>“Just determination, the fact that there [are] people there to help you recover to some extent and the hope that you will regain some level of normalcy.”<br>“Subconsciously, my determination has helped a lot, that I’m going to do it. I’m going to do it.”	comparing themselves against them.

	"I think we are given this determination to survive no matter what. Self-preservation. It's pretty. That's what puts us all together you know our belief in living. Our, I mean we are living organisms so that drive to survive no matter what has got to be there." Many participants described their determination to fight for recovery as an ingrained part of who they were. Their determination to survive despite adversity had grown over time. Positive attitude. All of the women in this study described themselves as having a positive attitude. They expressed the notion that their attitude was an innate characteristic that was with them long before the stroke. Many described that this hope and a positive attitude have an interchangeable effect by fostering good things for the future.<br>"I guess it's just me.<br>"In the beginning you know when you have it and you get these down and out crying spells…I myself have had an attitude…Thinking that something can be done that I can do so [that gave me hope and influenced my recovery]."<br>"I always think it's bad to look at negative things or to feel sorry for yourself. I've never been like that. I have never felt sorry for myself. I always know there are so many people [who are] a 100 times worse off. I'm a very hopeful person with other people too and I try and build that up in my family. Don't give up you know." In terms of the interchangeable connection between hope and a positive attitude, these women made the following comments: "It's your attitude. It's hope. It's a lot your attitude."<br>"I equate [hope] more with a positive attitude and I have been positive all my life."<br>"Even without a stroke you have to have a positive attitude towards things. I guess you have to ask for the grace to be able to accept these things that fall on us." It was evident that these women felt that their positive attitude was part of who they were and that this attitude was interchangeable with their sense of hope. Spirituality. There was a mixed response in terms of the role spirituality played in giving hope and fostering recovery. Approximately half of the participants stated that spirituality was not a major influence on their hope and recovery, whereas the other half felt it had played a prominent role. Two of these women were nuns, and spirituality and faith were a dominant influence in their lives.<br>"I just know that with the belief in God that he will make things better has made my life a lot easier."	Positive attitude driving the hope narrative and recovery<br><br>Turning to spirituality for hope, power greater than themselves and trusting in this

	"You pray for things and hope that the Lord will hear you and of yourself. We can do nothing but with prayer and hope, it all helps along."<br>"I guess my faith is at the bottom of everything. I have always been a Christian since I was a child. I have never waivered in my faith….You know like they say you have to have blind faith and that's what I have blind faith. I know that you can't prove everything. But I don't expect it to be proven. But the blind faith has really kept me going." The impact of spirituality on recovery is dependent on the individual and, therefore, its impact on hope depends on the individual's beliefs and practices.<br>Personal factors<br>Personal factors depend on an individual's personality and surroundings. These personal factors include progress, goal setting, being active, and awareness. These factors influence an individual's participation in recovery and the pursuit of external resources to aid them in the journey to a successful recovery.<br>Progress.<br>Personal progress is a key ingredient to sustaining hope. Small improvements in recovery foster hope for future long-term recovery. Past progress also has an effect on individuals who are several years post stroke. By reflecting on the beginning stages post stroke and progress they had made, participants were hopeful for continued recovery in the future. Some described the importance of small improvements.<br>"I was always a person who had hope, you know. It acted out for a while, you know, a stroke until you get to feel a little bit better and that there was some hope and when you get to get some strength back in your arms and your legs and when you are able to do a little bit more it gives you [hope]."<br>"When I was in therapy…there were some of those tests, I couldn't do them. You know, I just didn't have the strength. And I remember thinking, am I going to get over this? And of course the therapist would be there saying, 'Well, try it again, try it again'. And so you try again and then you realize, yeah you can do it. So, I think that every bit of that is more hopeful." Over the long term, these women described how these incremental improvements assisted them in their process to regain a sense of normalcy in their lives and to return to previous activities.	Looking at where they have been and what they can do now, ability to reflect and recognise recovery. Personal progress and hope

	"When things change and I have a more positive attitude as I think things will change for the better for me."<br>"I'm back to doing everything I did before. I guess it feeds into sustaining your hope." Personal progress was a crucial factor in sustaining hope. When an individual's progress began to diminish, hope was more likely to be lost. Participants alluded to the fact that large gains of progress were not necessary and that every small step of progress helped maintain hope<br>Goal setting<br>Hope generally involves setting and working toward a goal. Contrary to past studies, goal setting was not a major influence of hope for the majority of these women. The women expressed the importance of taking it day by day and being happy with any progress they were making.<br>"No, I just went day to day and did what I was told."<br>"I just take every day as it comes and I make the best of every day." One woman described goal setting as a strategy she used in her rehabilitation. Her attainment of these personally prescribed goals has continued to have an influence on her life and her recovery.<br>"I guess that's where the goal comes in. You have a goal and you have to be positive that you can reach that goal." Although goal setting was not a conscious practice for many of the women in the study, they did describe hidden long-term goals on which they worked daily.<br>Being active. All participants expressed the need to keep active. By participating in social activities and interacting with others, these women kept their minds stimulated. Some participants also expressed the need to help others.<br>"Oh yeah and of course you are not thinking of [yourself]… you are not pitying yourself. You are out there helping."<br>"You have to keep your mind off of yourself. You know if you are always thinking about yourself and pitying yourself." In helping others, these women gained a purpose and a sense of accomplishment, which inspired hope in themselves and in those they were helping.<br>Awareness. Participants expressed the need to be informed of their condition and progress. Knowledge about what to expect after a stroke and what steps needed to be taken gave these women both comfort and a sense of hope.<br>"And that gives me comfort. If I know what to expect, then I am okay."	Progress was not recorded by achieving goals but instead achieving and living day by day.<br><br>Need to keep physically active and their brain active with activities and hobbies<br><br>Caring for other to distract from own problems and regain sense of purpose

	“People are not hopeless, they feel hopeless so it is a feeling so a feeling is something that can be altered. You know, we can feel sad, but we are also capable of feeling glad, you know….So if you feel hopeless we also have the possibility of feeling hope because one is the other you know. But what makes that difference is the surroundings that one’s in, being aware that there are options open to you. That’s not to say that it’s easy.” By being aware of their personal condition and the resources available to them, participants were able to tailor their rehabilitation program to best fit their needs. By knowing that there were resources out there and that recovery was a possibility, hope for future recovery was inspired. Overall, these 3 factors (external, internal, and personal) worked in conjunction to inspire hope for future recovery. Without a supportive environment, belief in oneself, and belief that progress was being made, individuals were more likely to lose hope. It is therefore important that everyone involved in the stroke survivor’s life work toward providing the optimal environment for maximal recovery.	Utilising a supportive environment of support, belief in progress and self to optimise hope and recovery
4. Fotiadou et al., (2014)	Aphasia<br>The impact of aphasia was a consistent thread running through the blogs, affecting interactions with all parts of a person’s network. It made it harder for a person to have in-depth conversation with family or friends and express opinions and thoughts. The language difficulty could also make a person feel less included in conversation. Conversation continues one way because of my aphasia. (Nick) There was also a sense of not being able to get across their message in a timely way, impacting on a person’s ability to make humorous observations and jokes. This is illustrated by Laura, who described a car journey with her husband, where they were listening to a radio programme:<br>One of the frustrating things about aphasia for me is the lost opportunities to make snide remarks. I’m too slow … I couldn’t say it [about the radio presenter] but I wanted to say: a southern drawl is nice, but she sounds like a southerner on a big dose of tranquillizers […] [Husband] was about to turn off the car. I was still trying to formulate a witty remark. (Laura, 48) Participants also described finding it difficult to follow conversations, particularly where	Aphasia affecting ability to include humour during social interactions and family interaction affected

	multiple conversations where taking place at once. Adam, 56 years old, discussed how hard it was to follow what was being said when several close family and friends were talking together: “Bit of bother trying to keep track of all the conversations.” (Adam, 56) A prevalent theme through the blogs was that the ongoing difficulties in communicating resulted in feelings of anger, frustration and depression. As noted by Rose, 55, “Being in a world where you can’t communicate to others is hard too.” The changes in social interaction caused by the aphasia led many participants to feel they had lost a part of their former selves. They could feel unsociable, anxious and inclined to avoid crowds. Tom described the impact on his sense of self in no longer being able to communicate with others as he used to:<br>We have lost the use of words, and thereby a piece of ourselves. […] For someone who used words for 40 years, it completely changes my life. (Tom, 67) Yet, despite the difficulties, participants also found ways to adjust to their aphasia post stroke. These included expressing gratitude to still be alive and still able to “kiss my family, children and boyfriend.” (Beth, 42)<br>We are all different. One individual is not necessarily better than another. He or she may just be different. […] Aphasia is not the end of the world. I am still alive and I am still me. (Tom, 67)<br>Impact of stroke and aphasia on social relationships<br>The blogs described the everyday experience of living with aphasia. A number of core themes emerged from the analysis: the impact of the stroke and aphasia on family, friends and the wider community; support received and responses of the social network to the aphasia.<br>Family Changes in activities. Participants reported restrictions in their ability to participate in family activities, such as swimming or playing games with their children. They also described limited options in terms of places they could visit, for example, not being able to go further than the local shop. Another common theme was that activities required more time than prior to the stroke. Feeling tired when outside was another barrier. This is illustrated by Beth, 42 years old, who found it difficult to accompany her son: “I am so tired, the fatigue is getting better, but I went two college visits with my son over the weekend but I am no spring chick any longer … so these were very long days for me! […] really tired me out.” Feelings of unhappiness resulted from the lack of ability to participate in family gatherings. Adam, living with his wife, described how sad he felt when not able to visit his daughter: “Even though Daughter Mine only lives 6 mins away, today it’s a long way to go. I’m just not strong enough to walk that far	Social isolation due to lack of communication ability.<br><br>Concentration on what can be achieved still, adapting communication.<br><br>Reduced physical function reducing assess to family activities and reducing independence<br><br>Fatigue being a barrier and the effect on family participation

	today. Still made me feel awful for not going." While many of these restrictions stemmed from the stroke, some were a direct consequence of aphasia. Laura, 48, illustrated this point as she described struggling to read to her 5-year-old daughter at bedtime:<br>Last week she (daughter) wanted me to read The Three Little Pigs, but it's hard to be convincing when the evil wolf sounds like this: me: 'Little Pig, Little Pig, let me out!' [daughter]: 'You mean, Let me in'.<br>Changes in family dynamics. Feeling dependent on family members was frequently raised. Participants tended to rely on others for a number of activities of daily living, such as preparing meals, as well as for compensation of lost skills like driving and talking. This often meant that another person would have to accompany them for a significant part of the day. They could therefore feel that they were losing their independence.<br>As I'm not 'allowed' out alone (could you imagine that?—I wonder what they think I'm going to do?). (Adam, 56)<br>Having to rely on others, and the loss of autonomy this represented, was described as a source of worry. Carl, 69 years old and living with his wife, explained: "[wife] showers me Sometimes weekly. Sometimes less … These are the kinds of self-imposed problems that drive me crazy too!!" Participants described feeling that they were losing their role in the family. They described no longer being able to take on past responsibilities, such as carrying out household chores. There was also the sense that however supportive a spouse was, still there was a shifting of the terms of the relationship, as discussed by Laura: "[…] partners can share some of the burdens, but we have such different journeys to make. Now almost a year has gone by, and [husband] and I are still adjusting to our new shared reality." It could also lead to a change in the husband–wife dynamic in terms of decision-making. Emily, 42, reported that she could not voice her opinion regarding an important operation due to limited expressive abilities. This forced her to leave this decision to her husband:<br>[husband] is the physician in our family, I leave the decision to him. And thus I put him in such a difficult position […] he has to decide about my future.<br>Parenting limitations and reduced parent–child interactions owing to communication difficulties were described. An example is Emily, living with her husband and child. Prior to the stroke, she was the one mainly responsible for her 3-year-old's upbringing. Post stroke, her	Change within the family dynamic and loss of autonomy. From family member to carer<br><br>Loss of identity and role with further loss of partner dynamic<br><br>Loss of role as a parent

	husband became the main carer. “Up until now, he [her son] was truly a mama’s boy […] Now [husband] is the playmate, the reader, the explain it all person.”<br>Friendships<br>Friendships emerged as an important part of people’s lives. Friends were referred to with great appreciation: “They are my rock and my true north” (Jess, 31, talking about her friends). Nevertheless, following stroke and aphasia, some participants reported difficulties in maintaining their pre-stroke friendships. Reduced contact and changed preferences. The main trend post stroke was for participants to be in less frequent contact with friends. The reported explanations included mobility issues that made travelling difficult and difficulty using technology or phones, in part due to aphasia. An example is Adam, whose contact with his best friend reduced post stroke:<br>I’ve not seen him [his friend] for a year, mainly because neither of us travel well, though we’ve kept in touch by phone I have to admit I’m not too good with phones, so the contact has been down. (Adam, 56) Physical disability also made other aspects of social gatherings problematic, for example, using cutlery in a restaurant, as described by Pam, aged 29.<br>When I’m on a date at a restaurant, I may want to order a piece of meat—steak, chicken, etc. But I don’t, because cutting meat with one hand is a pain. Miss Manners says, ‘Asking your date to help you cut up your dinner is bad form’. (Pam, 29) Some participants described receiving visits by friends rather than initiating visits. Reduced energy levels, tiring easily during group meetings and struggling to follow group conversations resulted in a preference for meeting friends one-to-one for some participants, as discussed by Emily, 42 years old: “I am not ready for larger gathering, I tire easily and when there is more than one conversation my speech suffers. So we meet one-by-one with a great many of my closest friends.” Aphasia confounded by environmental factors, such as background noise, could also present barriers, as discussed by Jess, 31 years old: “My fear is when I go out with my friends and they put on background music, or suggest a late night game of Articulate … I can rarely understand the questions, let alone reply.” Participants also described feeling embarrassed of their aphasia. This impacted negatively on their willingness and ability to socialise. For example Beth, whose aphasia was initially severe, avoided friends as she felt uncomfortable socialising: “I was embarrassed by my speech […] I hid from friends while healing”. Participants also described	Importance of friendship for support and struggle with maintaining all post-stroke friendship<br><br>Adapting to maintain friendships by having one-to-one meeting instead of big groups

	feeling nervous about socialising and worried about other people's reactions: "I am anxious .I don't know why but I am anxious about seeing my friends. Some how I fear that when they see me, they will feel sorry for me." (Emily, 42) Over time, these responses evolved and changed, however, as shown by one participant, Beth. For Beth, as her aphasia improved, she gradually started spending more time with her friends: "since speaking better […] I now actually spend 'real' time with my pals." She described developing a new approach towards friendships: "I am liking more and more meeting new people."<br>Wider social network Participants' ability to interact with the wider social network also appeared to be affected by stroke and aphasia. Wider social network in this study was conceptualised as consisting of neighbours, co-workers and community members.<br>Reduced participation. Simple, everyday communication was often described as a struggle, and participants described times when they felt unable to take part or communicate in their community due to their aphasia. Laura, for example, described her difficulties communicating with her neighbours post stroke: "a crowd begin to form in front of me, as if the oracle was waiting to speak. But this oracle has aphasia!" For some participants, their aphasia meant they avoided social interaction, for example, a casual conversation with someone new in their local park. Reasons given for avoidance included difficulties talking and fear of not being understood. This is illustrated by Nick:<br>[…] staying away from people because I can't talk. I would have to explain I've had a stroke and can't speak properly […] So I avoided folk in general. Rather than go out and engage in his former hobbies, Nick described how post stroke he preferred to spend time alone.<br>Environmental factors, such as using public transport and bathrooms and eating and drinking in public, were also perceived as barriers that discouraged contact with others. Adam, 56, described the difficulties he encountered when someone asked him for support in a hearing and then suggested going out for a coffee afterwards:<br>For me going out is a carefully planned military style operation. I don't—can't go out unaccompanied. […] We need to know exactly where the buses are and how long we would have to wait for them. […] Don't get me going on trying to catch a bus in a wheelchair. While maintaining a blog was a way for people to reflect on their experiences and potentially communicate these to others, nonetheless sustaining a blog was described as a challenge.	Embarrassment and anxious of speech being a barrier<br><br>Accepting their condition and building new relationships and social networks with people who understand condition<br><br>Impossibility of achieving by acknowledging limitations and not challenging them isolating themselves.

	Fatigue, struggles with typing and language hurdles could make completing entries time consuming and effortful. Completion of a single post could take from half an hour to 3 days. The consequence of experiencing these everyday barriers to participation was that some participants expressed feelings of frustration and anger. Pam, who was 26 when she had the stroke, described how someone "[…] recently asked me if I was angry. The correct answer is 'Of course'.[…] I have had to put my life on hold?" Work. Going back to work was difficult for most of the blog authors in this project: post stroke only Beth and Jess were in employment. Factors which made it difficult for participants to return to work included aphasia, fatigue, older age, epilepsy, short attention span and difficulty multitasking. These post stroke sequelae also made searching for a new job challenging.<br>Aphasia, fatigue and age now preclude me from working at those jobs that I loved and enjoyed. (Tom, 67) For some, loss of work also entailed loss of the social contact which went with the work environment. New and positive social interactions post stroke. The onset of aphasia motivated some participants to become active members of their wider community, including participating in aphasia, stroke and religious groups, such as aphasia symposiums. Emily, 42, described how becoming a member of an aphasia group enabled her to develop new friendships: "After just one session with her we understand that we are going to become best friends."<br>Entering the social media community, for example, through blogs, Facebook and Twitter, was another new social avenue for some. Jess, 31, viewed blogging as a fun activity during which she could be herself: "I've learnt to just be me in a blogging sphere. It's a nice safe cyber world." A factor which appeared to promote participation was the person's willingness and motivation to socialise despite their difficulties. Beth, a mother of a 5-year-old, describes her eagerness to socialise with other parents: "I love going to the games and I am learning to socialize with the families, which I struggle a bit […] but I'm taking baby steps to know the [surname] parents."<br>Support received<br>A theme which came up consistently was the different types of support from family members, friends and the wider social network provided to participants, and the value this held for them.	Going to work was their identity and without it they are lost<br><br>Accepting condition allowed stroke survivor to open new door to wider communities and acceptance within their new role and identity.

	Practical support Family members assisted in activities of daily living, like having a shower, preparing meals and helping the participant manage their finances. They also assisted the participant with speech and language exercises and playing word or maths games. Friends provided help such as dropping off dinner or babysitting. There were also examples of friends becoming informed about aphasia and raising money for financial support.<br>I was invited to a regular Coffee Morning event, where they surprised me with 1000 origami cranes and a check of over $3000. (Emily, 42, talking about her work friends) Another way in which friends provided practical help was in seeking specialist support. Carl discussed: “[friend] got a psychiatrist to see me.” Practical support from community members was also described, such as delivering shopping free of charge or helping to shovel snow off the garden path. Help was particularly appreciated in emergency situations: Rose, 55 years old, recounted how a woman in the church aided her during a seizure: “She put her arm around me and guided me to the hall.”<br>Emotional support<br>Participants described how family made them feel protected and loved, provide reassurance and encouragement and created a safe, tolerant and supportive environment for them.<br>My mom—my angel through my whole stroke survival. […] I would not be doing so well if it was not for her. (Beth, 42) Friends also provided valued emotional support. Jess, 31, described her friends as her “guiding stars.” Pam, 29, described her friend’s encouragement in the lead up to brain surgery: “Before I had brain surgery, my friend [name] offered to shave her head in solidarity.” On occasion, members of the wider community also provided compassion and encouragement, for example, praying for the participant every Sunday at church.<br>Beth also described the sensitivity of work colleagues, who gave her the space and time to do things for herself, so that “[…] have made it possible for me to lead a positive life through my recovery.”<br>Other people’s responses to the stroke and aphasia<br>Responses of family members<br>Positive reactions reported were being supportive and tolerant and accepting the “new person” with aphasia. Additionally, participants spoke of new family jokes inspired by their aphasia.	Recognising the plethora of family support, orchestrating people to help for different aspects of life<br><br>Using humour as a facilitator of social

	"Now my family is getting some good-natured family laughs over some of my speech". (Laura, 48) Negative reactions from family members included lack of patience, sadness and rejection. Emily, 42, commented on the negative response of her 3-year-old son after the stroke. Prior to the stroke, she described how she and her son were "inseparable." On returning home from hospital, however, she wrote: "he doesn't want me in the room again. When I try to lie down he pushes me out of the bed, he clings to his dad." Laura described her son's sadness that she had had a stroke. When looking through old family photos, her 17-year-old son remarked: "I wish you didn't have a stroke. […] You were more fun then."<br>Responses of friends and the wider community Although there were many examples of positive and supportive responses, there were also some more difficult reactions described. Participants felt at times that they were being labelled as disabled, with low IQ, diminished and pitied, resulting in feelings of anger and frustration. For example, Jess described her difficulty participating in a game due to fatigue, leading others to assume she was not intelligent which she found upsetting: "And the FRUSTRATING thing is that is that people think it's because you have a low IQ, which isn't the case." Strangers could also assume they were incompetent and unable to take part in activities.	interaction and inclusion<br><br>being labelled as disabled causing frustration and upsetting impacting on motivation and happiness
5. France et al., (2013)	Quest Memoir<br>Four women and 4 men aged 54 to 85 years, who had had a stroke a few months to 6 years previously, presented accounts with a dominant quest memoir narrative. These people had chronic impairments from their stroke and most had significant functional deficits, such as severe aphasia or partial paralysis. Men's and women's quest memoir narratives were very similar in content. The main characteristic of these narratives was a relatively upbeat presentation of adaptation to stroke-related changes to their life, as illustrated here:<br>This thing has happened to me. . . . I just want to get on with my life and . . . I can't turn the clock back. I've got what I've got, go and deal with it and run my life accordingly. (James)<br>Adaptation took various forms within the interviews, including an apparent acceptance of impairments, looking for new occupations or interests when old ones could no longer be sustained, and/or finding new ways of doing tasks. For instance, Sandeep still led an active life, traveling long distances abroad and doing voluntary work, but he had started to reduce his	Accepting their situation and utilising what they have.<br><br>Narratives of adaptation and knowing limitations and managing these.

	commitments: "So I try to see that I still contribute. I do not go to so many meetings. I only go to the meetings where I could contribute something. I don't go just because my attendance has been ticked."<br>Three respondents in their 70s and 80s mentioned their age in explaining their response to their disabilities. They reasoned either that illness and disability are expected with greater age or that their losses were less problematic since they had retired and did not need to earn a living or raise children:<br>[The nurse] said, "Oh you've got to realize," she said, "your age and you see, you were very fortunate up to now. You've never had any serious illness up to now and plenty of people had." "Well," I said, "that's true enough," I said, "I can't grumble." I didn't have, and at my age I've been very fortunate. It's the only way to look at it. (Helen)<br>Some men and women also presented their experiences in a stoical way to avoid being seen to complain about their situation. For example, Janet, whose father had also had a stroke, said, "He's had a lot of problems, my dad, you know. So, but he just grins and gets on with it [laughs], and that's what I'm trying to do." In addition, most were grateful to have recovered significantly since their stroke, which appeared to aid their acceptance of any remaining limitations:<br>There's things I can't do, I'll never be able to do again, I know that. But I look upon it, I was lying in my bed after my stroke, the list of things that I could not do was a great big list and the list of things I could still do was very, very small. So over the last year, I've transferred things over from "can't do" to "can do," and the lists are beginning to settle up. (James)<br>Commonly, these narratives contained reference to feeling lucky compared to others who had been more seriously affected by stroke. Two exceptions were Gerry and Karen, who had not experienced significant recovery; Karen felt her ongoing impairments were fairly minor, whereas Gerry still had severe aphasia. Some people seemed to have come to terms with the consequences of the stroke partly because of what health professionals had told them, for example, about what level of recovery to expect, as Janet explained:<br>I love my driving and I drive twenty-six miles to my work, and he [the doctor] says, "You'll, you'll not drive." He says, "I don't think you'll be able to go back to your own work," he says. . .	Recognising the positives from the situation and acceptance.<br><br>Using humour as a coping mechanism<br><br>Patients feeling grateful to their recovery by recognising where they were and where they are now.<br><br>Confidence in the health professionals – trust

	. It's the best way to get it, I suppose. If you're going to get any kind of news you're better getting straight, aren't you?<br>Still being able to participate in previously enjoyed activities, even if done differently, and to feel valued, were both important to people's positive attitudes. Sandeep, for example, had been given an award for his voluntary work and Karen felt influential in her new job: "I think I've made a positive contribution to the company as a whole." Often regret for what they had lost was apparent, but the overall tone was positive and accepting. Religious beliefs had also helped some people to come to terms with the stroke's impact. Overall, maintaining or finding purpose in life was an important part of (apparent) acceptance and adaptation to the effects of stroke for these 8 respondents.<br><br>Restitution<br>Only four accounts (2 women and 2 men aged 66 to 84 who had had a stroke 1 to 7 years previously) fitted a dominant restitution narrative. These accounts displayed a belief that the respondent was fully recovered or would fully recover: "I was very, very pleased about it all, especially now, a year later, when I feel perfectly all right. Perfectly normal person" (George). Anne and George had had "mini strokes" or transient ischemic attacks (TIAs), which had been surgically treated; Audrey had had a major stroke. George and Audrey believed they had recovered abilities initially affected by the stroke, such as vision and speech. Anne had been told to anticipate a full recovery. These three did not view their stroke as a chronic illness. Lenny had more serious impairments, saying he initially "couldn't walk or do anything." He still had weakness on the left side of his body, walked slowly using a walking stick for longer distances, and lived with his brother for practical support. Nonetheless, his account displayed the characteristics of a restitution narrative: "I had a belief that I am cured. . . . I'm better now. I could say one hundred percent better now." His considerable recovery, his limited day-to-day activities, and the fact that he had lived with the impacts of stroke for 7 years could explain why he felt recovered despite continuing limitations. Alternatively, he might have wanted to present himself as a recovered and capable "very strong person" in line with the culturally preferred restitution narrative. His reference to his strength hinted at the possible influence of his masculine identity in his use of the restitution genre.	Ability to undertake some pre-stroke activities<br><br>A feeling of worth and identity within an environment<br><br>Spirituality and religion helped with coping and acceptance<br><br>Not viewing their situation as a chronic illness and that recovery was inevitable<br><br>Feeling recovered even still with limitations highlighting acceptance and identifying the new person he has become being the normal

	Chaos<br>Three respondents (2 women and 1 man aged 47 to 76 who had had a stroke 2 to 5 years previously) presented accounts that predominantly conformed to chaos narratives. However, two of the narratives (Jenny, Andrew) were coherent rather than chaotic in form. Therefore, the lack of hope and depression they expressed might fit better with a new “despair” genre. Nasreen’s account was somewhat chaotic in structure, although this could be partly because a freelance interviewer who spoke Punjabi carried out the interview. All three respondents experienced severe permanent impairments affecting their mobility and ability to walk that limited their abilities to carry out social and day-today activities independently. Andrew and Jenny also described some cognitive impairment. They all had extreme difficulties adjusting to their losses, as illustrated by Jenny’s description of the impact on her and her husband’s life:<br>“Both of us have found the effect of the stroke absolutely devastating because we were, you know, we were starting to do things. Like we’d started going on walking and dancing holidays and we were starting to do things like that that we found really, really enjoyable, and I suddenly can’t do them anymore.”<br>A common theme in these chaos or despair narratives was feeling out of control of daily life and dependent on family members to perform even basic tasks. Andrew felt he had lost his role as a man and a husband within the family, and was worried his wife might leave:<br>“Well, it certainly changes things in terms of your role within the family because certainly now I’m dependent now [rather] than a partner. That’s certainly how I feel, because the things we’ve taken responsibility, the things that need being done in the house, just paying bills, organizing things, just running a home, all those sorts of things, all the responsibility of doing that falls on my wife, which I don’t like and I feel, “What sort of a husband am I?” It does worry. I do sometimes think to myself, “What’s my wife getting out of this relationship and how long are we going to be able to sustain it?”<br>Similarly Jenny’s husband had to take on household tasks that she had previously performed, such as grocery shopping and cooking, which had caused conflict between them. Thus, Jenny’s and Andrew’s accounts seemed to provide some evidence that stroke had disrupted the more traditional gender roles in their married lives. Nasreen emphasized the impotence she felt. This	Thinking back to their previous function as a barrier to what they could achieve.<br><br>Now feeling lack of hope and desperation<br><br>Loss of identity and masculinity as head of the house hold.<br><br>Needing heavy assistance from family putting straight on social relationships

	was compounded by her inability to speak English, which prevented her from communicating with and receiving information from health professionals and social workers:<br>Nasreen (N): I can’t straighten my fingers; my hand won’t straighten. Interviewer (I): Can’t you ask them [health professionals] to help? N: How can I ask them? I: Can’t your sons ask them? N: Sons can help, but they won’t take me to hospital. (Interview translated from Punjabi)<br>All three respondents were taking antidepressants, and Jenny and Andrew explicitly referred to being depressed: “I do often feel very suicidal because I’m finding it impossible to think of things I can look forward to except a game of Scrabble and that’s not always available” (Jenny). They also described significant emotional suffering, including as a result of their health care experiences. Andrew, for example, related his traumatic and disempowering experiences in hospital after the stroke: “I did feel very, very alone. It would have been nice to have thought I had a friend in the hospital somewhere, other than my visitors that were coming in to see me.” Nasreen had been told her recovery would be slow, whereas Jenny’s and Andrew’s health professionals had left them with no hope of recovery. None had experienced much, if any, improvement since their stroke. The severity of their disability and the unlikelihood of improvement could explain why these three accounts largely conformed to chaos narratives.<br>Interviews that Lacked a Dominant Narrative Genre<br>No one presented an account consistent with a dominant quest manifesto or quest auto mythology genre. Some women’s and men’s interviews contained elements of quest manifesto, but only Marie’s account contained elements of quest auto mythology. Marie was in her 40s and had suffered from central poststroke pain since her stroke 3 years before. Her account was almost equally dominated by the often contradictory use of restitution, quest memoir, and chaos genres, but she did not use quest manifesto. She repeatedly presented herself as a capable, committed single mother who was striving to cope simultaneously with her impairment and motherhood. Overall, Marie appeared to be trying to present a quest memoir narrative while really wanting a cure (restitution narrative). She corrected herself on a couple of occasions to give a more positive, optimistic presentation: “I’m sorry. I’m not supposed to get upset, am I? I’m supposed to be being positive and helpful.” She then slipped back into a description of daily life more in keeping with a chaos genre: “My social life’s completely changed. My work life has changed. In effect, it, it’s blown my whole world apart.”	Medication as a coping mechanism for depression<br><br>Disempowered and alone after stroke<br><br>Managing role of a parent post-stroke

	This strongly suggested that Marie was aware that chaos is not a preferred public narrative type. At numerous points throughout the interview she expressed hope for a miracle cure for her pain, in line with a restitution genre. She also used powerful imagery to describe her personal transformative process, thus conforming at times to a quest auto mythology genre:<br>I feel as if it's my second chance now. It's as if I had one life and it ended when I had the stroke, and I now have a completely new life. . . . I feel like I've been through a baptism of fire and come out at the other end . . . crawled out of the other end in effect, and I've had to claw my way back to health as I knew it before.	Want to be portrayed as succeeding but is struggling to come to accept new identity<br><br>Recognising new identity and the loss of the old
6. Glintborg & Krogh., (2015)	The First Meeting<br><br>The first author meets Mette for the first time during spring 2013. Mette has suffered a stroke just three weeks earlier and is now being observed at a rehabilitation centre, following the acute hospitalization phase. Mette is pregnant and is to give birth in a month to her first child. During this first meeting, Mette tells a story about being happy and relieved because she got off so lightly (with the ABI). She often finds it hard to look at the other clients at the rehabilitation centre who are more severely affected by ABI. She seems happy and cheerful, and smiles during the interview. Mette is being discharged later that day after only two weeks at the rehabilitation centre, since she has no physical consequences. She agrees with the centre's evaluation; she does not feel any severe consequences from the ABI. During the testing (for depression and quality of life) she mentions that a rehabilitation plan has been made, but Mette says, "There is very little help I need." The tests disclose a high quality of life, with no sign of depression. A nurse from the psychiatric ward (pediatric) will get in touch with Mette when she is about to give birth to her son. The doctor has arranged this to prevent any delayed psychological reactions during or following labour. However, Mette does not report any psychological consequences so far. On the contrary, she seems relieved, and during the testing she says, "Well, you won't find much here." In connection with the rehabilitation, she explains that she has never felt "lost" (in the sense of getting what she needed), and that she has received all the support she could wish for. In this excerpt we see a young woman, soon to	NOT >6/12

	be a mother, who positions herself as lucky that she has experienced hardly any consequences from the ABI. We see how Mette constructs herself as different from the other clients with an ABI (identity dilemma 2: sameness and difference) and how she got off lightly compared to them. In this way, Mette constructs herself as the same (constancy) before and after the ABI. She positions herself as the one who got off lightly and, therefore, hardly feels any change (identity dilemma 1: navigation between constancy and change). The first author also co-constructs Mette as happy and even cheerful in the accounts describing her.<br><br>The Second Meeting<br><br>The second meeting takes place about one year later. A lot has happened in Mette's life since the first meeting. She has become a mother and moved into a new house with her husband, Christian. Mette is still waiting for clarification in regard to her return to work. The interview begins with a question about how Mette experiences the ABI today:<br>I: Can you tell a bit more about how you experience the brain injury today?<br><br>M: It burns all the way up here (points at her arms), in the skin. It is resilient, it is sore and in all of my muscles there are some big muscular lumps […]. There is a constant tension in my leg, so it is also very swollen and sometimes I cannot really be anywhere because I simply feel pain in the entire arm and the entire leg.<br>I: No. Okay. Is there anything else?<br>M: Not related to the body.<br>In this first excerpt, Mette is focusing on the physical consequences. Later in the interview, Mette addresses the psychological aspects as well and says, "It is really, really big psychologically." Becoming a mother and having an ABI was especially very difficult:<br><br>I: So, which things come to mind, when you think about what it was like, becoming a mother?<br>M: Well, anxiety, bad conscience, bad conscience and bad conscience.<br>I: Yes, okay. How did you experience that?	Explains the physical effects of the stroke and the pain.<br><br>Challenges regarding the condition and being a mother.

<table>
<tr>
<td></td>
<td>M: (M is crying.) Well, I could not be there for my son, I could not get up at night and nurture him, I could not give him milk and... [...] And he would just lie there, the little guy, and be all perfect, but I just could not. I have not even been able to go swimming with him, so we have been home a lot. I could not think about meeting the maternity group, and it has been difficult for some (of the other mothers from this group) to understand, that I did not go out with them. So it has been really hard going from being very, very social to living in a box.

When Mette is asked what it was like to become a mother, she answers in terms of two specific terms: “anxiety” and “bad conscience,” of which “bad conscience” is repeated three times. By doing so, Mette constructs her position as a mother in a negative way. She continually uses the words “I could not,” thereby positioning herself as a passive and incapable mother and in opposition to her son, who is positioned as perfect and innocent. Meanwhile, it is interesting that Mette, at the beginning of the interview, points out the physical complications and not until later in the interview says that “I am not that affected physically. It is really, really big psychologically.” The depression test conducted at the second meeting indicates that Mette shows signs of severe depression. Furthermore, there is a significant decrease in her psychological and physical quality of life compared to the pre-testing one year earlier. From these excerpts, we see a young woman who, in comparison to the first meeting, now suffers from the ABI in a highly psychological way. Mette constructs herself as an inadequate mother, both in relation to her son (not being able to nurture him) and according to social norms (not being part of the maternity group). Thereby, Mette constructs herself as different from others (identity dilemma 2: sameness and difference), but at this second time, the difference from the first position is a negative one. Furthermore, she constructs the transition from being social to living in a box (identity dilemma 1: navigation between constancy and change) as hard. This construction points to the conclusion that Mette cannot cope with the changes and the navigation of these identity dilemmas. For this reason, we need to take a closer look at the rehabilitation process, in order to understand what might have negatively influenced the development of Mette’s sense of self.

A Physical Construction of ABI</td>
<td>Social implication of the condition and how exclusion social groups

Explains that the psychological effects of the condition are greater than the physical in her opinion</td>
</tr>
</table>

<table>
<tr>
<td></td>
<td>In the previous section, we saw how Mette explains the physical complications as the answer to how she feels about having a brain injury. Meanwhile, the following description shows that the psychological consequences affect Mette's life as well: I: Okay, is there anything in this short rehabilitation […] that has given you any new positive experiences?
M: No. […]. But it is also difficult, because you see, I am not affected that affected physically. It is really, really big psychologically.

I: But because you are not affected physically, that much-
M: Then they could not really help me.
I: Okay. So did you miss anything?
M: I do not know, you see, it was so hard having a brain injury, and you could not… people could not see it on me, and […]. It was so hard to be in a situation where you are ill but you could not…people could not see it, and I could not really see it, except that I was so tired.

In this excerpt, Mette positions other people as blind to the ABI: “people could not see it.” Furthermore, she continues and now includes herself in this position: “And I could not really see it.” To take on the position of not being able to see “it” could relate to the question of acceptance and how to accept something which is invisible. The hidden and invisible consequences of an ABI are then used as an argument for the conclusion she draws: “Then they [professionals] could not really help me.” Drawing on Bamberg’s theory of discourse perspectives (Bamberg, De Fina, & Schiffrin, 2011), the dominant discursive practices centre on the formation of a consensus of what is agreed upon and taken to be truth. When Mette refers to the fact that people, including herself, could not see it (the ABI), she draws on a master narrative saying that brain injury rehabilitation is mainly related to physical and visible consequences. In this way, Mette’s accounts of the physical consequences are a way of legitimizing the claim that something is wrong. Based on this analysis, we find that Mette constructs her ABI primarily at a physical level.

Positioning Processes: An Example of Self-Positioning and Implications for Identity</td>
<td>Impossible narrative as Rehab therapy will not help with her psychological condition being her priority

Disregarding therapy and dismissing its benefits.

Only recognising her condition due to</td>
</tr>
</table>

	Next, Mette talks about her previous job, where she worked as a healthcare service provider:<br>I worked as an assistant, also for older people […]. What does a person do, when he is hospitalized and what does a person do, when one cannot do the same things as before? It is actually a bit like what I am in right now. What do we do, to uphold the quality of life that we had before? Then we just have to compensate. (emphasis added)<br><br>In order to understand what happens here, we draw on the first two steps of positioning analysis: how characters are positioned within the story, and how the speaker/narrator positions herself (and is positioned) within the interactive situation (Bamberg & Georgakopoulou, 2008, p. 8). When Mette talks about her previous job, she makes an interesting shift from talking about her position ("what does a person do") as an "assistant" for elderly people, to suddenly including herself in this position as a patient ("what do we do?"; emphasis added). In this excerpt, we see how Mette shifts from positioning herself as a service provider, to suddenly being the one provided for. Still, it is interesting to see how she makes a kind of knowledge transfer when she uses her previous experiences as a professional in order to arrive at the conclusion about compensating in order to experience quality of life. Following Bamberg, the notion of small-d discourse can be used in order to interpret this quotation as a sign of wanting to be an agent, someone who takes initiative and actively constructs discourses and positions rather than being a passive recipient of those constructions. It shows a way of trying to progress and not regress, since Mette can use knowledge from a previous position to inform a current position. Therefore, the authors argue that this way of using knowledge contributes to and generates an identity process in which Mette becomes aware of her professional identity and can use it constructively in her recovery process. Meanwhile, the small-d discourse (agency) should be viewed as situated and not constant or universal. For instance, in the next excerpt, we see a shift from small-d discourse to capital-D discourse. This happens when Mette is asked about her future expectations:<br><br>I: What thoughts do you have about the future?	physical effects as these are the ones you see.<br><br>Recognising her previous identity and using that memory to formulate her new identity.

	M: It is definitely about an acceptance of what is going to happen with my life and a follow-up on that. Will I be a part of a vocational rehabilitation or what will happen to me, and what will happen to us?<br><br>Mette uses the phrases, "what is going to happen with my life," and "what will happen to me, and what will happen to us." In using these words, she accepts and actually takes on a more passive position of waiting and being in a standby position. Level two in the positioning analysis concerns how the narrator positions a sense of self/identity with regard to dominant discourses or master narratives. In the beginning, we saw how the dominant medical approach to rehabilitation constructed a physical focus. Moreover, we saw how Mette took on this position, and thereby positioned herself as helpless. Mette is positioned by the system as a passive recipient when she is waiting for a future clarification. However, Mette still needs to either accept or agentively deny this offered position: that is, she has to actively choose how she wants to reconstruct her narratives and identity in relation to this offered position.<br><br>The Question of "Was" or "Am" and the Ambivalence of Identity Reconstruction<br><br>So far, we have seen how Mette constructs her ABI, and how discourses play an important role in this process. Now, we take a closer look at how all of these aspects are brought into action and constructed as narratives. Next in the interview, Mette is telling us about the period of time when she met her husband, Christian. This was a time where she engaged extensively in social activities with her friends. Mette constructs herself in this period of time as follows: "I was the free Mette" (emphasis added) and continues:<br><br>M: You see, I am the social Mette, who has always been "the clown," who got totally wasted at the disco, right. […] And now, I cannot even consider having one drink. And sometimes my friends say to me, oh, they miss the old Mette. I: Do you miss her, Mette?<br><br>M: Yes, I miss her so much […] I do. I miss going out with my friends, going to a cafe and having a good time and all that. Because, I do have many friends. Well, just look at my Facebook	Is awaiting that she will not be the same as she is now in the future and accepting that change will come<br><br>Identifying condition as a burden or weighing her down.<br><br>Loss of previous identity and

	account, I got more than four hundred friends, right [...] but, it's not like I know them all that well, some of them are just acquaintances, but people I actually saw. Therefore, I think that they find it difficult to understand that I don't see them any longer.<br><br>According to the three identity dilemmas, Mette is here struggling with the dilemma of constancy and change. For instance, she says that she is the social Mette (constancy), in the temporal form of presence, then she elaborates on how she cannot go out and have drinks with her friends anymore and how they miss the "old Mette." Here, we identify a lost self: the social self. We find that the reconstruction process is characterized by much ambivalence. For instance, Mette says: "You see, it is a new Mette, that has come […]. Who is this new Mette, who is about to come, and will my friends accept the new identity, and will they still see me as who I was before?" Ambivalent accounts and different scenarios are presented here. Mette worries about whether her friends see her as she was "before" the ABI. At the same time, she wonders whether her friends will accept the "new" identity. Furthermore, she is actually presenting three different identities: a Mette that "has" come, a Mette who is "about" to come, and finally, the Mette she "was" before the ABI. These accounts show that Mette is navigating between different identities: who she was, who she is, and who she will become. When she asks, "Can my friends accept the new identity, and will they still see me as who I was before?" Mette also constructs a potential risk of losing her social and personal relations. Earlier, she constructed the loss of the old Mette, in saying that both she and her friends "miss the old Mette." Moreover, she says: "He [her husband] fell in love with a Mette who is not there anymore. And how does that affect his love for me?" Mette preferably wants to position herself in a way that would help her maintain her social and personal relations. The analysis shows that this is also why Mette cannot quite accept this developing identity (the "new Mette"), and therefore, she holds onto who she was (the "old Mette"). However, by constructing such a hope of going back, she also constructs a possibility of being the same and, thereby, being part of the same social environment that she once belonged to. In this case study we find that the construction of a lost self has to do with being the same, but different. Mette reckons that she is not the same; however, she does not know who she will be in the future. In sum, the analysis points to different factors influencing the loss of identity and the	understanding it will not be like that again<br><br>Loss of identity and relationship with partner changes<br><br>Denial and fear for the future

	identity reconstruction process. First, we saw how master narratives in rehabilitation practices enter into the process of identity reconstruction. Later, we found that Mette on one hand wishes to construct herself as she was before the ABI, but still has to reconstruct herself and accept this position as different. This is necessary in order to integrate the three presented identities (a Mette that was, that is, and that is to come) into a more coherent narrative.	
7. Green & King, (2009)	Being vulnerable , Vulnerability was the perception of participants that there was little they could do to control the events in their situation.<br>Lack of independence. Vulnerability due to the need to accommodate current functional limitations into daily life activities and feeling insecure about the ability to do so.<br>Discontinuity of normal life resulted in distress and anxiety. For men, these issues and concerns revolved around their masculine image.<br>Men felt their personal identity was strongly linked to their perceptions of their masculine self. That is, characteristics and elements of the masculine role such as physical strength, being the provider and protector of the family were threatened by the after-effects of the minor stroke. One participant said: "the stroke has made me useless. I cant do things for my family like protect them. If an intruder came in I couldn't even defend my family".<br>Men in this study measured their recovery by the extent of physical recovery attained, with clear goals and expectations set early in the recovery trajectory .<br>Emotional, that elicited reactions of anger, anxiety, depression, denial and ultimately adjustment. A 60-year-old man whose life revolved around his farm commented "I am very frustrated and disappointed in my lack of progress in recovery… I cant do the chores; I cant even hammer a nail.<br>Tension exists… regarding my lack of sexual performance. I started taking medication for anxiety attacks that started after the stroke. This has destroyed my sex drive.<br>The wife-carefivers engagement in hyper-vigilant monitoring of the health and well being of their husbands.<br>Coping strategy adopted by the wife-caregivers to alleviate the distress and anxiety engendered by the stroke event.	Pt having little control, passive agent<br>Reduced functional ADL physical limitation<br>Identity as a male<br>Role as the protector of the family<br><br>Physical ability used to identify recovery<br>Emotional/ denial<br>Related to ADL/ chores (meaningful ADL)<br>Effect on relationships<br><br>Hyper vigilant as a coping mechanism

	For many this role actually appeared to contribute to increase stress and anxiety as they became fatigued from being constantly on guard. One wife-caregiver remarked that she “sleeps poorly at night” because she worried about her husband can commented “…my husband would kill me if he knew that I checked on him in the night to see if he is still breathing.” Another wife remarked, “My husband accuses me of babying him. I have increased stress”.<br>Being careful not to be seen as critical of their abilities and contributions to the family during the recovery period. On wife-caregiver commented, “he is hypercritical of being evaluated and accuses me of judging him”<br>Realization. There was also awareness about the potential long-term impact of the minor stroke at an individual and couple level. Recognition and adaptation would be a continuous process, perseverance and resiliency would be necessary in ensuring the family would remain intact.<br>Over time, many of the participants became self-reflective, refocused on the meaning of family and marital relationships and began rethinking priorities in life<br>Loss. Referred to elements of their lives, physical emotional or social that had been lost or altered by the minor stroke event.<br>These losses in turn affected independence, autonomy and coloured their sense of self as previous activities and social contacts became limited. One participant remarked “it’s a struggle to accept the fact that im not the person I used to be, that I can’t contribute like I used to”<br>I am entering a transitional period, where I will have to learn to get over my past perceptions of self-image. Perhaps it is time to focus on other things.<br>A selfemployed farmer expressed his concerns about his ability to return to farming if his persistent difficulties with his walking/ hand and arm strength did not improve.<br>Many wife-caregivers also expressed a loss of independence and autonomy, as they restructured their daily routines to accommodate the presence of the husband.<br>“I find he stays home more, and this is stressful because it changes my routine.”<br>Sense of self. Gradually began to restore balance in their lives. This new balance was achieved by an appreciation and acceptance of what had transpired in their lives. Current reality	Reduced QOL for Wife caregivers due to fatigue<br><br>Being constantly on guard<br>Awareness of recovery being a long process<br><br>Rethinking priorities<br><br>Loss of identity<br><br>Loosing social contacts due to limitations<br><br>Accepting and recognising new identity and move on.<br>Returning to work functional/ identity<br>Spouse loss of identity.<br>Change of routine<br>Self persuaded, Acceptance of new reality<br>Adaptation

	One man remarked " I will not be getting any better" and noted that instead of looking for changes "I am learning to adapt! The emerging reality therefore was one of continuous change and adaptation.<br>I am in a transitional period… the arrogance that I had before, that I was fit and masculine and therefore immune to disease is gone now… previously I had dichotomy between my physical and emotional needs. The stroke has highlighted the need to develop all aspects of myself in order to have a better QOL. It has taught me the importance of moderation and balance.<br>I value my partner more and other relationships more. I have stopped worrying about the little things. The stroke put my life into perspective. Many couples felt the future as they had envisioned it immediate or long term was now different. For some, this was a difficult transition and they felt their future was in jeopardy. For others, this became a positive experience. they were able to focus on being together as a couple affording them the opportunity to "talk more openly", "share more" and this prompted a "greater appreciation of each otherand the roles we play in the family.	Recognising old self is no more<br><br>Everything into perspective = bringing relationship closer
8. Hersh, (2009)	Narratives in a biographical context<br>Marjorie expressed her understanding of ending therapy in terms of leaving the ''class'' and the learning environment provided by Elizabeth. She described herself as having been a ''perfect pupil'' and it seemed reasonable to her to withdraw from therapy because she felt that she could no longer fulfil her responsibilities to achieve or to learn. Her aphasia was something she had worked on as if she were back at school. She continued to seek pleasure in books despite her difficulties in reading and she told me that looking at books was important for her ongoing sense of recovery and quality of life.<br>Eddie -He was devastated that he no longer felt in control, that recovery took a long time, and that he could not resume his previous role as the quick-wit and raconteur, even though his physical and language impairments were, on the surface, relatively mild.<br><br>Notions of recovery<br>Michael, a 69-year-old farmer, described his prognosis as ''unbeknown''. Doreen reported that Wilfred's therapist had admitted that ''he may never talk … but then again, there is a chance it	Relaying on previous identity of being a teacher and relating that to her situation<br><br>Hobbies for QOL<br>Being able but not at a desires level of function to resume previous identity role.<br><br>Not knowing what the future holds for recovery.

	just could happen''. Ruth also remained convinced that David, who was severely aphasic, ''could do very well'' but did not think he could do that without more speech therapy. People tried to deal with uncertainty by obtaining advice or snippets of information from others, both professional and lay. For example, Claire told me she had lost belief in further recovery because she had been given advice by her local doctor that her language was unlikely to change. When asked how she felt when her speech therapy stopped, she said: ''I didn't care. I think the doctor said I wouldn't get any more [language].'' In contrast, Ruth's confidence that David could improve with speech therapy was influenced by hearing the successes of others, even if their circumstances were different: I do think he needs continued speech therapy. I have heard too many cases of people who had similar things to David and they do so well with a good speech therapist … I have a friend who had a little boy and they always said he was just like David, the aphasia part of it was so frustrating for him … and he came on tremendously … and I think David could do the same.<br>Similarly Neil, aphasic following a stroke 18 months before when he was 63 years old, compared himself to other members of his local Talkback Group (a local aphasia group; Hersh, 2007) and judged the time he needed for change according to the time it had taken others:<br>I'd like it to be better but I don't know whether … I … this Talkback is … he was one of my … he stroke about three years ago. I don't know whether my speech is going to get any better because I've been two years …<br>Ron, however, had reached a level that he could live with and did not believe he was going to change. Cheryl told me she had not expected to get better so was surprised and satisfied with the progress she had made. In the context of such individual responses to recovery, it is not surprising that discharge decisions will hold varying significance for people with aphasia. Judgements by clients about how much therapy is enough are difficult when there is such uncertainty about what might be achieved, what is possible. These stories show that people with aphasia get their information about potential recovery from many sources, not just their speech pathologist. In addition, their notions of recovery were not necessarily compartmentalised into professionally defined areas. Discussions about language change were merged with other influences on wellbeing; for example, Claire's changes in medication, Paul's confidence with swimming, or Wilfred's growing confidence in using his left hand. For Ron,	lack of clear information<br><br>relying on the knowing and advice of others<br><br>success stories of other instilling hope<br><br>Medication intervention aiding success<br><br>Hobby success stories

	cycling was just as important a response to stopping aphasia therapy as doing language work. When I asked him about his speech pathologist's unsuccessful efforts to secure community-based aphasia therapy for him, he remarked:<br>I've done most of the work by myself. I used to sit up in the doorway on the card table and do three hours exercise … I bought myself an exercise bike at a garage sale. I used to do 30 kilometres …<br>These examples reflect the importance for researchers of contextualising experiences and not making assumptions about the meaning people attach to events. The degree to which people felt able to alter their recovery chances also varied. Some assumed a sense of control in which they could manipulate recovery through effort or management of circumstance.<br>Ron's belief in exercise is a good example. Several people emphasised the importance of informal supports, particularly through family and friends, and through socialising. For others, the effort put into recovery was more akin to relearning, with an emphasis on a set of tasks, consciously chosen. Sharon, who was only 44 when she had her stroke, viewed recovery as dependent on solid work. She told me ''if you don't work on it, it's not going to happen for you''. Using her children's old books, she set herself tasks once home from hospital:<br>I got all their old kindy books … from when they were little … I couldn't even do the alphabet. Couldn't do anything … I'd sit there all day … I sat there and I just went through the alphabet every day.<br>Unlike Sharon, Matthew focused only on the tasks he was given in therapy, repeating the work he was given over and over. His wife, Phoebe, explained: ''It was a remarkable improvement but let me tell you, every waking minute he was working at it. There was absolute determination to do well.'' Some people had sought recovery by being model patients while in therapy, equating recovery with enthusiastically doing exactly what their rehabilitation professional asked of them. James had also invested a lot of effort in therapy and found it helpful to a degree. But by the time I spoke to him, over 2 years since his stroke, his frustration and anger existed alongside a resignation that he could no longer exert control over his recovery:<br>Shit happens. So what do you do? I mean let's be honest. I mean so you have to deal with what the card is dealt to you …	Physical changes being a catalyst for change and success<br><br>Network of support<br><br>Individual motivation and recognising if you work hard you succeed and help recovery.<br><br>frustrated by their slow rates of progress and lack of control.

	For others, therapy played less of a part in their recovery. For some, simply getting out of hospital or rehabilitation and being back at home made them feel better.<br><br>The importance of aphasia therapy<br>David and Ruth lived in a country town and travel to their local community health centre<br>Transport issues were also important for Marjorie who was picked up early in the day by a community bus and then spent 2 hours on it each way while it picked up other people or dropped them off. She was exhausted by the time she arrived at her appointment. Parr et al. (1997) also noted the frustration of many of their interviewees who were either unable to access therapy services or for whom the transport issues were so burdensome that they outweighed the benefits of having therapy. Another interviewee, Paula, became very stressed waiting for tardy taxi drivers and resentful at the costs incurred. Paula told me that she ‘‘felt safe’’ seeing her therapist at home but refused to continue therapy for more than a couple of weeks when she was asked to have sessions as an outpatient. She was 61 years old and felt that she was too young to be seen somewhere for therapy which was associated with more elderly and disabled people. As with this last example, the setting of therapy was important. For many, the period of residential rehabilitation, being away from home and in a regimented and intense environment in close contact with other patients, was highly significant in their narratives. For some this was comforting and reassuring, a chance to plough their energies into the tasks of rehabilitation. Michael and David had such positive experiences during residential rehabilitation that further aphasia therapy once back in the community could not live up to it in their eyes and was not considered as useful. For others, residential rehabilitation was a disturbing removal from their homes and a time to be endured. The benefits of therapy became secondary to the urgency of getting home. In previous studies where clients have been asked about therapy, they have often expressed a desire for more intensity or frequency than they received (Horton et al., 1998; Parr et al., 1997). In the present study there was variation in people’s satisfaction with the timing and frequency of therapy. People pointed out the contrast between the intensity of work in residential rehabilitation units and the long gaps between sessions in the community, particularly for those in rural areas. Claire received five sessions over 4 months. William had visits every 2 to 3 weeks for the first 4 months and then	Transport to rehabilitation session becomes a barrier to success<br><br>Being proud and trying to keep old identity and assuming social stigma causing a barrier to rehabilitation

	once every 2 months. Sometimes there were long gaps without services between discharge from rehabilitation and community-based therapy. Several people would have liked intensive therapy later on in their recovery rather than in the first few months. Others would have liked the flexibility to dip in and out of therapy depending on their health and energy. If less therapy was received than people wanted, the uncertainty of their future was compounded by questions about how much better things might have been, had they had more access to therapy. Discussions about therapy always included equal mention of the therapists themselves, their personality, style, and therapeutic relationship. Comments were mainly, though not always, positive, and for some the relationship became quite close, as Phoebe, whose husband Matthew received domiciliary therapy, told me:<br>The happiest possible experience for me was to have Hazel as not only a professional helping Matthew but it was like a sister and a mother and a very good friend propping me up as well. She was all encompassing as far as I was concerned. She was wonderful.<br><br>CLIENTS' VIEWS OF DISCHARGE FROM THERAPY<br>Interviewees often described individual activities but without demonstrating much understanding of the reason for those particular activities or how they fitted within broader therapeutic aims:<br>She had names … like opening a window or simple things, but things I couldn't do, you know? And you had to work out the numbers from it and all that and she kept on at that. And that is what happened all the time. (Harry)<br>… she just talked to me and I talked to her and I don't know, that's about all there was to it. (Cheryl)<br>Despite this, many people were positive about therapy and found it worthwhile, devoting a great deal of effort to it. For some, the exercises themselves were perceived as the main reason for their improvement. For others, it was the encouragement that was most important. Although Matthew could only speculate about the role of his therapy in promoting change, he remained adamant that he had needed it:<br>I can look back at a distance and say ''what was important about the set therapy?'' Would I have improved as much without therapy? I choose to think I wouldn't have but you don't know	Benefits of Therapy intervention not only for recovery but also social support role within the family.<br><br>Positives of therapy intervention being the reason for success

	… You don't know if it's a normal process … I don't know what part the speech therapy played … I wouldn't have been without it. It would be too risky to be without it.<br>A few people were less positive, complaining about therapy being too theoretical or irrelevant to their particular needs. Claire found her therapy patronising and ''stupid'' as her husband Tom explained:<br>Well, I think it was a lost cause. She (the therapist) had a routine … with the cards and all that sort of gear. To my mind that's a bit degrading, putting you back to about grade one or two at school, you know, hat and cat and mat stuff … that would frustrate me …<br>Claire's comments about her therapy tie in with her earlier comment about not being too bothered when it finished. Her negative feelings about therapy may also have reinforced her acceptance of her doctor's comment that she was unlikely to improve further. This section has explored three influences on people's narratives about discharge: biography, notions of recovery, and views on therapy itself. The contexts in which people express their views are very important and perceptions about the value of therapy and the significance of it coming to an end are closely woven into other aspects of their lives. In the next section I look in more detail at their perceptions of discharge itself.<br><br>Sometimes clients perceived that clinicians touched on discharge briefly but they were not prepared to air every aspect of the issue. For example, Eddie felt unable to ask Ros for more therapy because of his perception that she was so busy. Similarly, Bill had a ''bit of a chat about it'' but he was not completely open:<br>Bill: In the back of my mind, I sort of thought, ''well, if it did continue there is a possibility that things could get better''. It is one of those things that you don't know and with it not being there you are never going to know anyway. DH: Did you tell your therapist that? Bill: No. No, I didn't want to make her feel obligated or embarrass her or anything like that. No, I wouldn't have done that.<br><br>Collaboration in decision making<br>Only four interviewees reported that decision making for their most recent discharge had been mutually agreed or shared in some way. Their stories of discharge involved a sharing of	Loss of motivation to adhere to therapy due to feeling degrade

	information, deliberation in the context of real choices, and some degree of agreement on the final outcome. Clients had sufficient understanding of therapy itself and of their condition to make a judgement about the value of continuing therapy. The therapeutic relationships were strong but clients had other resources, sources of support, and were able to regain a sense of continuity. Shared decision making and mutual understanding did not necessarily mean complete satisfaction. For example, James agreed that his therapy should stop—both he and his therapist felt that it was no longer achieving a great deal—but he was far from happy about his residual disabilities, difficulties returning to work, and family disruption. Interestingly, all four of these people had good communication skills, a finding with important implications for how best to involve people with more severe aphasia in decision making. However, I am not suggesting that people with mild aphasia are always necessarily better able to influence decisions than those with severe aphasia. Eddie and Harry were examples of people with sufficient language to negotiate the issue but both felt unable to be involved. Paul, David, and John all had strong advocates in their wives but were still unable to influence their discharge decisions. For example, Ruth did not believe that there was any real sharing: ''No, they don't negotiate it. I think they tell you what they'll do and you agree with it … See, I just agreed with everything.'' In six other cases, clients reported that they had made the decision to finish therapy (including Doreen's account of Wilfred refusing ongoing help after leaving rehabilitation). The other 12 suggested that the discharge decisions were made by the speech pathologists(or in the cases of Cheryl, Ron, and Paul, made by healthcare teams). Ron said: ''I was happy to get out of the place. But I didn't have a say in the discharge date.''<br><br>Follow-up<br>Offers of follow-up services were clearly available for some clients. Doreen knew that domiciliary speech pathology services were available as soon as Wilfred felt able to accept more help. Equally, Marjorie and Sharon had both been offered more therapy if they wanted it in the future. Matthew and Phoebe believed that their speech pathologist would always remain willing to help if they needed her. Phoebe told me: ''I still do not feel that we are abandoned. I still feel if I needed to, I could ring her at the drop of a hat and she would come back to us.'' Other people felt able to request further services but only with changed	Lack of control of their own fate.<br><br>Having confidence in the service for support as a safety net

	circumstances. Bill told me of Edna's discharge: ''I got the feeling at the time when it happened that it would probably be final.'' But loss of therapy prompted Bill to consider alternative strategies for his wife: ''I realised … that we were not going to get anywhere and that's when I decided that I have to look at other options. And this is when I approached the doctor regarding this medication.'' He maintained that changing his wife's drugs had improved her level of functioning, and on the basis of his renewed confidence he was planning to contact the therapist again to ask for more help ''even if it is only in the short term, just to satisfy my curiosity''. In contrast, others felt there were no options for follow-up. Yvonne thought that if she rang to request more therapy for John then she would be refused and she was not prepared to accept group work as an alternative:<br>I just feel she would say ''no''. Because I have asked her once and she said take him along to those sessions … the group sessions. He hates that. He doesn't join in or do anything and it doesn't do him any good at all.<br>Similarly, Eddie did not even consider asking for more individual therapy even though he would have liked that. He said: ''I didn't feel like doing that because I know how busy they were … they got a queue a mile long trying to go in …'' Like John, Eddie was offered group therapy after he had finished his individual sessions and he also decided not to continue with the group. But when he stopped going, after a week or two, nobody rang him to see where he was or whether he was all right. He thought it might have encouraged him to go again if he had been contacted. Anna would have liked James to go to a group, but the nature of his language deficits meant he could not benefit from this option. She stressed the importance of ''the after therapy support'' and continued individual counselling sessions with the speech pathologist even after James finished therapy. Finally, continued contact with speech pathologists after finishing therapy was important for a few clients, even if it did not entail any language work. William was thrilled when Tina contacted him for inclusion in this study. Marjorie loved seeing Elizabeth when she visited her work for other reasons. Keith and Ron both made a special effort to visit their previous therapists. Harry also wanted to stay in touch with his therapist, Helena: ''And I'll say this for Helena too that she does phone up to see how I'm going sometimes … I told her. I said 'come and have a cup of tea if you want to'.''	Being abandoned by the therapy service loss of hope and opportunity for recovery

	Feelings about discharge<br>The way that interviewees felt about their discharge varied according to their perceived degree of recovery, how valuable therapy was to them at that point, and the degree to which they felt able to be involved in the discharge process. Those who spoke most positively about their discharge had generally recovered well and had participated in the discharge decision. For example, at the time of discharge, Sharon felt she could resume most of the activities she wanted to do. She no longer viewed therapy as necessary and she felt able to discuss this with her speech pathologist. On the other hand, Bill did not know whether Edna was able to make more progress and he would have welcomed continued therapy: ''We would have preferred that she still kept coming irrespective of how we both assessed the situation'' (Bill). However, he and Edna did not feel that they could challenge their therapist and assumed that her decision was inevitable: ''I accepted the situation as it was because I couldn't see any other way forward.'' Bill and Edna's situation was similar to that of Harry and Barbara. Harry told me that because he loved reading, he should have been viewed as an enthusiastic candidate and offered more therapy. However, the couple firmly believed that the decision was financially based, beyond their therapist's control, as Barbara told me: ''We thought it was all he was entitled to.'' Looking back on their experiences, interviewees also recognised that their feelings about discharge were influenced by other aspects of their lives at the time. Matthew and Phoebe thought that discharge from aphasia therapy occurred at the right time for them because, by coincidence, they became very busy with decorating and moving house:<br>I think if the house painter hadn't come into our life at that precise second, our life would have been different. With the painting of the house, we've been moving furniture and changing rooms and doing things. We would have continued if he'd have not come. So that was therapy of a different sort.<br>Similarly, at the time when Keith left individual therapy, not only did he continue with the Talkback Group, but he also became ''hooked'' on his newly acquired computer that appeared to replace his need for further therapy. Neil ploughed his energy into his gardening and Ron into his carpentry. Discharge from therapy was particularly significant for those people who felt it left a gap or who felt disturbed by the uncertainty of knowing if they could have improved further. Paul and Carol felt let down, disappointed, and angry, not at their therapists	Possibilities to achieve activities clients desire to achieve due to therapy intervention<br><br>Reliance on therapy being the reason for success, without it, there is not hope.

	but at the health system more generally.Finally, several people were saddened at stopping therapy because they missed the company of their therapist. This was certainly the case for Marjorie, Harry, William, and Wilfred. However, they balanced this by recognising that their therapists were busy with responsibilities elsewhere, and that their relationships were always going to be transient and for a particular purpose.	
9. Kitzmüller, Asplund and Häggström, (2012)	The Family as a Lifebuoy<br>Supportive family relationships were seen essential for stroke survivors' survival and recovery. During the acute stage, it was important for spouses to ensure that stroke survivors received optimal treatment and care. Such involvement could result in transfer to more specialized hospitals or rehabilitation units. Later, spouses tried to look for new treatments or remedies to improve the stroke survivors' functional abilities. Often, they succeeded despite the stroke survivors' poor prognosis.<br>I would not have managed without my wife. She has pushed me all the time, Without her, I would have stayed in the wheelchair and lived in a nursing home [Harald, stroke survivor].<br>Both stroke survivors and their spouses wanted to be reunified in their own homes as soon as possible after the stroke. Family relationships were essential for boosting the survivors' will to live; for example, being a mother of a small child could restore a will to live despite severe poststroke depression. The family's encouragement and emotional support helped stroke survivors to carry on.<br>It meant a lot to me that I had a big family as long as you have a family, they will always stand up for you and you must keep going [Eva, stroke survivor].<br>Encouraging spouses helped their ill spouses achieve self-confidence, motivation, and independence. Stroke survivors expressed gratitude for having been pushed forward by their families. The dreams that stroke survivors recalled from comatose periods can be seen as metaphors for how family members are perceived as rescuers.<br>I perceived that my son was my rescuer in the helicopter; he saved me several times was meant to be together with this little family of mine [Henny, stroke survivor].<br>Spouses also provided tremendous practical support during both the acute stage and the subsequent years after the stroke. They had to act as nurses and physical therapists while fighting for their disabled spouses' rights and taking over roles and responsibilities in a	Recognising that the sole reason the patient has progress is due to what their partner has done.<br><br>Patient appreciation that family motivate and encouragement

	supportive, yet determined manner. Spouses were convinced that their actions improved the rehabilitation process but emphasized that they, too, needed emotional and practical support. Support from members of the extended family was crucial, as spouses commonly experienced poor help from nurses or other healthcare workers. Practical and emotional support allowed spouses to maintain a work life and to keep up with their caregiving obligations during the acute stage.<br>We got a lot of help from my wife's sister. She moved in and lived with us during this time. Her help meant a lot [Torgrim, spouse].<br>Absent Presence Both the stroke survivor and the healthy parent had to be away from home during the acute and sometimes critical stage and, later, had to concentrate on a demanding rehabilitation process. Despite major changes in the family members' daily lives and the difficulties of fulfilling their parenting obligations, couples reported that their children had not experienced much harm, as they had received good care from the healthy parent and members of the extended family. Parents explained that they had been most concerned about their own situation and did not remember their children's reactions during the acute stage. They mentioned that their adolescents had left home early and moved far away. With one exception, parents did not see a connection between their children's early departure and the stroke event.	
	This is not only about me; it [the stroke] was destructive for my children My daughter moved to another county when she was only 16. I think she wanted to be far away think it was best for her [Rita, stroke survivor].<br>Single mothers felt that they had abandoned their children when they were left to the care of extended family members for the first time after the stroke. The mothers attempted to compensate for such absences with exaggerated efforts to be especially good parents after the stroke. Behavioral problems, difficulties with concentration, and learning deficits among their children were noticed by these single mothers.	Outcome: the effect of the stroke affected parent- child relationship with distance being created
	My son Is a little spoiled, but my only job now is to be a good mother and that has to be done well of course I felt that I had abandoned him [he was 8 years old then] he needed much support but you cannot choose these thing therefore I wish to do especially much for him now	Trying to maintain role as a "good mother" by spoiling child as a sense of purpose

	.I have the feeling that I have been away from his life when he needed me [Harriet, stroke survivor].<br>All stroke survivors with minor children at onset were women. They talked about short comings in their parenting as they could not meet their children's needs. Their husbands had to take on parenting alone, and this change was difficult because they were not familiar with these new roles. These stroke survivors experienced mental pain, self-reproach, and sadness when they suddenly realized that their children had turned away from them.<br>I cannot describe how it felt it was a shame, a horrible shame became even more depressed [Line, stroke survivor, about her child turning away from her].<br>The sudden and shocking onset of the stroke created a fear in the children that the parent would die or that something bad could happen to them too. The children also experience do ther frightening situations, such as being alone with a parent with epileptic seizures or finding a parent after a suicide attempt. According to the parents, the children had shown psychosomatic reactions, but parents did not connect these reactions to the stroke event. He [8 years old at onset] got troubled with his stomach, he had much pain and we visited the doctor and it kept going. At the end, we were sent to the hospital they did not find anything [Torgrim, father and spouse].<br>The narrative of a participating child revealed feelings of loneliness, loss of freedom, lack of support, and the burdensome responsibilities of caring for a household and a minor sibling. The struggle to fulfill the sometimes self-imposed duties and being unable to do so caused the child to lose self-confidence. To keep the family together, especially when there were conflicts between the parents, the child tried to help the ill parent to relieve the healthy parent. Strong emotions were displayed during the interviews with adult children.<br>I felt that my mother and my father were absent [cries] I asked my little sister to sleep in my bed and that lasted for a long time; I wanted to have someone in my room together with me [Liv, minor at onset].<br>I remember very well my mother not being there and my sister taking the responsibility; it was a lot of responsibility she took me to kindergarten on her bicycle [Elin, minor at onset].<br>The parents and one of the children reported health problems during adolescence that disappeared after they left home. The illness was seen as a possible consequence of the	Failing as a parent

	childhood experience. Tense relationships between parents and children were noted especially when the child needed to take on burdensome duties. One child explained her decision to leave home early as a means to escape from responsibilities.<br>I only wanted to get away from home. I wanted to be alone. I moved out immediately after I finished school. I was tired of all the responsibilities I stayed away for eight years and I thought I would never move back to my home city. In this time, I felt that I had a very bad relationship with my parents now it is different [Liv, minor at onset].<br>Over whelming responsibilities early in life seemed to result in a preference for managing things alone or for performing perfectly. Parents did not make a point of these early responsibilities but admitted that there had been little talk about the illness within the family. Children observed that their parents were reluctant to talk, and so they kept silent. Nevertheless, the experiences sometimes had positive outcomes for the child, such as a greater awareness of the importance of health and improved coping strategies. A closer relationship with the ill parent was reported by the child when there was no need to take on caring duties. Couples could not answer the question about how the stroke had affected their children's lives, but they did reveal that they had noticed their children's reluctance to talk about the ill parent with others. Left with no professional support, one child talked about her tremendous need to find someone to share the experience with. Finally, she called volunteer telephone services.<br>We weren't seen had a need for someone to talk to, get information about the things that had happened to us, somebody who could visit us and talk to the whole family, how we could handle problems, whom we could contact, family counseling, maybe [Liv, minor at onset].<br>According to parents who had adult children living away from home, these children had difficulties accepting that the ill parent was not the same as before the stroke. They seemed to deny the situation, especially when they had not been engaged in all of the daily struggles and when they expected things to be as usual when they came home. Healthy parents missed their children's support and felt disappointed and abandoned. Nevertheless, they mostly excused their children for having lives of their own.<br>The kids are not realistic, they deny the situation. I have to tell them: your father is not the same as before [Gitte, spouse].	

	Aphasia deepened the gap between children and their disabled parent and made it difficult to maintain contact with children who lived in other parts of the country. Despite not being able to say a word, some parents called their children frequently to hear their voices and their news. Parents with severe aphasia felt disappointed when their children reacted with anger or withdrawal. Some parents realized that aphasia was stigmatizing for their children, and they failed to obtain professional support for the children.<br>It is very hard for the children in the family suddenly their stepfather turned crazy they should do something for them tool information, counseling [Gunnar, stroke survivor].<br>Broken Foundations<br>The couples were not prepared to face the changes in family life after stroke. Healthcare workers had not made efforts to assist in that matter. Stroke survivors could no longer fulfil their roles or meet their spouses' expectations. Role changes and altered relationships made the healthy spouses face responsibilities with which they were unfamiliar with. These role changes often caused fear and insecurity or interfered with work obligations. Spouses had to take sick leaves, reduce work time, or give up work altogether, and these changes reduced the familys total income.<br>I had a husband who managed everything before and all at once he did not so I had to take on the responsibility for our finances [Siv, spouse].<br>If it had not been for our little son, it would have been easier. It felt like an overwhelming responsibility just to find out how to dress him it was my wife who had the main responsibility for him and suddenly she suffered a stroke [Torgrim, spouse].<br>Living with a spouse with cognitive impairment and an altered personality influenced marital relationships. Sometimes, the spouse perceived the disabled stroke survivor as a stranger. Childish, aggressive, controlling, and jealous behavior endangered the mutuality in marital relationships.<br>Something has been damaged with the stroke; he doesnt understand as before and he cannot do things as he did he doesnt see his own situation like it is the worst was that his personality was changed. He should not get angry over trifles and let others suffer. There have been many conflicts [Birgit, spouse].	Feeling un equipped to manage family relationships impact on emotional support<br><br>Spouses taking on new roles and responsibilities<br><br>Lack of understanding and ability to cop with cognitive behavioural changes

	Some male stroke survivors felt that their spouses threatened to abandon them if they did not change their behavior. They admitted that they had not been easy to live with because they became bad-tempered or disengaged after the stroke. Disagreement also arose between couples about important decisions that had to be made.<br>I took over running the farm on my own today I cannot understand what I was doing. He understood his situation and wanted to selll I wanted to build up [Gitte, spouse].<br>None of these issues were discussed during the couple interviews, but they were disclosed in subsequent individual interviews afterwards, during which all participants showed more emotions through various types of body language. Living with a depressive spouse was trying. Healthy spouses had to push and pull as well as seek treatment. Serious and long-lasting depression with suicidal behavior in stroke survivors was an additional burden that affected healthy spouses' mental health.<br>It was bad enough when she suffered the stroke, but when she tried to commit suicide i felt like I had built a house and someone had snatched the foundation. [Torgrim, spouse].<br>Couples considered aphasia to be the worst disability after a stroke and regretted that aphasia got so little attention in health care services. Not being able to communicate caused grief and anger. All contacts with the outside world had to be made by healthy spouses who also had to defend their aphasic spouses' rights. Some spouses felt that their aphasic partners were over looked or were stigmatized by other people. They tried to educate others about aphasia and encouraged them to talk to their spouses. Aphasia was also considered to be a threat to stroke survivors' intelligence as they were no longer able to share their thoughts.<br>The worst thing is that I cannot have a conversation and discuss things with him. it is difficult so we turn on the TV. Aphasia is the worst thing! His intelligence is not as before; it is in his head but it doesn't show, and if it does it comes in a strange way; Im sure he has lots of thoughts, but we cannot take part in it, that's how it is [Siv, spouse].<br>Stroke survivors with severe aphasia had to rely on their spouses as interpreters, although their interpretations were not always correct. It was obvious during the interviews with couples that aphasic stroke survivors wanted to talk on their own behalf but had difficulties entering the conversation. Sighs and groans were uttered when the spouse dominated the	Barrier – communication between spouses resulting in loss of emotional connection

	conversation. Spouses of stroke survivors who had completely lost their speech abilities were forced to make guesses that caused misunderstandings and conflicts.<br>There is a lot of guessing but I cannot just fake that I understand the man I share 24 hours a day with, I can't There are lots of misunderstandings He is speechless but no one can imagine how animated our fights are [Mathilde, spouse].<br>Aphasia interfered with couples' social activities too. Friends and acquaintances disappeared, and the aphasic spouses withdrew from social life. Thus, the nuclear family lost important connections with its social network. This change made spouses struggle even harder to maintain a network by trying to compensate for their aphasic spouses' withdrawal. He cannot start a conversation; sometimes, when we have visitors, he leaves the room He refuses to visit our friends So it is he who withdraws, not them [Charlotte, spouse].<br>Spouses made efforts to increase their disabled partner's independence. Male spouses encouraged their partners to participate in demanding activities. Some female spouses were overprotective and limited their husband's activities when they considered the activity to be dangerous. All activities in the couples' lives had to be planned and prepared for and did not allow for spontaneity, a missing feature that was emphasized as a considerable loss.<br>Spontaneity is gone you cannot make spontaneous decisions anymore because everything has to be planned and arranged carefully I miss it a lot, Life has become a routine [Renate, spouse].<br>With few exceptions, caregiving around the clock was exhausting. For the spouses, it was difficult to attend to their own interests, as many of their partners were dependent on them. Stroke survivors wished to be a part of the spouses' life, a wish that at times was overwhelming and forced the spouse to seek an escape.<br>You are not a caregiver sometimes but your whole life becomes caregiving. If I stay in another room for too long he calls on meI Sometimes I have to leave the house. I cannot manage to stay there [Matilde, spouse].<br>Stroke survivors who continued to work found that their job claimed all of their energy, and they became too exhausted to fulfill their spouses_ expectations. Stroke survivors were aware that the situation was burdensome for their spouses and that it impacted their freedom and sometimes even their health. To relieve their spouses burdens, the stroke survivors gave up leisure activities or rehabilitation efforts that demanded their spouses' help. Feelings of guilt	Social isolation as a coping mechanism, embarrassment within social circles<br><br>Loss of spontaneity, coping by making routine and planning

	and a troubled conscience were common among stroke survivors. They felt desperate about being unable to contribute to the family's well-being because of the overwhelming role changes they faced, including ending their work life, not being able to do housework and impaired parenting.<br>He was forced to stay at home with me. He could not travel and attend seminars He could not even go out skiing, I had a very bad conscience because I could do so little [Henny, stroke survivor]. Stroke survivors struggled for independence in their marriage. Managing things alone became important. At the same time, it was essential for them to get the help they asked for. Being dependent upon their spouse caused irritation and anger and left the stroke survivors with diminished self-confidence.<br>Maybe I'm so irritated because of all the things I cannot do and so it causes someone to suffer [Karl, stroke survivor talking about his wife].<br>Struggles against dependency often led to considerable changes in the family's way of life. For two male stroke survivors, independence was of such importance that they decided to live on their own, although they did not separate completely from their wives. The decision was also made to save their wives from obligations. The partners continued to visit each other, share meals, and spend holidays together.<br>I told my wife: im going to move to another county and try to manage on my own give me two years[I I supposed if I had chosen to live with her, I would have made her do things for me now living on my own I cannot depend on her [Gunnar, stroke survivor].<br>Short-term relationships (less than 5 years) fell apart within the first year after the stroke. None of these participants had been married. Disability after stroke was seen as the main cause of separation as these stroke survivors did not want to limit their partners' lives. At times, the healthy partner could not accept the disabilities and role changes. Living with an unsupportive and criticizing partner caused disappointment and grief. Being left alone when the partner's help was most needed worsened the suffering.<br>I felt abandoned. He visited me only once in the hospital. I felt that he had let me down [cries] [Gerd, stroke survivor].<br>Also spouses felt they had been left alone grieving without healthcare workers' support. They reported unmet needs in being informed about how to deal with their ill partner's condition.	Partner being someone to express emotions towards, negatively<br><br>Reclaiming independence by moving out of the home from partner to undertake ADLS's independently

	Finding a New Marital Path Couples who decided to continue their lives together had to find a common path into the future. Together, they had to rearrange their lives and adjust to the changes that had broken their foundation. Most couples had been married for many years before the onset of stroke (Table 1). They knew each other well and were able to meet each others' needs. The relationship was strengthened as they grieved the losses and searched for information together. Exercising, as part of the rehabilitation process, became the most important and time-consuming activity in the couples' lives. Both stroke survivors and spouses had engaged in these activities with enthusiasm. As most couples continuously experienced progress, they continued exercising for a long time. After the stroke, couples spent more time together as working hours were reduced or terminated. Time became available for new activities. Couples who managed to find new leisure activities gained new acquaintances and friends whose support made other, more demanding activities possible. Outgoing couples kept more of their important networks than did less outgoing couples. Visiting peer groups was a prioritized activity. Learning about the illness and its consequences and gaining support from people with similar experiences helped these couples adapt to their new life situation. To maintain communication, couples dealing with aphasia developed new communication strategies using body language, facial expressions and demonstrative actions.<br>The other day he looked to the other side of the beach and laughed. I told him: B know what you are thinking about. I can read your mind. we have been married 51 years you know [Charlotte, spouse of a stroke survivor with severe aphasia].<br>After sometime, the changes in marital life became routine. Couples did not reflect much on the past; rather, they wanted to look forward. Love and attachment gave them strength to continue their lives together. Their strategy was to focus on opportunities rather than on limitations. Humor often seemed to be used as a means to deal with the difficulties. Most couples were aware of the necessary alterations after the stroke. Stories about other stroke couples' divorces from the peer groups were reflected upon and made them work even harder on their own relationships. With few exceptions, the participants expressed that they had a good life and found that their marital life improved, despite the strain. Being able to communicate openly strengthened the relationship. The duration and quality of the marriage	Recognising the issues face on relationship and embracing them together, and grieving together brought relationship closer<br><br>Shared difficulties with support groups with people and their coping mechanisms shared.<br><br>Being able to look forward and not at the past establishing a new routine.<br><br>Humour as a coping mechanism

	seemed to be an important factor in how the couples managed. Being able to solve problems together was seen as a shared victory.<br>We stay closer to each other. Now we talk more about our problems, we are more open towards each other. We have stuck together all these 30 years [Eva, stroke survivor].	Stroke brought family closer together emotionally
10. Morris et al., (2014)	Desired outcomes: preventative, restorative and participatory<br>We identified preventative, restorative and participatory outcomes. These were shared by participants, but differed qualitatively in interpretation. Survivors, carers and physiotherapists believed PA prevented future health problems including further stroke; improved survivors' physical selves, supported participation in valued activities and life roles and thereby contributed to adjusting, maintaining or restoring survivors' physical and social sense of self after stroke.<br>Preventative outcomes<br>All groups recognised that PA supported recovery, prevention of future stroke and other health problems. Some survivors with families were explicit about PA preventing death and promoting longevity. These beliefs were associated with fear of future health events, life disruption that might arise and resultant family burdens. We conceptualised these outcomes as ''preventative outcomes'' that reflected desire to face forward to future life. As one mildly disabled stroke survivor, who had a stroke 16 years previously and participated tai chi and swimming in said:<br>After a stroke, well, I'll tell you, it gives you a right rude awakening after having a stroke, because you think ''Well, I could have been away, how am I going to improve my life, really?'' It frightened the life out of me....''Oh, hey, come on. Shake up and do something, improve what you did before, improve your fitness, improve your lifestyle'' [Female survivor, 69 years old]<br>Many carers feared future strokes. Their fear was magnified by physical effects of stroke and other conditions, suggesting exercising to preventative levels was difficult. One carer, who was retired and whose husband had a stroke two years previously expressed this:<br>I was a bit scared because I realised if you have one, there's a possibility to have more...I nag him but he also has Parkinson's and is less mobile which worries me [Female Carer, 62 years old]	Feeling of being awakened by the stroke, establishing a new life path and having motivation to achieve.

	However, physiotherapists tended to take a retrospective approach in which PA was orientated towards preventing deterioration in physical gains made in rehabilitation. As one physiotherapist who had worked in a stroke unit for eight years said:<br>It will maintain them at that level if not improve them. I think without doing anything at home you do get a number of patients on discharge they've worked hard in hospital, but they will go home, they will sit and do nothing but they do decline frighteningly quickly......[Physiotherapist, acute/in-patient rehabilitation, qualified 13 years]<br>Restorative outcomes<br>Many survivors described PA as a mediating influence, enabling them to replace the acquired ''disabled'' status with a ''normal'', independent social sense of being in line with their pre-stroke selves. PA could address disruption caused by stroke and restore how and who survivors were before their stroke. We conceptualised these as ''restorative'' outcomes that were backward facing towards pre-stoke identity. One survivor with low disability who had a stroke two years previously and who participated in a stroke exercise class described restorative outcomes thus:<br>Yes, I really feel it [exercise] helps me and its an accomplishment that you're doing something you know.... because I don't want to be regarded as a disabled person, I think it's just basically you want to be what you were before and its an ambition [Female survivor, 75 years old]<br>In line with survivors, many carers believed PA mediated restoration of survivors' physical selves that supported adjustment to stroke and participation in enjoyable activities together. In this way it supported their partnership. One retired carer whose husband had a disabling stroke five years previously explained how this occurred:<br>Well, in our case, A could really just walk and we went out every day and it was finding places to walk.... anything to keep walking and keep mobile and do as much as he could...in the end we travelled all over [Female Carer, 80 years old]<br>However, older more physically impaired survivors perceived restorative outcomes as less achievable and too effortful. These survivors accepted their situation, perceived limited possibilities for physical restoration and were less inclined to prioritise PA in their lives. As a retired survivor who had a stroke 10 months previously, and who only managed to walk short distances described:	Restoration narrative, recognising that exercise will help construed some old identity.<br><br>Exercises as a way of maintain a level of ability

	I just don't have energy; I just don't seem to have any. I don't have the inclination either you know. Aye, yes, I am fine lying in my bed the plans I have and what I am going to do and what I'm not going to do and it never materialises. [Male Survivor, 83 years old]<br>Physiotherapists often focused on physical deficits and function. For them, restoration often stemmed from their clinical practice within a ''deficit reduction model'' de-contextualized from survivors' and carers' lives. One physiotherapist who worked in an out-patient setting with young adults described her thinking:<br>I also think if they're getting stronger it is going to impact on their movement and balance at a really basic level.... if they continue to exercise at that level, hopefully their balance will improve because they're getting stronger.... [Physiotherapist, out-patient rehabilitation, qualified 26 years]<br>Physiotherapists' focus on physical outcomes led some community physiotherapists, to judge survivors' broader restorative outcomes and life goals as unrealistic. Uncertainty about possible recovery underpinned these judgments. Other therapists, who worked in community settings, were uncomfortable with judging survivors' goals as unrealistic, blaming themselves for not being honest.<br>It's about goals; sometimes patients' goals are unrealistic. [Physiotherapist, Out-patient, qualified 16 years] I don't think that's patients being unrealistic I think that's us not being honest or...[Physiotherapist Community Rehabilitation, qualified 11 years] We don't have enough information to say. [Physiotherapist Community Rehabilitation, qualified 25 years] A patient will set a goal in hospital and it may be ''I'd like to get independently to the hairdresser'', but they haven't actually been independently to the hairdresser for ten years. That does happen, that is really difficult. [Physiotherapist, Community Rehabilitation, qualified 27 years].<br>Participatory outcomes<br>For survivors, PA eased boredom and supported purposeful lives. Outcomes stemmed from enjoyable activities that ''mattered'' in life. These were conceptualised as participatory outcomes, were forward-looking and related to life goals post-stroke. Activities included hobbies, skilled household tasks, work and family roles, and social participation. A young	Understanding limitation and accepting these – restoration<br><br>Goal setting realistic achievable goals

	stroke survivor who had a disabling stroke two years previously and who was a keen horsewoman and mother described her desire for participation:<br>I'm lucky I've got children and they want a mum so you've just got to get on with it.... I want to see them get married. I want to be the mother of the groom and wear my posh hat. I want to dance at the wedding. I'm not just going to sit at the top table, I want to dance. [Female survivor, 44 years]<br>Carers' beliefs converged with survivors. They prioritised participatory outcomes, emphasising social participation as desirable, enjoyable and achievable through PA. Social interaction influenced their own and partner's wellbeing, which characterized achievement and progress, as expressed again by the lady whose husband had a stroke five years previously:<br>My husband was a great dancer but then when he had the stroke, he couldn't. But if we went to a dance, and it was a waltz, we got up in a corner and I'd hang onto the back of his trousers in case he fell. I got hold of him, always got a big cheer. It was hilarious. [Female carer, 80 years]<br>In contrast, physiotherapists tended to see participatory outcomes as effects of exercise, rather than as life activities enhanced by PA participation. They acknowledged social and psychological benefits of PA and the importance of survivors engaging in enjoyable activities that enhanced motivation and matched survivors' abilities. However when asked what types of activities survivors should engage in their responses tended to value formal exercise classes, or organised groups where other survivors provided social support. Others suggested that basic activities of daily living were most appropriate. They thus tended to adopt a ''medicalised'' model, in which PA was the source of participation rather than a mediator of participation in wider activities. One physiotherapist working with a mixed neurological and stroke caseload in community rehabilitation described her views:<br>...they're with people that support them and with other strokes so they're getting the support you get with cardiac rehab and pulmonary rehab groups as well... . [Physiotherapist, out-patients, qualified 16 years]<br>In summary, our comparisons between participant groups suggest that whilst carers and survivors tended to locate PA within a survivorship model of broad life outcomes, for physiotherapists it was within a medical model of physical recovery.	Participatory outcomes – encouraged by desires to be better for children<br><br>Family motivating participatory outcomes<br><br>Medicalisaion model rather than recognisign family as helping them

	Control of desired outcomes: capacity, capability, and context<br>Three types of control were identified as central to achieving desired outcomes. Control of capacity for recovery, capability to be active and context are described and compared below how they were perceived by study participants.<br>Control of capacity for recovery<br>We defined control of capacity for recovery as participants’ perceptions of stroke recovery that were within their control. ‘‘Recovery’’ described progress towards achievement of desired outcomes. Many survivors’ perceptions of control of capacity for recovery centred on cause of stroke and recovery mechanisms. Some survivors had little idea about stroke cause, and recovery was considered a natural healing process. These survivors perceived low control and low capacity for recovery; little importance was attributed to PA for recovery. These survivors focused on everyday tasks, often viewing them as effortful. One retired lady who had her stroke fourteen months previously, was mildly disabled and who only undertook some basic exercises for her limbs on a regular basis described:<br>It’s some kind of activity that stops something in your brain but I don’t know what it is...they said just to keep moving and not sit too long but not to over exert myself, that I was just to walk about a wee bit......[Female survivor 73 years old]<br>However, survivors who believed stroke resulted from modifiable medical or lifestyle factors considered purposeful activity a means to control recovery. These survivors reported high control and high capacity for recovery. These survivors, irrespective of disability, were motivated by desire for fulfilling lives, often reporting external recovery drivers. This was detailed by one survivor who had recovered well from a stroke three years before and who participated in several sporting activities with friends and family said:<br>Well...one of the psychological things in my brain, was I need to be, get better for my wife and my family ‘cause they need me...I want to see my grandchildren growing up, you know that was a real driving force for me [Male Survivor, 65 years old]	Understanding exercises help but not understanding pathology<br><br>Personal family drivers for motivation for recovery

	Many caregivers attributed recovery to natural processes. They regarded PA as beneficial to health, rather than recovery, yet believed their partner had capacity to recover. They sought to control return to ‘‘normal’’ life by seeking opportunities for physical progress in various contexts and desire for control of capacity for recovery was implicit in their reported actions. One retired lady whose husband had a stroke three years previously described how she encouraged her husband to be active and to optimise his capacity for recovery:<br>So every, it started at the beginning and I said, right, I’m going to encourage you every day to be what you can be.... day by day, like we set wee goals. When he came out, we set goals. [Female carer, 73 years old]<br>Drawing on their biomedical background physiotherapists believed internal physiological and behavioural factors and external social factors influenced capacity for recovery. One physiotherapist who worked with survivors in an out-patient setting described the influences thus:<br>...the lesion itself. You can’t change that but extrinsically, the care they receive, support from caregivers, how motivated that individual is, maybe their behaviour, their emotional state. [Physiotherapist Outpatient, qualified 26 years]<br>However, other physiotherapists considered survivors’ recovery was primarily controlled through rehabilitation and stroke survivors themselves had little internal control of recovery, as described by one therapist who worked in a mixed out-patient and community setting with young patients:<br>It [recovery] doesn’t happen on its own. Some patients when I go home, it will get better, so it’s trying to explain it doesn’t get better when you go home. It doesn’t get better if you just sit and wait for it to happen either. There’s got to be facilitation. [Physiotherapist Community Rehabilitation and out-patients, qualified 12 years]<br>Physiotherapists tended to believe survivors’ intrinsic motivation was fixed by social context and ability to control capacity for recovery was limited by motivation. Even when motivated in rehabilitation, few survivors were thought to maintain motivation to be active after rehabilitation. A physiotherapist who worked in the community with a mixed caseload saw motivation in this way:	control of capacity for recovery by family encouragement<br><br>not changing diagnosis but changing their recovery pathway via support systems

	I think it's not very usual in my experience to find someone who's been inactive, had a stroke, and then want to be really, really, active. I think they probably are prepared to make some lifestyle changes, but the reality is when they get home they are sucked back into that lifestyle...[Physiotherapist, Community Rehabilitation, qualified 11 years]<br>Control of capability to be active<br>Survivors reported perceptions of factors influencing their physical and emotional capability for activity. Capability was conceptualised by what they believed they could do. Factors that controlled perceptions of capability included physical and emotional effects of stroke; effort of activity; previous exercise experience; mood; communication; co-morbidities; and age. However, desire, determination and confidence to overcome stroke appeared to mediate appraisal of these factors and determined survivors' control of capability and motivation to be active. Survivors derived confidence for PA from pre-stroke experience of PA and benefits observed from activity after stroke. Survivors with social drivers to recover expressed strong determination to overcome the stroke, which enhanced control of capability. These survivors frequently sought ways to be active, irrespective of disability. One retired survivor with low disability who had his stroke two years before and whose activity focused on walking said:	
	Because I want to get better and I think that's where the determination comes from, the fact you want to get better so you do things you're supposed to do in hoping they will work. [Male Survivor, 78 years old]	Hope facilitating control of capability to be active
	When experiences were positive, control of capability was enhanced by increased confidence. One survivor, who was still disabled from a stroke 18 months previously and who participated in a range of activities including stroke exercise classes and bowls said:	
	It's getting confident to do things. I find the first time you do it, you are a wee bit apprehensive, but then you've done it. You've achieved it and the feeling is brilliant, so you don't think twice about doing it again. [Female survivor, 70 years old]	Highlighting repetition of activities to improve confidence
	However, survivors were discouraged from being active where experiences were poor and confidence was lowered and control over capability to be active was diminished. One lady, who was disabled with stroke 2.5 years previously and had a number of comorbidities explained the effect of bad experience on her confidence:	

	Yes, I thought I could go to the gym and surely I would get better there. ''No''. So I then bought me a Wii...I couldn't do it and I did it all wrong and I went and hurt myself on it. I hurt my back. So that was the end of the Wii.... I can't do it [Female survivor, 68 years old]<br>Many carers' supported survivors' motivation by identifying, supporting and monitoring goal achievement, despite physical disability. They did this to enhance survivors' confidence and control of capability for activity. Carers were motivated by care for partners, desire for continuous improvement and return to valued shared activities.<br>....sometimes the carer has to say no that's something you can do, or no, we're definitely going to do this [Female Carer, 80 years old] ...The carer has to take a back step quite often...stand back yeah, keep an eye on them without any accidents...it gives them confidence they've done....[Male carer, 73 years] ...Once they've done it, they know it [Female Carer, 80 years old]<br>However, tensions emerged when carers' and survivors' perceptions of capability diverged. This led some carers to adopt persuasive approaches to influence survivor motivation and facilitate survivors' capability to be active. As lady whose husband had experienced a disabling stroke three years before described:<br>But he was, after we had a terrible fight when I threatened to send him back to hospital, because he was just sitting there and not speaking or doing anything, he began to realise, well, maybe he should. So we started to get out and about again... . [Female carer, 77 years old]<br>Therapists' perceptions of control of capability diverged however. They often viewed survivors' motivation as fixed and unmodifiable believing it outside their role to influence behaviour change, feeling ill-equipped to address motivational and social barriers to PA. Only one physiotherapist reported providing younger survivors in hospital rehabilitation with the opportunity to try out different activities. Efforts to support survivors to enhance control of capability after rehabilitation were often limited to information about exercise services. Two therapists, one who worked mainly with elderly survivors and another who worked with out-patients described issues with motivation thus:<br>You get certain individuals that are completely motivated and will go to any length to get to where they want to go and that's great, and then others, it's maybe their motivation slips and they just see barriers and you can't, because it's getting out your job remit [sic. area of responsibility]. [Physiotherapist Community Rehabilitation, qualified 16 years] Resp 2: I think	Negative experiences impacting on motivation and confidence for activity<br><br><br><br><br><br><br>Carers encouragement and allowing for support to safely undertake exercise and confidence improve<br><br><br><br><br><br><br><br><br><br><br><br><br><br>Outcome- stroke survivors disabling themselves due to lack

	that's where the neuropsychology would be great because so many patients, it's a life changing thing they've gone through and it's maybe changing their thoughts about exercise pre-stroke as well as post-stroke and it's hard to do just as physios. [Physiotherapist, Acute in-patient, 8.5 years]<br>Control of context<br>Survivors' physical, social and environmental context influenced PA participation. To be active, survivors needed to control this context. Transport, knowledge and timing of PA and weather provided the physical environmental context for PA participation. Social context was support from health or exercise professionals, family and other survivors. Typically, social support enabled survivors to control physical barriers to PA. However, social support from family could be over-protective or directive and not always helpful. One survivor who was retired had experienced a disabling stroke 15 years previously and who undertook little PA said about carers:	of sense ability and confidence
	As I said to you they either wrap you in cotton wool which is no a good thing, but that way you, you become an invalid then don't you, and the last thing I wanted to be was an invalid. [Male stroke survivor, 72 years old]<br>Another survivor whose stroke was one year previously who walked and cycled reported frustration with directive relatives:<br>...she's told me, 'You should cut down, you shouldn't be doing that.'...So, how can she judge that?...I was annoyed. [Male stroke survivor, 58 years old]<br>Many carers' sought to complement survivors' PA needs by managing the environment through pragmatic problem solving. They provided physical support including assistance with physiotherapy; environmental support including providing transport, access to leisure centres and suitable venues for community activity. Carers provided social support by encouraging attendance at groups and often attending with their partners. Loss of control over context, for example by reluctance from survivors, or unhelpful leisure staff, led to frustration and disappointment for carers and survivors. One carer whose husband had a stroke seven years previously that left him very physically disabled reported:	Outcome - over-protective or directive

	P was referred to a gym and we went in. Oh well we haven't got people to look after him.... We can't do anything for him...there was nobody there to take the responsibility or had time for him. We weren't in five minutes and it upset D. [Female carer, 61 years]<br>For physiotherapists, context was often defined by survivors' physical ability, underpinned by risk assessment and safety concerns stemming from safety responsibilities as healthcare professionals. They often adopted directive approaches that could limit interactions with carers to concerns about survivors' safety. A physiotherapist working in a stroke rehabilitation unit reflected this perspective:<br>...we're very much focusing, I guess, on the safety aspect, how is that carer going to assist.... so we don't talk about it as saying, this is exercise and this is the level of activity you're allowed to do. [Physiotherapist, acute in-patient, qualified 22 years]<br>As one physiotherapist who worked in a rehabilitation unit suggests some physiotherapists were wary about exercise classes outside the NHS, stemming from safety concerns for patients.<br>I think we need to have good knowledge of what the class actually involves. Go and actually see the class and see what the facilities are like and know what they are doing in the class to know if it's going to be appropriate for patients as well. [Physiotherapist Outpatient Rehabilitation, 18 years]<br>In summary, carers and survivors shared overlapping and congruent ideas about control of recovery, capability and context. Physiotherapists tended to diverge however believing that survivors and carers had low control over achieving desired outcomes. Physiotherapists expected clinical, physically orientated, directive strategies and information could control survivors' achievement of their desired outcomes. However, data suggest a range of more complex contextual social, environmental and behavioural factors influence survivors' participation in PA. In the following section, roles and strategies used by carers and physiotherapists to control achievement of outcomes are compared.<br>Carer role and strategies<br>Our data suggest that carers were facilitators in the setting and supporting of PA goals shared with the stroke survivors. They adopted strategies for goals orientated to life tasks and roles, motivated by care for their partner, desire to see continuous recovery, and to resume shared	Barriers including support from public sources (gym)

	pre-stroke life activities. Their role was influenced by uncertainty about potential recovery, by timescales for recovery, and by hope it would. The carer of the gentleman who was severely disabled by stroke five years before reflected this view:<br>There's always hope they do improve and just try and be positive about it and do what you can for them [female carer, 61 years]<br>Many carers adopted motivating and monitoring roles, assessing process and progress towards goal achievement. Monitoring of process stemmed from desire to facilitate survivors' autonomy to undertake activities combined with instinct to monitor safety. Carers practised ''safe autonomy'' to enhance survivors' selfefficacy for activity, whilst assuring themselves their partner was safe. One carer of a lady who had a stroke 17 months before and who was initially severely disabled said:<br>The carer has to take a back step, quite often. Stand back, yeah, keep an eye on them without any accidents. It gives them a bit of confidence they've done. [Male Carer, 73 years]<br>To enhance survivors' confidence in activity, several carers reported monitoring progress, which was often slow, by noting and illustrating change to survivors through targeted feedback to encourage activity. A carer of a survivor who was disabled by stroke 17 years previously illustrated his approach:<br>...it's difficult when you are living with someone to see any real progress. So I would write down each week things she was doing and compare that with previous weeks and it's amazing actually what the difference there actually is. [Male carer, 64]<br>Physiotherapist role and strategies<br>Facilitators of self-directed activity<br>Physiotherapists viewed themselves as rehabilitation specialists adopting roles as facilitators of independent activity for survivors' transition to self-directed activity. However, their biomedical paradigm meant activities they supported focused on the body function rather than life-enhancing activities, valued by survivors and carers. Physiotherapists provided information about community exercise services and facilitated goal setting, but often on their terms. Their strategies were influenced by perceived boundaries of their role, about survivors' motivation and by their prioritisation of risk minimisation and safety. Most therapists viewed information provision and referral to other agencies as their primary strategy to facilitate	Having confidence by taking risks and pushing self<br><br>Maintaining diaries as a measurable goal setting

	survivors' PA. A physiotherapist who worked in community rehabilitation with patients who had a range of conditions reflected this approach:<br>Let them know what's out there, what they can access, what they can go to, what they can do in the home on their own....[Physiotherapist, Community Rehabilitation, qualified 25 years]<br>Nevertheless, some did focus on more patient-centred goal setting to support uptake of PA, as reflected by one therapist who worked with younger stroke survivors in out-patients and the community:<br>I would sit down with them afterwards and have a one to one session afterwards and say, okay, right, do you want to be part of a class or would you prefer to go and exercise on your own, what suits you, what do you enjoy? Is it going to the gym, is it going out for a walk? [Physiotherapist, Outpatient Rehabilitation, qualified, 12 years]<br>Others were directive and unclear about whether survivors complied with instructions. This inability to rely on survivors' reports led to uncertainty about PA recommendations, as illustrated by a therapist working in rehabilitation:<br>You say right, just try these, do this amount and this amount. You go away and come back. How did that feel? You don't know if they've actually done them or not. [Physiotherapist Outpatient Rehabilitation, 18 years] Although physiotherapists saw their primary role as facilitators of independent activity, in contrast to carers, they described few behaviour modification techniques. They expressed uncertainty about how to motivate survivors to be active.<br>...what we're not doing is addressing the people that don't and why don't they and what are we missing out and actually motivating and changing people's behaviours, how they think about activity and exercise as well. [Physiotherapist Outpatient Rehabilitation, 18 years]<br>Although physiotherapists discussed goal setting in the context of rehabilitation, only one described strategies for goal setting and monitoring of PA:<br>I get them to use a calendar, get them to tick the days they exercise, so in the week, they become focused because some of them are kind of housebound. [Physiotherapist, Community Rehabilitation, qualified 16 years]	goals orientated to life tasks and making them measurable

	Physiotherapist's perceptions and approaches to address survivor motivation stemmed from beliefs about the boundary of their perceived role as rehabilitation specialists, as reflected by one physiotherapists who worked in an acute stroke rehabilitation unit:<br>I think there's probably quite a lot of them we can't address as physios.... That's not really our role to be doing...but it's somebody within leisure doing that role. [Physiotherapist Outpatient, qualified, 22 years]	
11. Mumby & Whitworth, (2013)	Personal narratives<br>The processes underlying participant experiences throughout intervention were complex in nature, being derived in part from direct reflection by the participants, in part from observing others and shared experience, and overall from a synthesis of multiple narratives. These processes were considered to relate to adjustment rather than recovery as participants with aphasia had chronic impairments that were no longer being addressed by rehabilitation services, while participants without aphasia experienced their own adjustment from a different perspective. All participants were observers of others' adjustment. The main focus in reporting the findings is the accounts of people with aphasia, although the thematic network was derived from all the participants.<br>Core themes and sub-themes Five core themes emerging from the thematic analysis structured the more detailed exploration of the experience of participants longitudinally in relation to the process of adjustment to aphasia. These included intervention type, effectiveness, barriers, facilitators and QOL. Mumby and Whitworth (2012) previously explored perceptions of the intervention by focusing upon effectiveness in terms of intervention type. The current paper explores the three other core themes in more depth, looking at barriers and facilitators in the context of the intervention, and relating these to the experience of living with aphasia and QOL. The account of adjustment processes was reached by reflecting on how the main sub-themes reflect changes over time, cross referencing them with coding of the core themes. As improved QOL may be considered the primary outcome of positive adjustment to chronic aphasia, the core theme of QOL provided the main focus for the analysis. According to its sub-themes, QOL is derived from participation, a sense of worth, and degree of adjustment (see thematic structure in Appendix E). The narratives highlighted that, in terms of	Sharing experience and observing others – related to adjustment rather than recovery<br><br>Pt could recognise other improvements or changes but not their own<br><br>Amount of doing ADL's isn't important, but the type of ADL achieved<br><br>2 below by encouragement from peer support

	participation, the amount of participation in activities was less crucial than the degree to which participation was achieved with a sense of worth. Sense of worth was an overarching theme, encapsulating sub-themes that had personal, social and environmental aspects. This concept included a sense of self (knowing and being known) and a sense of purpose (where activity had personal, religious or community meaning) and was encouraged by peer support. The two themes of participation and sense of worth were integral to the consideration of QOL in terms of adjustment processes. The barriers to achieving QOL fell into several distinct categories: barriers to a sense of worth were mainly personal, barriers to participation were mainly social, and barriers to adjustment were multi-faceted. The underlying adjustment processes will be described below, specifically in terms of facilitators overcoming barriers and of personal and emotional adjustment.<br><br>Overcoming barriers via facilitators Overcoming barriers emerged as one of the main processes in adjustment to aphasia. There were five main barriers to quality of life with aphasia: impairment, internal emotions, isolation, inaccessible systems and ignorance, the first four of which can be related primarily to domains in the ICF and the A-FROM, with some overlap, and the last one implicating all the domains.<br>Overcoming impairment (ICF: Body functions & structures/severity of aphasia). Impairment was the most frequent of the barriers emerging within the narratives, with the development of strategies being the key facilitator. Participants, all with chronic aphasia, still hoped for improvement in their aphasia and talked of "getting better" despite little or no change in their aphasia severity during the intervention period (intervention was not designed to address impairment). This frequently resulted in a tension between accepting impairment and maintaining hope. Moreover, the participants identified benefits in "getting around" impairment, such as setting compensatory goals in the "What Next?" groups, and practising new strategies in both "safe" environments and with others with aphasia (peer support and sheltered courses). The development of internal strategies for coming to terms with impairment will be discussed in relation to internal emotional barriers (see below). In summary, adjustment to impairment was reflected in finding and adopting compensatory activity.	Reclaiming an identity and self-assessment<br><br>Sense of purpose within personal, social and within an environment<br><br>Adjustment process required – participation and sense of worth<br><br>Narratives of Hope in getting better<br><br>Difficulties maintaining hope due to impairments<br><br>Adapting to impairment to facilitate coping strategies

	Overcoming internal emotional barriers (ICF: Personal factors). As raised earlier, barriers and facilitators are stated as "not applicable" for personal factors within the ICF, as these are cast in terms of the environment. Analysis of the interviews, however, showed that some barriers originated from within the person rather than from the environment, forming part of the adjustment process. The narratives highlighted particular emotional reactions emerging during the post-stroke journey, both negative and positive (coded within the theme QOL in the sub-theme of adjustment). According to models of grief, negative emotions are to be expected as part of the process of moving forward but, as seen in the narratives, also have the potential to limit progress if they are too dominant and act as barriers. The evidence from the narratives suggested that the adjustment processes involved an emotional shift from negative to more positive emotions, in relation to transforming the sense of self and acceptance. This shift will be explored further below under the heading of personal and emotional adjustment. Overcoming isolation (ICF: Relationships, activity & participation). Participants with aphasia expressed concern about others with aphasia being isolated, having experienced this themselves. The key facilitator was the provision of help in the form of personal contact through volunteer support and tailored programmes of activity agreed with people with aphasia and carers to increase participation in community life. Personal involvement and relationships built up over time were viewed as the key to overcoming isolation rather than simply "getting out (of the house)", and this aspect was linked with a developing sense of self (part of QOL). The importance of quality rather than quantity of participation was stressed by the people with aphasia. The adjustment processes therefore involved supported social reintegration, with increased participation in the activities of everyday life. Overcoming inaccessible systems (ICF: Environmental factors). The barriers raised by existing systems within services (e.g., requiring people to make telephone or email contact rather than personal contact) restricted the ability of people with aphasia to access services and local facilities. Public transport was highlighted as an example where reduced access for people with aphasia often occurred (e.g., due to difficulties with numbers, timetables, completing forms, or asking for a destination), made more difficult by coexisting physical impairment or when confronted with the negative attitudes of others. Facilitators were aimed at both raising awareness within organisations to bring about improved access and empowering people with aphasia to	Emotional barriers preventing and facilitating recovery due to motivation and QOL.<br><br>To facilitate recovery Pt need to accept impairments and be positive<br><br>Personal contact with carers or community life aided reduced isolation<br>Building of meaningful relationships rather than sampling being within the public<br><br>Access to local facilities or services due to environmental factors being a barrier

	campaign for recognition and change. The adjustment processes therefore concerned increasing independence (linked with the personal processes of increasing confidence and self-determination), trying "new things", and ultimately modifying environments.<br>Overcoming ignorance (impact in all ICF domains). Ignorance about aphasia was identified as a key barrier for people with aphasia, having an impact in all domains. The process of overcoming this barrier was twofold: (a) facilitatory activity (from within the intervention or from the participant) for raising awareness, and (b) a subsequent positive response from those concerned, resulting in increased insight. Information could be presented but still ignored: PWA2: I went into the police station and I didn't have a drink for two days right. R: Hm. PWA2: And they say's "you're drunk". R: Ohh. PWA2: And I says "I'm not drunk" and it was a Saturday afternoon, Saturday 2 o'clock afternoon. R: Hmm. PWA2: I says "I'm not drunk", you know, you get people come in here at 2 o'clock in the morning [unintelligible] you know and em, I got me stroke card out and they says "I don't care if you've had a stroke" and I got me aphasia card out as well [gestures putting card down on table]. Raising awareness was not confined to those without aphasia (such as volunteers and staff in other organisations) but was reported as necessary for both those with aphasia and their carers. The overarching facilitator of raising awareness was reported to act in parallel with the other facilitators in overcoming barriers. People with aphasia valued personalised information about the condition and signposting to sources of help and resources. Equipping them with knowledge about aphasia was part of the process of empowerment. All participants were empowered through increased awareness to make changes to their behaviour and that of others. Participants with aphasia reported moving towards a greater acceptance of life with aphasia, and those without aphasia reported developing greater tolerance of aphasia in perceptions and activities ("patience", in the words of the carer). People with aphasia who initially reported dependence on the facilitators of help and support, later emphasised greater independence through developing strategies and sharing experience, progressing towards helping to facilitate recovery for others.<br>Personal and emotional adjustment<br>Evidence from the narratives showed additional processes, specifically concerning adjustment to the ongoing loss associated with aphasia, including restricted independence, also relating to physical loss. All participants talked about loss in terms of impairment but there were also	Improving QOL by trying new things, adapting and taking risks – modifying environment<br><br>Lack of understanding within the community surrounding<br><br>Raising awareness of Aphasia aided overcoming barriers in life<br><br>Aphasia suffers have greater acceptance of life.<br>Carers more accepting as they experience it<br>Sharing of experience as a strategy of coping

	descriptions of loss in personal terms: PWA3:“Hmhm. Well you can’t, it’s different altogether. You know, you feel a different person altogether [gesture to self]”. Moreover, there were losses in terms of participation described by all participants, and less prominent but still present, reference to loss interms of the wider environment. The sense of loss concerned all aspects of life. For example, when questioned about what it is like living with aphasia in her first interview,PWA3 responded: “[laugh] Start with this [gestures everything][laughs]”. The view that aphasia adversely affects all aspects of life was reported by the participants without aphasia, a kind of “fragmentation” in opposition to the movement towards “wholeness” as shown in Figure 1 above. This fragmentation was described by PWA2 as a lack of coherence in his everyday functioning: “I want to work, to be perfectly honest with you but I’m kind of (.)I’m just getting through the summer .... Ah man, you just. I cannot ...I get frustrated I cannot explain things, or worry I cannot explain things”. It was described by the carer as a breakdown in relationships: “But (-) a baby (-) a child progresses where (-) when you’ve got aphasia you go(-) it takes longer and you don’t get (-) back to where you were before .... I just get on with it”. Some fragmentation concerned the variability and unpredictability of aphasia and the continued need to readjust; in the words of PWA4, “everything is changing all the time”. Adjustment involves resolution of such fragmentation by reforging links between different aspects of life.<br>Emotional factors. As well as concerning physical, personal and interpersonal loss, adjustment also involved emotional reactions in relation to loss. (These excluded those emotional changes that are the specific sequelae of stroke such as lability (Code et al., 1999) described as a separate issue by the participants with aphasia.) Within the interviews, most references to negative emotional reactions concerned fear (direct references to fear, and the related themes of worry and anxiety) a danger (with its more extreme form, rage, as well as more muted annoyance and frustration). Most references to anger included the term “frustration”, the term used in the grief model, and the theme off ear was very closely linked with sub-themes about uncertainty (especially about the future) and vulnerability. There were minimal explicit references to denial, possibly due to the participants in the study not being in the early stages of adjustment to aphasia, although it was still present. Interestingly, denial (involving restricted awareness, or choosing to ignore issues) continued to evolve alongside the other	Loss of previous identity and now identifying as a different person<br><br>Identifying that Aphasia has affected all aspects of life, fragmented reality<br>Fragmentation causes the patient to believe everything is a struggle and a challenge and that no part of them is the same, requiring adjustment for resolution to reforge links to previous life<br>Loss – its understated that the grieving process for loss of identity is separated issue<br>Uncertainty and vulnerability root cause of Anger and frustration<br>Denial linked to disengagement and

	emotional reactions within participants during the course of the study. The earlier interviews and the participant reflections about their own or each other's recovery sequence also provided illustrations of denial as it related to fear or anger. For example, denial was manifested as disengagement, withdrawal or avoidance as a means of coping with the fear of social contact (e.g., PWA3 was very reluctant to go to groups) or coping with the loss of skills by avoiding situations requiring these (e.g., PWA2 initially identified multiple reasons for not using written language). Denial was also manifested as over-preoccupation, rigidity or misdirected activity (which may be aligned with anger) to avoid positive adjustment, observed in others by participants who refused to modify their own behaviour. Reducing denial was linked with the sub-theme of raising awareness (see above, overcoming barriers). The interviews suggested that in the process of adjustment to aphasia, the participants progressed from negative emotional states and reactions (primarily fear and anger), to more positive ones (themes of humour, expressing relief, motivation and determination being well developed). The progression included a variety of positive outcomes (themes of increased independence, enjoyment, hope, looking to the future and aspirations, openness and understanding (of others), finding a sense of worth, and crusade) coinciding with increasing acceptance. Unlike the grief model, there was no specific reference to depression in the interviews as a precursor to acceptance. The narratives from the people with aphasia showed shifting emotional emphases, starting from negative reactions such as fear and anger. For example, looking specifically at fear (and anxiety), PWA3's thematic sequence may be summarised as follows (shown with the corresponding interview number containing references in brackets) with expressions of fear throughout but more positive emotional reactions increasingly voiced: Fear (1 to 4)→Tearfulness (1)→Verbalisation & rationalisation (2 and 3)→Enjoyment, Hope & Increased independence (3 and 4). Initially, PWA3 was openly tearful when discussing how the aphasia had stopped her being active. PWA3: "(-) Well just everything really (-) [big sigh, teary]. I find it difficult to meet people". She emphasised verbalising her fear and anxiety (Interview 2and3) ,particularly about communication and going out ,and later rationalised some of her problems in terms of normal ageing. She was proud about her achievements in the Art exhibition (Interview 3: "a little bit heaven"), and hopeful at being "a little bit better"(Interview3)and" along time yet ...each time a bit more"(Interview4). In the final	withdrawal from coping or social contact<br><br>Wanting to fail to prove a point – its hopeless<br><br>Transition from Negative anger and fear to acceptance through hope and sense of worth – openness<br><br>Pathway from negative to positive hope and increased compliance<br><br>Pride from achievements

	interview, she was a little more positive but found it hard to maintain this positivity because of repeated setbacks related to ill health having the potential to lead her back into fear and anxiety. This type of sequence was borne out in other participants. For example, PWA2 revealed his fears changing (from aversion to the telephone, to fear of social contacts, to fear of large gatherings) and resolving as evidenced in the following stages in successive interviews: Fear (1 and 2)→Verbalisation, Increased independence (2 and 3)→Enjoyment, Looking to the future (4 and 5). The theme of anger (or frustration) was also traced in the participants over time. For example, in PWA2 the following sequence was emphasised: Frustration (1 and 2) → Humour (3) → Channelling drives/Attribution of blame (4) → Crusade (4). Frustration for PWA2 concerned his inability to work as a head chef, difficulty getting words out (Interview1), and at fatigue after talking (Interview 2). By Interview 3, he was using humour to divert some of his frustration, and by Interview 4 he was showing greater interest in the experiences of others, diverting some of his anger into concern for what was happening to others with aphasia, blaming ignorance from communication partners ("I could have killed her", Interview 4) and being determined to set things right, the theme of crusade being found alongside references to acceptance and self-worth. The progression towards positive emotions and outcomes aligned with the" positive personal outlook" identified by Cruice et al.(2010).PWA2:"Em, I've got to be positive ...and just move on, do you know what I mean? It's like er, I've always been a positive person but I've got to be more positive". Positive outcomes included the development of a sense of crusade in some participants (PWA1, PWA4 and, latterly, PWA2 helping to campaign about aphasia and help others). Conversely, the concern for others with aphasia expressed by PWA3 could be traced to her own experience of fearfulness and the hope that future intervention services would prevent others from becoming isolated, rather than becoming an active campaigner herself. Positive outcomes also included increased sense of worth (found through volunteering, for example, see "participation", Appendix E). Two key processes underlying the progression within personal adjustment were reflection and rationalisation, which are elaborated below.<br>Reflection: Looking back, looking around and looking forward. The analysis revealed three stages in thinking about living with aphasia: Looking back, Looking around and Looking forward. These three stages interwove two important sub-themes within the adjustment	Frustration and reoccurring set back due to looking back at previous identity<br><br>Partner is a scapegoat and who emotions are expressed to<br><br>Positive attitude facilitating acceptance<br><br>Recognising the loss of previous identity and abilities

	process related to coming to terms and growth and their subthemes (see Appendix E).<br>"Looking back" comprised reminiscence and reflection including reassessments of past life, remembering the recovery sequence in relation to the length of time since the stroke, and in relation to loss. "Looking around" was linked with the provision of peer support, ascertaining impairment levels by comparison with others, and reflection on what constitutes normal ageing. The sub-theme of transition referred to a shift in behaviour observed as ongoing at the time of the interview. "Looking forward" involved personal growth, demonstrated in independence and confidence, and specifically in new things, such as broadening horizons and looking to the future.<br>Rationalisation and finding meaning. The narratives highlighted rationalisation (i.e. consciously thinking things through, including verbalisation to make sense of life) as another key process in personal adjustment. The interviews endorsed the value of the "What Next?" intervention groups, offering a visual framework and structure for reflections by supporting participants in their verbalisation of ideas. Being able to express and discuss fear and anger (to professionals but also to others in a similar situation) was identified as part of the shift towards taking control and reaching positive outcomes. Moreover, the task of "Looking back, around and forward" was facilitated by an integrated consideration of all aspects of life), and to envisage these aspects together when looking forward was part of reforging links between the different aspects of self and the reworking of self-image. A final aspect of the adjustment process was a search for meaning, including asking the reason for the loss—asking "Why?". In its most rudimentary form, this was represented in the attribution of blame, observed in all the participants with aphasia (but also found amongst those without aphasia; see Table 4 below). Verbalisation played a part in moving on from the attribution of blame, by reasoning "it happened because", and the two main strands of this within the narratives were (a) to help others or change things and (b) to prevent others from going through the same pain. Through "finding meaning", both of these strands were associated with being better able to accept living with aphasia. By the nature of aphasia, verbal rationalisation is particularly challenging but constituted an important part of adjustment, in which peer support encouraged shared vulnerability and experiences, and a sense of worth. Participants referred to the resolution of blame, guilt and shame as acceptance.	Once acceptance of condition is recognised then shift towards taking control – positive outcome<br><br>Looking back, around and forward strategy to link the fragments of the fragmented life to establish wholeness<br>Attitude of blame observed - verbalisation through reasoning helped move on from this<br>Able to accept personally by helping other come to terms with experience

12. Nanninga et al., (2017)	Context in mobility practices In this study, we focused on three contexts in which mobility practices are played out: the rehabilitation centre, home and community [24–26]. We found that our participants' mobilities are enabled or disabled in everyday activities, both by physically and cognitive opportunities, and by human and non-human obstacles. During inpatient rehabilitation, most participants learned to move around fairly safely and independently in the clinic. When back home again, however, many reported having difficulties with this aspect in their own house. They said that the physical environment in the clinic was adapted to their needs, unlike the impediments at home. Staircases were a frequently cited example. John, for instance, explained that mobility training in the clinic had not really prepared him for the stairs he had to climb at home.<br>John: For example, we had practised on the stairs, but the stairs at Beatrixoord were very different from at home. They're not the same at all. It might have been easier if someone had come with me to practise at home, for safety reasons to see how it all went. Then you'd know what to do. Now I had to work it all out by myself, which I found very difficult in the beginning. It was also hard for my sons because they also didn't know what to do. At home, John felt that he had to work out for himself how to go up and down stairs. Especially troubling was the fact that the stairs at the clinic were straight, whereas those at home turned a 90degree corner. This meant that some steps were narrower on one side and difficult to negotiate, and John was afraid of falling when going downstairs. Home modifications, such as installing grab handles in this case, only solved part of the problem, since John still had to take the stairs. It was not only the physical environment (stairs, corner) that differed between home and the clinic, however. The social environment was also different. At the clinic, therapists taught John how to manage the stairs and they were always close at hand, whereas his sons were not always at home, and when there, did not know how to help their father take the stairs. Because of the uncertainties John experienced, he decided to move to a single-storey apartment. Like John, many of our participants had difficulty applying the skills learned in a therapeutic environment to their own living environments. Mike developed various strategies for taking the stairs:<br>Mike: It takes so long because I have to take the stairs slowly and then there's no problem. And going down again, from upstairs to downstairs, on a good day I can walk down as well as	Post discharge concerns home set up not adapted to their needs<br><br>Lack of post-discharge support – physically un equipped for home life<br><br>Lack of moral support and isolation from help increasing fear of task<br>Changing the environment around to cope with limitations

	anyone. But if I hear the doorbell and I have to go down quickly, I go down backwards, to be on the safe side. Mike was able to adjust his pace and the way he tackled the stairs, depending on the situation, which shows considerable flexibility. It was not just the staircase, however; all his pre-stroke routines took a lot of time and energy. He therefore chose to move to a house with a downstairs bedroom so that he could free up time for more pleasant things than going up and down stairs. When going outdoors, our participants faced more and different obstacles than inside the home. Typically, these obstacles were caused by their hemiplegic body. There were also cognitive problems when dealing with busy situations. Rose told us how the physical environment outdoors did not accommodate her hemiplegic body:<br>Rose: I just don't feel confident when I walk. The paving stones are hopeless, you soon discover that if you can't walk properly, all paved paths are crooked. That means that I go out less often than I could. Because footpaths differed from the even paths at the rehabilitation clinic, Rose went outdoors less often than she would have liked after returning home. Ben explained that although he did go out, he sometimes struggled if something unexpected happened, such as having to go to the second floor of a shop without a lift.<br>Ben: Well, in shops with a lift, I take the lift. Because people are used to walking on the right-hand side of the stairs. I can use my right hand, but it drags a lot. So I really have to try and get my arm up onto the railing and raise my leg as well, which is very difficult. I should really walk on the left if I take the stairs. Ben, who suffered from a right-sided hemiplegia, took the left side of the stairs because that gave him more support for his body. However, that meant having to move against the traffic flow, as people in the Netherlands keep to the right. Therefore, although most shop staircases are wide and therefore resemble staircases in rehabilitation clinics, they are not used solely by rehabilitation professionals and peers, but by people who are unaware of the special needs of hemiplegics. Similarly, other participants' stories show that post-stroke bodies are often not acknowledged and accommodated in public spaces, in both social and physical respects. Sarah, for instance, would avoid crowded places or ask her husband to accompany her.<br>Sarah: Then I go out onto the street with my husband because I've fallen over a few times….Busy places and such, people push against you and don't watch where they're going. I tend to avoid it, escalators and that sort of thing … In my case, my husband also needs to feel	Adapting physical strategist to problem solving<br><br>Physical inabilities increasing fear of falling and demotivation to leave house<br><br>Difficulties to adapted to situations outside of routine<br><br>Adapting routine by observation of others to complete tasks<br><br>Physical and social barriers to success during tasks due to public awareness<br><br>Adapting to situation by reliance on family for support.

	like going out with me. Because Sarah was afraid of falling in public, she became dependent on her husband's support. The above stories demonstrate the challenges involved in moving around in the outdoor physical and social environment. Many of our participants avoided public places, or when they had to go out, they planned carefully how, when and with whom they would go. We found that it is more difficult for stroke survivors to achieve satisfactory ambulation in public than in private spaces, and more difficult in private than in clinical spaces. This indicates that stroke survivors should not just comply with lessons that professionals set out for them in the clinic. Rather they should be supported in coordinating, transforming, dismissing, and attuning the taught lessons to ever changing socio-spatial contexts outside the clinic.<br>Ambivalences in mobility practices Mobility is important for balancing work, family life, in leisure and pleasure . We describe the ambivalences that our participants experienced in their attempts to be mobile at home and in the community in their post-stroke lives. John, for instance, told us how he juggled to maintain his mobility after his discharge from the clinic: John: I've actually gone backwards because I move a lot less now, only when I have to. The therapy (at Beatrixoord) was like a big stick to keep me moving. I only go outside occasionally now. …I can't get to the shops because I would have to walk and it's too far, it's 500 metres I think. I'm still getting a mobility scooter and a serving trolley, I've applied for them. But it'll take ages before I get them……. Everything depends on my son, who still lives at home. I don't get any household help, because he still lives at home and he also has to do the grocery shopping. That's really difficult because he's away from home for 12hours and has to do it all in the evening. There are several ambivalences in John's story. First, he wanted to go outdoors in order to maintain his walking ability, but when outdoors he was unable to participate in valued activities, such as walking to the shop. This clashed with his wish to engage in useful activities and to give his son some relief from the triple burden of work, household and care activities. John did get a mobility scooter in the end, which enabled him to engage in activities such as grocery shopping, thereby improving his family and community life. At the same time, however, his frequent use of the mobility scooter meant that he walked even less, which led to a further deterioration in his physical condition. The major ambivalence that is played out in John's case is how family and societal roles were served by a mobility scooter, which at the	Loss of spontaneity and requiring planning prior to tasks to manage<br><br>Reliance on therapists to motivate causes lack of compliance independently<br><br>Loss of therapist caused reliance to be shifted to close relative for support<br><br>Attained a mobility scooter to elevate family from responsibilities – Emotional burden facilitating change

	same time worked against his physical fitness, a clinically significant factor. Similar ambivalences surfaced in the stories of other participants. As well as physical impairments, our participants also talked about cognitive impairments that limited their community mobility. Mary, for instance, had become hypersensitive to stimuli after her stroke. Mary was single and had a busy working and social life pre-stroke. However, cognitive impairments and fatigue meant that she could not continue all these activities. Mary chose to continue to work, but that meant she had to quit activities such as regular visits to friends and going to the fitness center. She also had to plan almost all her activities in advance, such as shopping.<br>Mary: When I go shopping I always prepare a shopping list the day before. I know exactly where everything is in the shop. So I write my list based on the route I take through the shop. … And so I arrive, grab a trolley, go into the shop and don't even have to think about it. I grab what's on the list, pay and leave. I never think "Oh, that's on special" or "hey, what's that?" No, that's what I need, I grab it and in it goes. Then I'm happy to be able to go home, ha ha.<br>For Mary, shopping had become a planned, controlled trip. She could no longer take the time to compare different products and get the best deals available, as that took too much of her energy. As well as working and grocery shopping, Mary continued cycling as a leisure activity, although it had become demanding for her post-stroke. She had to think consciously about every push on the pedals to prevent herself from falling. Despite this, she developed strategies that enabled her to enjoy cycling again:<br>Mary: I don't take the time to look around and think, hey this is lovely … No, you think gosh I'm tired or I have to sit for a bit. … Then I find a bench and I go and sit down. …. I always have a book with me or a puzzle book you know, or a bottle of water, I always carry all those things with me. ……It keeps me happy for a whole afternoon. Cycling used to be a leisure activity in which Mary could enjoy both the physical activity and the scenery along the way. Poststroke, however, she had to plan it in advance, including creating opportunities for rest, during which she could look at the scenery. The ambivalence in Mary's story comes from the time and effort she now needed for work, shopping and cycling. As a result, she had very little time and energy to engage in other activities that were valuable to her, such as meeting friends, going to the fitness center and shopping for pleasure. The quotes from Mary and the other participants show how stroke survivors have to be creative in dealing with the ambivalences and tensions	Loss of social connections due to fatigue<br><br>Forward planning to achieve activities<br><br>Creating routine to manage<br><br>Prioritisation during tasks – safety and pacing strategies<br><br>Constant adjustment and adaptation to

	they face in regaining mobility in home and community life. It is this process of endless tinkering, weighing, adjusting, and coordinating mobility practices in each situation anew, that needs to be acknowledged by the rehabilitation team. Indeed, at home everything hangs together, whereas different aspects of post-stroke life are allocated to different team members in the clinic pre-discharge. After discharge, stroke survivors and their families need to figure out how to set priorities in their mobility practices, to achieve satisfactory bodily, family, working, and community lives, to let go of particular pre-stroke pleasures, and to reformulate goals every day and everywhere, every time and again.	surrounding and tasks to be successful and achieve<br><br>Developing priorities and goal setting for coping
	The politics of mobility practices Our third findings-section outlines the inequalities in access to resources, such as personal abilities, a social network and money, between the participants. This underlines the political dimension of mobility [24,26]. Participants experienced differences in accessing health care in general, and obtaining mobility aids in particular. Their stories revealed that it is in fact difficult to arrange mobility aids such as grab bars along the stairs, braces, walkers, wheelchairs, mobility scooters and taxi transport. Many participants felt that they had to work out by themselves what aids they needed, and how to get them. Take, for example, the story of Tom's partner, who explained how they discovered that the mobility aids recommended by professionals at the rehabilitation center – in their case a mobility scooter and wheelchair for community ambulation – did not work well for them at home, and how they struggled to get the right aids.	Self problem solving post discharged to deduce their specific home needs
	Tom's partner: So, you're discharged home, and you're asked what would you need at home, what kind of aids, what kind of support, but at that moment, you have no idea yet. Only when you're at home does it become clear what you need, or what's missing, only then is it possible to say. […] So we needed to change stuff. We got the mobility scooter, we got the wheelchair. But then I asked for a disability parking permit. "Well," she said, "we don't need to apply for that, you'll never get it, it's very difficult". But now, it's what we enjoy most. I put a lot of effort into getting the permit, because you're not allowed to park anywhere these days, and he couldn't walk that far, so I didn't go anywhere. […] So we gave back the wheelchair and bought a smaller mobility scooter. Although Tom did not use his large mobility scooter for months, they had to pay for it every month. And the wheelchair did not work out in practice because Tom's wife, who was much lighter than Tom, had to push it. She would quickly become	Expressing need for OT assessment post discharged once stroke survivor knows what they would need

	exhausted, especially on difficult surfaces. Both Tom and his wife found the small mobility scooter and the parking permit ideal for their community mobility. However, many of our participants did not have the capabilities or support of family members to select and negotiate the aids and facilities that are available from municipalities but are difficult to acquire, such as a disability parking permit. Their mobility at home and in the community remained limited. In particular, the fragmented organization of health care and related provisions was seen as an almost insurmountable obstacle to extending their mobility range and with that, their social participation. Steven's partner explained how she felt burdened by this fragmentation as an informal carer:<br>Steven's partner: You just have to apply for everything yourself, all that paperwork, it's enough to drive you crazy. Those appointments, you have to make the time for it all. He has to go in a wheelchair, in a shared taxi, he needs to have grab bars, he needs such and such. And every appointment is something different. So a grab bar is something different from a modification for the shower, which is different again from another appointment with the council and the share taxi that you need. And that's different again from having the back of the house modified. Yes, you can't keep dividing yourself up and working thirty hours and everything …And another year has just flown by. The fragmentation Steven's partner told us about is caused at least in part by the fact that in the Dutch health care system, different organizations are responsible for different aids and facilities, and there is a lot of administration involved. Stroke survivors and their families have to make separate appointments and arrangements for mobility aids, home adaptations and transport provision. Stroke survivors and caregivers explained that schemes are often not clear and differ from one municipality to the next. This seems to give stroke survivors and their spouses unequal access to resources, which also indirectly restricts their community mobility. As a result, some participants were unable to arrange the mobility aids and facilities they needed in their own living environment, or it took a long time to do so. For example, not all our participants had the resources to get their driving license back or the financial resources to maintain a car. Take Isa, for example, whose social life was hugely affected:<br>Isa: You do lose quite a few friends because you can't do everything independently any more. I always used to drive myself – I went everywhere. I couldn't do that now. And now I'm allowed	Finding that "best practise and advice" not specific to all and how patients adapt to suit their situation<br><br>Unable to control own required help due to barriers from different service suppliers<br><br>Loss of social relationship due to

	to drive again, but I can't afford a car anymore because I've been incapacitated. Although Isa eventually regained her driving license, she could no longer afford a car. The stroke had left her unable to work, which limited her financial resources. Like Isa, many of our working-age participants became unfit for work post-stroke, and received a sickness benefit that was less than the income they had earned when working. This illustrates that participants with better financial resources and pre-stroke working conditions appear to be less home-bound. This section has shown that the political aspect of mobility, in terms of having unequal access to resources, influences the number of outings and activities of stroke survivors and their families. Moreover, the bureaucratic logic survivors and their families are facing post-discharge, complicates the tinkering process that has been articulated in the previous two sections. Such findings would have been easily overlooked when framing mobility as a movement from A to B, or in effectiveness research that isolates a few variables to be able to account them.	barriers affecting isolation- driving<br><br>Participants with greater household income were less housebound
13. Norris, Allotey and Barrett, (2011)	It was not only the impairments of the disease and health seeking that were raised by the participants but the effect they had on an individual's ability to participate in the life they led. Evidence of the consequences of stroke was illustrated in many ways, through the dialogues, photographs and in observation, through which two broad themes emerged. The first was the burden of a disrupted life, in which the direct personal impact of impairments was particularly related to familial and emotional consequences. The second was the rebalancing of life over time. Both illustrate the interconnectedness of individuals to their immediate familial and spiritual context.<br>The burden of a disrupted life<br>The onset of physical weakness and continuing sequelae of stroke limited the individual's ability to function, resulting in the disruption of many day-to-day activities. Difficulties with activities of daily living such as washing, dressing, toileting, cooking and eating were identified by all participants, as the daughter of Filza (F, 55 years old) describes:<br>There were many things [she couldn't do], shopping, couldn't do gardening, cooking, she was just able to sleep. Even to eat was difficult. We had to help her eat … We had to help her to do everything, she can't lift her arm … she had to ask for help even to eat.<br>Arti (F, 46 years old) similarly described her inability to complete tasks independently.	Reliance on Family to complete ADL's and to motivate

	All [activities] are with help, all. If I want to go to the toilet, with help … If I want to go to the plantation … now it's gone … I can't, walking … I feel bad, my heart is broken.<br>Because of the personal nature of the activity, the inability to use the toilet independently was particularly important. This was especially so for the women. However, it was not just the loss of privacy that was of concern, but the burden it put on others to assist with this essential task. Immediately after her stroke Lastri (F, 56 years old) had to be carried to the bathroom to use the toilet. She said, 'I was put on the back like a child'. In the photographic interview she addressed this issue again:<br>This [the photograph] is when I got up from squatting … I was in the bathroom. This is the most important for family life. For a woman, using the bathroom is important. It is easier if we can do it independently … If we can do it alone, we do not have to ask some else to carry us just to urinate or any other bathroom activities. (Lastri)<br>Filza, Arti and Lastri, like the other participants, relied on close relations to assist with their daily care. They were fortunate to have older children or spouses who were able to assist in these tasks although, as Lastri indicates, this was not a burden they wished others to bear. This assistance was often very difficult and physical in nature. While Lastri was successfully carried to their indoor and relatively spacious bathroom by her sons, Arti's daughters had a more challenging task. Their very small toilet was away from the home, along an uneven path and Arti herself was larger than her daughters and physically very dependent. As a result they often had to compromise and use improvised commodes at the bedside. This was very distressing to Arti and created some tension between the family members, although they understood the necessity for this. In other observed families, this assistance was not available or offered. As a result individuals were left bed-bound and soiled for prolonged periods of the day. For the stroke survivors themselves there was one activity the limitation of which had particular salience: work:<br>Interviewer: From all these pictures which is the most important? Pramana: This one. I am unable to work. I want to be able to work but I am unable to work freely and independently … it has a big influence. At that time, I could break the tree branches, but I cannot do that now. I used to take avocadoes. I used to be able to go from one coffee tree to another, now I can't, I have to take a stick with me. It has a big influence to my condition now. I used to go to the	Emotional burden on other to perform tasks<br>Felling of being demoralised and treated differently<br><br>Burden on family to complete tasks, establishing new family roles<br><br>As above<br><br>Lack of this assistance resulted in loss of functions and independence further<br><br>Comparison to previous identity causing sadness

	garden in five minutes, but now I reach there in two or three hours … This [picture] shows when my children were working together, tending the garden. I could not walk to the garden, so then they took me by motorcycle and I was there with them. I only sat down and took care of my grandchildren, while my children were working together … I, of course, cannot work. (M, 66 years old)<br>The ability to work was key to all but one participant. Its loss post-stroke was not just a personal loss but was predominantly seen as a threat to the family. For the participants, the threat was posed not only through current financial insecurity, but also because other family duties, such as educating their children, had to be sacrificed. The following three contextualised quotes illustrate these concerns. Agus, a married father of two children below the age of four years, had been a cart vendor prior to his stroke as well as tending to crops, jobs he could no longer do. He struggled to discuss the implications of these losses for his family. In the first interview he raised his concern about the economic condition of the family:<br>Agus: It [my health] is really, really important … If I was healthy I would get some money for the family … I have to be able to walk [to work] … Now, I am more emotional, quick to get angry … I am getting angry easily, the economic condition is my concern. [Long pause]<br>Interviewer: Can you tell me more? [Long pause]<br>Agus sits for a long time with his head down breathing deeply. His wife, cradling the youngest child, asks how he is feeling. He does not respond. The interview is stopped. Agus' wife was his main carer and with young children was fully occupied in the caring role. She was supportive of Agus and together they pursued multiple health-seeking options in the hope that his condition would improve. But as the months passed and no functionally significant improvement was made, this energy, time commitment and financial investment was reduced, being redirected to the wellbeing of the children and the family as a whole. Faisyal (M, 58 years old) had his stroke in 2006. He lived with his wife and three of his seven children. Prior to his stroke he had been a religious teacher but his income primarily came from selling coffee and vegetables grown on the family plantation. In the following extract he alludes to both the familial and emotional consequence of his lost incomegenerating ability:<br>Faisyal: I think about my children, three of them, because they have to go to school … why other people can send their children to school and I can't, and I must fulfil the needs of my	Loss of previous role and responsibilities<br><br>Emotional state and behaviour causing frustration and lack of patience with tasks being a barrier to success<br><br>Lack of improvement seen thus gave up on trying

	children. Interviewer: What is the biggest problem you face? Faisyal: That I cannot work again, yes it burdens me because my children need a lot of money to pay for their study … It is hard. It may cause them some trouble. Of course there is a problem. They all have to help me.<br>The relationship between lost income and the responsibility for children's education was reiterated by four stroke survivors. Melati (F, 46 years old) had two children still of school going age, but the income no longer earned, now she was unable to take produce from the family plantation to the local market, had resulted in the children being temporarily removed from school:<br>Melati: I cannot work, no, I cannot, I cannot even cook anymore. Melati's husband: Cries, she very often cries. Melati: Just watching the tasks without being able to do them. I cannot help with the children's education, just watching them, and I could not work moreover … they can forget to go there, to school because my economic condition cannot make it possible … it is a responsibility, the heaviest one. There are many … but especially about the children's school, about the expense.<br>Rebalancing of life over time Although the participants noted numerous ways in which the stroke had impacted negatively on their lives, all but two (Sujatmi and Agus) also discussed ways in which the resultant disruption had been remediated over time. Some of the rebalancing of their lives had occurred through recovery of function. Indeed, stroke was seen as a curable disorder and as a result recovery was both pursued and expected (Norris et al. 2010). But this physical recovery, which was incomplete at the time of this research, was also supported by a reconfiguration of roles within the family unit. Melati had previously been responsible for selling the produce from the kebun in the local market. While she was unable to return to this role, she had a number of children who had, over time, taken on the task. This allowed her to spend more time on other household jobs which she was able to manage and her husband also joined her in a collaborative capacity such as helping with cooking. This rebalancing and shifting of roles was apparent in other cases. Prior to her stroke Lastri, a married woman with three children, had worked as a village health worker as well as on the family plantation. She used all her seven selected photographs to discuss her relief that her capacity to be independent in some familial roles now facilitated her children's independence:	Catastrophe narrative, "I cannot" giving up due to emotional and physical burdens<br><br>Accepting loss of old roles but developing new roles within the family<br><br>Strengthen family relationship roles by increased independence

	Lastri: This [picture] also impresses me. Interviewer: Why does this photo impress you? Lastri: Because I can help my children by finding firewood. It means I can already make an effort. It means I need no more help from my children. Interviewer: Why is it important that you have no need to ask the other's help? Lastri: I … because I want to be independent. My children also do not need to help me anymore as they have had to do it until now. All [the photographs] are the same. taking the clothes off the washing line is also important. Especially, in rainy season because my children have to go to college, and nobody is home. Here I can help my children to sweep and to collect firewood. Interviewer: Before you got your stroke, did you often do all these activities – like cleaning the yard? Lastri: I cleaned everything … planting koro fruit and vegetables. Then I just wait to harvest the fruit. Until I got sick … Now of course, I am happy. I can help by working again … I hope my children do not need to help me again.<br>Interestingly, although Lastri's husband was active and devoted, he had maintained his roles in the kebun, while the children (all over 16 years old) had taken on the caring roles. Lastri herself often commented that their care reciprocated the love she had given them throughout their lives. Nevertheless, her relief that she was no longer the recipient in those relationships is evident. In addition to the adaptations made in roles were those made to facilitate prayer. Prayer was a key activity for all the participants and one which was maintained despite their physical limitations. Five of the participants described the alterations they had made to make praying possible. They choose to sit rather than fully prostrate themselves from a standing position; to wash prior to prayer doing tayammum, which is a dry wash rather than the full ablutions (wudhu), and the dress they used. Ramelan (M, 60 years old) used a photograph to explain how he adjusted to periods of sickness during which he could not attend the mosque. Interviewer: Where do you usually do shalat (prayer)? Ramelan: In the mosque … when I am healthy, I do it in the mosque … every Friday is a must. Interviewer: What about the other days? Ramelan's friend: When he is ill, he does it here, inside the house. Ramelan: Yes, yes … I am still doing it although I am sick. I do it in a sleeping position if I cannot sit down. The most important is that I pray.<br>For the participants the importance of prayer had additional significance directly related to their stroke. Three participants discussed prayer as a form of surrender, a way to accept their mortality and put their life into the hands of Allah. Interestingly, this did not stop them from	Sense of pride and purpose in doing<br><br>Family bond strengthening due to illness<br><br>Adapting routine, task driven changes of coping<br><br>Religion and spiritual reasons for facilitating change

	actively trying to change their situation but rather assisted in the resolution of negative feelings. As Pramana (M, 66 years old) stated ‘At first, I felt regret, but what for, so I surrender myself’. A further three participants also discussed the role of prayer in their own recovery, as Melati illustrates:<br>Praying is the most important for me. Insya Allah [by the grace of God] I’ll be better, I pray to God that I will get better, that’s why I feel happy. I hope that through prayer there is a change. (Melati)<br>The role of religion was primarily discussed in relation to personal religious practice. Personal prayer was important to all, but few regularly attended the mosque or had been involved in other religious activities prior to their stroke. Indeed these more social aspects of religion were conspicuous by their absence, with only two participants, both male, concerned with them post-stroke. Ramelan, for example, had been well known in the community for his ability to call people to prayer (adzan). His stroke had initially rendered him too weak to continue this role, which had clear social consequences, as he and his wife record:<br>Ramelan’s wife: Before, when he was healthy, he used to be a religious teacher and a dukun [healer], but now he does not do it. Ramelan: I was sad when I heard adzan because I couldn’t do adzan again. Ramelan’s wife: People ignored him. Ramelan: These last three days I can do it again … I have to do it with deep feeling. Ramelan’s wife: Everybody now comes here when he does adzan<br>Faisyal had previously run an informal group for religious education. After he had made some recovery from his stroke he had chosen to restart this as a priority, albeit on a smaller scale than previously and with support of his wife:<br>It’s very meaningful to me; it’s a service that I could give to people. And it also is a communication between me and people around my life. For example, if one child doesn’t understand the singing [Al-Qur’an], he, she will ask me … I ask them to sing the Al-Qur’an and if they find it difficult to know how to sing, then I’ll teach them from my sitting position. (Faisyal)<br>The examples given illustrate how the disrupted life reformed its continuity in part, through both social (familial) support and adaptations to tasks, including that of prayer.	As above as a means of hope for success<br><br>Loss of identity within a community due to illness.<br><br>Commencing previous religious duties by bringing community to them, adapting for success – reclaiming social identity

14. Price et al., (2010)	Findings<br>The day everything changed<br>Teilhard, anambitious and successful man in both his private and professional life as a physician, scholar, and investigator, was preparing for ski season on Election Day 1984. In his "typical overachieving style", he was doing sit-ups while lying across a chair. Afterwards, his wife noticed that he was not using his left side so she took him to the hospital; he had had a stroke. He spent time in acute care and then a rehabilitation unit. During this time period, he stated that the prevailing rehabilitation theory held that getting function back in the affected upper extremity was extremely limited. Instead, he worked extensively on rehabilitating his lower extremity, learning how to walk with a cane and eventually returning to downhill skiing and other outdoor sports. Otherwise, Teilhard did not speak in depth about his experiences with occupational or physical therapists. His narratives revealed the following characteristics that seem compatible with resiliency theory (6).<br>Social support<br>Teilhard explicitly identified social support as helpful during his adaptation after stroke. Bradshaw et al. (13) defined social support as a "network of family, friends, colleagues, and other acquaintances". Teilhard shared multiple instances where his wife, colleagues, and minister supported his continuity of identity, propelling him forward in his adaptation. First, he identified that his wife was very supportive without being overprotective. She allowed him to engage in "controlled adventuring" by protecting him from situations she knew would be too frustrating for him or from taking on too much. He acknowledged that, through negotiation, they decided together which challenges were safe to take in order to avoid "a huge disastrous downside ". She recognized his limitations and strove to set him up for success by providing the just right challenge, urging him to get out and do things. She helped him maintain his public persona by helping him "not to look shabby". Together, they chartered a thirty-eight and a half foot sailing boat and, with a captain, sailed the San Juan Islands. "Both my wife and I made the stroke a challenge ... and I can be innovative when challenged. I try to be resourceful in dealing with specific facets of my disability that I think I can circumvent. So I think it was a trade-off of resourcefulness for denial, which is a good trade any day. Other social supports were his colleagues. Teilhard shared one instance where a colleague was in town speaking at a	Family and social support supporting continuity of identity and adaptation<br><br>Spouse encouraging trying activities she belived he could achieve<br><br>Urged to take risks to identify limitations ad what could be achieved<br><br>Being realistic with expectations

	conference. Afterwards he stopped by to visit Teilhard at home and said: You really got to start ...behaving as though you're going to keep participating in research, create new knowledge, you've got to start going to meetings so... people will know that you're up and about and OK. This propelled Teilhard to begin looking forward to resuming his lecturing, teaching, and research. Teilhard was apprehensive about traveling with a disability, so he called his friend and colleague from a prominent medical school who had a disability and travelled in a wheelchair. He received advice about traveling and since then "has put over 175,000 miles on [his] cane". I did start trying to get myself on the program of international meetings and ...within the first year of the stroke I gave some talks in Germany that I travelled to alone ... and Tasmania. I have published book chapters and papers at an undiminished rate. The social support of his wife and colleagues, and his own determination, ultimately resulted in his resumption of worldwide lecturing.<br>Spirituality<br>Teilhardis spiritually active, and he finds purpose and meaning through pondering the details of life, particularly nature and natural processes. According to McColl (14), "disability evokes spiritual searching and reflection [and] it confronts people's own humanity and mortality, as it may arouse doubts in our assumptions about the essential goodness of the world". Teilhard experienced this spiritual soul searching as he was trying to make sense of the nature of God and His role in Teilhard's disability when he had the following experience: At the time I had the stroke ,our church ...[had]an interim minister.... She came and ...I was resting in the bedroom and she came in and sat down, no frills, and she said, 'Well ...are you pissed[off]?' ...and for some reason I really came unglued. I was just bawling. 'Cause I guess I was. This validation of feelings assisted him in moving forward through the grieving process. Spirituality is a complex phenomenon that has a variety of definitions. Johnston and Mayers (15) defined it as thoughts, feelings, and actions concerning the meaning we make of our daily lives. Yet, its influences can be felt in many ways, and one of those ways is through nature (14). After the conversation with the minister, Teilhard described a turning point at which he was finally able to make sense of his experience and it was nature that provided the impetus. I was driving up the street toward our house [during]springtime. A forsythia was in blossom ...and ... I was thinking theologically about ... the nature of God. Did God inflict this stroke on me? Is that the	Positive social support and encouragement to reclaim identity<br><br>Learning to cope from others positive experiences with disabilities<br><br>Spirituality as a means of escapism and searching for greater meaning<br><br>Validating feelings acknowledging them moving forward with grieving process positively

	kind of God there is? And then I said to myself, but still there is beauty [emphasis added]. And the fact that there's beauty says I think God is loving and wants to give us pleasure. And that was helpful. He noted that this spiritual realization anchored him and helped him to be at peace with God. Forsythia is a spring-flowering shrub prized for being tough and hardy as it thrives in less than ideal conditions and is the first to blossom in late winter and early spring. It was a fitting metaphor for Teilhard, as he was confronted with the challenging sequelae of his stroke. Teilhard took up photography after his stroke, and through taking photographs of flowers and other beautiful things he attempts to shares his perception of beauty, and therefore the nature of God, with others.<br>Internal locus of control<br>When the researchers asked Teilhard if he felt there were any positive consequences of having a stroke, he paused for several moments, stated he was taken back by the question and countered: I'm very reluctant to even hint that having a stroke is a good thing. I think that's self-delusion, and perhaps other people's delusion. Now, making the most of the situation ...which is just what I think we ought to be focusing on, is probably good for one in general. Internal locus of control is having the belief that one has a certain level of control over one's own life events (13). The belief that one can make the most of a given situation, even when it is not ideal, is closely tied to internal locus of control. Teilhard stated, "I was able to get back a lot of things that were important to me, in part by being a stubborn SOB, and two, being resourceful". For example, Teilhard particularly enjoys the process of developing his own film, and he figured out a modification that would substitute for his left hand to allow him to move the film to a developing reel in his darkroom. This enabled him to continue to participate in a self-defining occupation and foster his continuity of identity. He also learned to do a physical examination with one hand, and returned to clinical medicine. He figured out ways to successfully travel the world by being tenacious. "I can do most of what I need to do without my left hand.... I missed my left arm kind of like you miss one of your kids who goes away to college not doing something with you."<br>Building on past successes and commitment to succeed<br>Having past successful experiences in overcoming obstacles may help a person to develop the attitude that adversities are challenges (6,12). Teilhard exemplified this approach to life when	Spirituality and having faith in God as a means of coping<br><br>Adapting the environment around to be able to achieve desired activities

	he stated, “I can be innovative when challenged”, indicating that he has had success in dealing with past challenges such as overcoming atypical childhood circumstances of being raised by his grandmother and part-time father. In spite of the adversity of his childhood, he admitted that he was an ambitious and curious child, mastering individual sports and academic subjects. The combination of these personal characteristics led to academic and professional successes. His early successful experiences helped him to develop a strong internal locus of control and a strong commitment to succeed, another resilient characteristic (6). Teilhard used several words to describe himself including stubborn, tenacious, over-achieving, and “type A-ing” it. He describes himself this way both before and after his stroke, indicating his continuity of identity. In addition, it was this strong commitment to succeed that helped him progress through difficulties associated with his disability. About his return to clinical medicine, he said, “I think being a wounded healer was an advantage, because patients somehow found some kinship with me”. Above all, he did not want to fail, and this drove him to overcome any obstacle he encountered, whether through adaptation or increasing his physical abilities. He used the attitude “challenges bring out the best in you ”to work through the physical and emotional issues that presented themselves following his stroke, as he and his wife viewed the stroke and its effects as a challenge.<br>Action-oriented approach and personal goals<br>Teilhard exhibited an action-oriented approach and set goals for himself to return to his regular occupations as soon as possible. Teilhard had an ability to see what needed to be done in a given situation, identify solutions, and set goals for himself. This approach was very helpful to him in his recovery. He described himself as “resourceful”. One example of his resourcefulness was when he was told by his therapists that he had left neglect. He procured a computer from the hospital and began playing solitaire, a program in which the cards come off the deck in the upper left-hand corner. He used this activity to train himself to look and pay attention to his left side as he recognized early on that he had to address this issue in order to get back to other occupations, such as driving. While he was still on the rehabilitation unit, he had his secretary deliver his mail to him and resumed his work, even though it was premature. He went downhill skiing soon after his stroke with the help of his physical therapist; however ,here placed downhill skiing with cross-country skiing and snowshoeing, which accomplished a	Recognising successful experiences and using this to tackle new challenges<br><br>Proud to maintain this characteristic about himself post-stroke<br><br>Positive mentality and understanding how to adapt to situations<br><br>Self evaluation and task orientated goal setting<br><br>Adapting to a situation for the purpose to complete future goal

	similar experience of being in nature, something he values immensely. Likewise, he returned to traveling with his wife and guest lecturing around the world because of the goals he and his wife set for themselves. He also took up photography after his stroke, and later took up writing photo-poems. Because of his ability to identify and solve problems, the support of his wife, who facilitated his "controlled adventuring", and social support from friends, colleagues ,and his minister, Teilhard related, "I was really able to get back to a lot of things that were important to me".	Network of support aiding to success
15. Radcliffe, Lowton and Morgan, (2013)	Couples frequently began their narrative with a focus on the stroke event and then discussed in more detail living and coping with stroke in the present and near future. The interactional dynamics between the couples frequently shifted throughout the interview, with spouses often taking it in turns to narrate, sometimes talking between themselves while trying to negotiate their narrative, and at other times one spouse would take a more dominant role than the other. Couples' narrative style emerged as either predominately conflictual or collaborative, based on the frequency of the Veroff codes applied to each shift in speaker. The speech of the collaborative couples mainly built on each other's narratives, with a high degree of confirmation and confirmation-collaboration codes and very little conflictual interaction. The couples with a mainly conflictual style of interaction engaged in some collaborative interaction but also often disagreed or interrupted the spouse with a negative response and frequently continued talking without taking into account what their spouse had said previously (identified as continuation), indicating an implicit conflictual style. Non-response codes did not occur frequently. Hearing problems, common in older age, appeared to contribute to most of the non-response rather than this being intentional. These collaborative and conflictual styles of narrative underpinned the three types of copresentation identified in relation to the content of the narratives. These different forms of co-presentation are described below.<br>Co-presentation of identity<br>United couple: Presentation as a united couple was evident in aspects of most couples' narratives but emerged very clearly in six (Jack and Penny; Molly and David; Helen and John; Patrick and Betty; Gretel and Vas; and Tom and Nora). Although the stroke often initially caused great disruption to their lives, these couples presented themselves as morally competent, self-sufficient couples who had jointly adapted to varying levels of disability as a	

	result of the stroke. Their narratives showed that together they were currently managing life with a stroke as well as other illnesses and hardships, and often minimised the impact of the stroke. Their narrative style was largely collaborative, characterised by the frequent use of 'we' and by confirming and collaborating talk through repetition and overlapping to finish each other's sentences in ways that emphasised their partnership. One example is Molly and David. After Molly's stroke David had to help her with aspects of personal care such as getting dressed and he took on more of the household chores, despite his own heart condition and problems with his eyesight. However they presented themselves as a normal couple who engaged in activities as other retired couples did. As David observed, 'There a lot a people 'alf our age who're worse off than us, ain't they, you know what I mean', while Molly supported this adding that they were soon to go on a short holiday. To continue the storyline, each built on what the other had said, as illustrated by the following extract that focuses on David's past experience with the anticoagulant drug warfarin, a drug they both take.<br>M: We both go to the (warfarin) clinic together. D: The only trouble is it don't 'alf alter your blood you know, that warfarin.… I 'ad a lump come up didn't I, ah you wanna hear another problem? M: He had a lump in his jaw…. Never stopped bleeding. D: And eventually they can't stop the bleeding so I get them tablets, I got huh some acid tablets, they didn't stop it so James er from the er what was it? M: From the warfarin clinic. D: From the warfarin, he phoned up some professor geezer and he said er double it up but then we had another plan what we should have done in the first place, keep chewing on lumps of cloth, trying of course to stop the bleeding. I was waking up in the morning and my tongue was like that. M: Yeah it was horrible, wasn't it? D: Cough, oh my god, frighten you. I think we managed to sort it out ourself Molly didn't we [laughs]. (Molly, aged 76, stroke survivor and David, aged 81)<br>In some cases united couples described their relationship as having been strengthened through their shared experience of the stroke. For example, Gretal, a stroke survivor, described how she could go out of their high rise flat only if her husband (Vas) was with her due to her risk of falling. Rather than talking about being dependent on her husband she presented the two of them as 'a team' who were dependent on each other, demonstrating a sense of agency and autonomy as a couple. Throughout their narrative they engaged each other in a confirming and collaborating form of interaction, agreeing with one another and	Being grateful that they aren't as worse of as others<br><br>The united couple strengthen through shared experience to aid coping<br><br>Couples working as a team to facilitate autonomy as a couple

	building on each other's narrative: G: We have always been a loving and caring couple, I can say that without hesitation but obviously our relationship has changed because chores in the house I would do without thinking about it. I am restricted doing so … V: Now I've got to do it [laughs]. G: You don't have to, but you do it [laughs]. So our relationship has deepened. No? V: Oh absolutely. G: We are more, as I said before we have always been close, very close and we are well known among friends and family, they say with a couple like you, one doesn't fight very often … it was destiny that I came from Germany and Vas came from Cyprus, we met and lived happily, happily ever after … Both of us we are a team. We've always been but since I had the stroke – V: We've always been, now it's more, because she needs me. We are more close than ever. (Gretel, aged 78, stroke survivor and Vas, aged 80)<br>There were variations among co-presentations as a united couple. Tony and Cathy's narrative differed markedly in structure to that of the others as, although Tony had made a good physical recovery from his stroke, his aphasia meant he spoke in a slow, stilted manner and had some difficulty finding the correct word. As the narrative progressed Cathy began to take a more dominant role by prompting, correcting or answering for Tony and finishing his sentences. Tony would also ask Cathy for confirmation or support to continue the storyline, shown here in the context of his recounting the history of his stroke:<br>T: I, we was on the boat, hh and um, no I had heart attack on the boat and I got to New York and they took me off the boat into the hospital and they fitted me with a pacemaker and it's, how many days after C: It was about 4 days after you had a pacemaker put in, he had the first stroke… T: Affart from the s, speech, um, that's all the result of the stroke, uh I don't C: Yeah I mean obviously you're not as agile now as what you was before. T: Huh now and again I get, um, leg, leg, left leg seelsa bit funny at times but ur I get about on that. C: Not your left leg, your right leg. T: Uh right leg. C: Because it was all down the right side. T: Right leg. C: where it was affected, yeah, yeah. T: Sometimes it feels a bit funny but apart from that. A, a all er the troubles that I uh had is the breathing problems. C: 'e has, he's made a fantastic recovery. You know, as I say, no one thought he was going to come through it, even the doctors was so surprised. (Tony, stroke survivor, aged 75 and Cathy, aged 74)<br>Together the couple sought to minimise the impact of Tony's aphasia by using humour, enabling them to display an image of a normal united couple. For example, Cathy joked about	Pride in relationship as an identity amongst friends<br><br>Dependence on partner and stroke has brought relationship closer together<br><br>Partner aiding in confirmation of knowledge

	her own hearing problems saying, ‘he can’t get his words out and I can’t hear, we’re a pair well matched!’ Although Cathy assumed a dominant role in the narrative she appeared to use this to construct a socially acceptable presentation as a united couple, with Tony actively collaborating and inviting Cathy to take this role through his requests for confirmation from her at different points throughout the narrative. This could therefore be regarded as forming ‘scene support’, rather than Shakespeare’s notion of ‘scene stealing’ by a dominant individual in the dyad (Shakespeare 1993). This narrative form did not occur in the co-constructed narratives of the other two participants with aphasia, possibly because they did not experience problems with word-finding as Tony did, as well as reflecting differences in individual personality.<br>Carer relationships:<br>‘positive’ and ‘frustrated’ carers All couples brought up caring for their spouse following stroke as an unprompted part of their narrative. However, in some narratives the notion of a spouse as carer was an image that dominated the couples’ co-presentation. This is illustrated by Jack and Penny, who together engaged in a confirming-collaborative narrative to present themselves as a selfreliant couple, with Penny willingly caring for Jack who had very limited mobility and required the use of a walking frame and wheelchair. Together they presented Penny as a positive carer, with Jack describing how well he was looked after and Penny emphasising that it would be difficult to seek assistance from a paid carer due to Jack’s stubbornness, implying that she was the best person to care for him. This was supported by Jack who emphasised Penny’s ‘natural’ role as his carer:<br>J: Penny’s be, been brilliant… In hospital I didn’t like them cleaning me and washing me …If the wife’s doing it, I mean, we’ve been together alotta of years and it seems more of a natural thing. But to have a stranger doing it, it’s, to me that weren’t on … The thing is I’ve always been a very, very stubborn person. P: Oh yes, very stubborn.… Well I know basically how, what he likes, what he don’t like, you know, and I’m here all the time. J: She knows how moany I can be. P: Oh yes, I don’t think a carer could take it, you know. They’d be running away [laughs]. J: She lays the law down … but really when I sort of think about it, I, she’s right. (Jack, stroke survivor, aged 79, and Penny, aged 59)	Using humour as a means of coping and unity<br><br>Self appointed roles within the family<br><br>Establishing roles of carer and patient rather than husband and wife<br><br>Trust and comfort with partner being carer instead of a stranger<br><br>Understanding the needs of husband more than a stranger would

	Whereas this extract accords with the notion of women as being 'natural' carers, it was not only wives who were presented as positive carers, as illustrated by Grace and Simon's narrative:<br>G: Not one of my children would ever do me, open their hearts to me as how Simon… He wash, he cook, he iron, he clean, he do everything believe me… He's not well as well so I don't want to see him lay down 'coz if he lay down I will suffer. S: … I'm not too well really … I've got this prostate [problem] … life have to continue.<br>G: He have to do everything, he have to put my clothes on for me. So it is really bad. Really, he, he wash me. So I am really bad. I'm not hiding it and I can't, God give me him for a reason.<br>(Grace, stroke survivor, aged 78 and Simon, aged 75)<br>Other spouses similarly talked about their caring responsibilities with a sense of pride. For example, Betty related that her general practitioner told her that by nursing her husband 'in the right and proper way' when he had pneumonia recently she had 'saved the hospital hundreds of pounds'. Similarly, Judy described herself as a good nurse and reported that while visiting Richard in hospital after his stroke a nurse told her that she admired the way Judy cared for her husband. However whereas Judy described her positive caring role, this was accompanied by aspects of a carer who was dutiful but 'frustrated', as shown by their relationship and style of interaction. This notion of a frustrated carer also characterised the account given by Rita. Judy and Rita were both very keen to tell their own story of their husband's stroke in terms of the disruption it meant to their own lives due to the high level of care their husbands needed and also both husbands' severely limited eyesight, which had led them to be registered as blind. This is illustrated by the following extract in which Judy engaged in a conflictual style of narrative, interrupting her husband and seeking to present herself as the dominant narrator who will tell the 'correct' version of the story:<br>J: He used to go down to the pub every day, you know, to have a couple of pints and he just come barging in one day and through the door and he said, 'I can't walk'. And he just got near the chair and I had to grab 'im, he just fell to the floor nearly, and I just got him in a chair. So I called our doctor and she came out. She was a French doctor at the time, and er she said, 'Oh, you've had a slight stroke Mr Finch and she' – R: She sat there, says, 'you're having a stroke' J: Yeah, yes, well we know that Richard. I'm just giving her [the researcher], I'm telling her	and able to motivate because of this<br><br>Without partner they would not cope they are reliant on them completely<br><br>Family having pride within their new responsibilities<br><br>Frustration from family due to level of acquired responsibility of care

	exactly what happened. [Addressing the researcher] Sorry, I'm not being rude to him, but he does this every time. (Richard, aged 83, stroke survivor and Judy, aged 67)<br>Similarly Rita who presented herself as a dutiful but 'frustrated' carer took a dominant role in her narrative with Ted:<br>Rita: I've had 'eart attack myself, well I've had three stents put in as well you see so, obviously I can't, when he falls I can't pick him up. And I've also got arthritis all over me back and I just can't whatsaname so I can't cope with him … I have to cut his food up for 'im and 'cause, you know, he can't cut his food up properly and he can't see what's on the plate and that goes everywhere … He can't see the steps when he goes out like, you know. When he goes out he's got his stick, his white stick and that but I was out one afternoon, he'd gone across the shops on his own, you see so he can't be trusted. Ted: I've gone across to the shop on the crossing. R: That doesn't make any difference, whether you're on the crossing or not because you can't see … I tell him he's not to answer the door when I'm not here. So but I think on the whole his life has completely changed. (Ted, stroke survivor, aged 84 and Rita, aged 83)<br>A further variant of the carer relationship was provided by Sarah and Nelson. This narrative focused on Sarah's role in caring for Nelson 'day and night' during their 60 years of marriage in view of his depression and mental health problems, and Nelson's inability to act as a carer for Sarah, who was left severely disabled and housebound after her stroke. The couple talked about Nelson's severe depression happening as a direct result of Sarah's stroke and how he spent 8 months in a mental health unit from the week she was discharged from the stroke unit, meaning they were living apart during that time. Although Sarah had slurred speech she took the dominant role, talking about how she coped with disability and how she relied on paid carers and her children. Their narrative differed from those of Ted and Rita and Richard and Judy as the conflictual interaction was less explicit with minimal response from Nelson.<br>Underlying tension and conflict between Ted and Rita, Richard and Judy and Sarah and Nelson emerged in the structure of their narratives, largely dominated by themes of illness, profound disability and the need for a high level of care. Various health problems experienced by both the stroke survivor and their spouse appeared to be making their lives very difficult. The couples' conflictual narratives therefore appeared to be at least partly driven by the practical realities and hardships they were experiencing in coping with stroke and other ill health at the	Spouses undermining partner who has had the stroke<br><br>Frustration from partner and lack of trust for stroke survivor to be safe alone<br><br>Stroke of one partner causing adverse health concerns for other partner<br><br>Outcome: Both partners having health concerns makes QOL difficult

	time of the interview and may have been influenced by the relative recency of the stroke, with interviews at 1–3 years post stroke.	
16. Satink et al., (2016)	Results and discussion Betsy: ‘‘I need him, but without him I could do more’’<br>Betsy, 67 years old, and cohabiting with her spouse Jan, expressed that she had mixed feelings about the way Jan supported her after her stroke. During various encounters with Betsy, she repeatedly expressed to the researcher that she needed her spouse, that she was glad for his support in different situations and how he organized the household in general. However, she also said that he was too careful with her and in some ways even limited her. Two situations will be further described with possible explanations.<br>‘‘I need him, but he always cooks the same!’’<br>During the first encounter, Betsy was sitting with Jan at the dinner table preparing a shopping list. She had planned to go to the supermarket and the researcher (TS) was scheduled to join her. She stood up and put her coat on, and put her bag, wallet and keys in her walker, then she walked with the researcher to the supermarket around the corner. Outdoors Betsy said, ‘‘You know, I welcome his help, but he can only cook three meals. It is often the same’’. Betsy expressed to the researcher that she had always been the one who did the cooking and that she really wanted to cook again. Once in the supermarket, Betsy collected all groceries from the shopping list. She could find everything, looked at the prices and decided what to buy. She also took a few other vegetables and mentioned that she always had done so: ‘‘I often take extra vegetables. It means that you can choose, and create different meals every day. A little wok is always delicious’’. When Betsy came back from the supermarket, she gave the bag to her partner. After Jan responded surprised that she had bought too many vegetables, she said, ‘‘But I can help you with cooking! Maybe we can use the wok’’. One possible interpretation is that Betsy explored new possibilities and tried to negotiate with her spouse about the possibility of cooking together. Based on the past, where she was always the one who cooked, and based on positive experiences in other activities she had done recently, she might have created a future storyline where she saw herself cooking again. Knowing that past and future influences present actions,[43,47] she might have bought extra vegetables to explore and negotiate the possibility of doing something she did before the stroke: cooking a meal. Almost two years post-stroke, Betsy was not cooking anymore and rarely went to the supermarket.	Recognising the importance of family for support<br><br>Being overprotected by spouse preventing progress of self<br><br>Roles changing within the family dynamic<br><br>Attempting to negotiate role within the family to regain pre stroke identity

	During the last visit, she told the researcher that Jan was often not amused when she came home with more groceries than he had written on the shopping list. At the time of the visit, Jan was doing most of the shopping. In contrast, Jan said the new situation had been overwhelming for him and that he had lost his ''pal'' at home. He expressed how difficult it was to cope with the changes at home, and he did not exactly know where to start to solve the challenges in the household himself. On one hand, he was devoted to assist Betsy in everyday activities, but on the other hand, he explained, he could often do these activities easier and quicker himself. Jan perceived this as the best way to manage himself: ''I also have to take care of my own time. Activities like grocery shopping, cooking, or household activities I can do much faster than her. Beside these activities, I also have other things to do''. Jan said they had stopped talking about it with each other and that they both tried the best they could in their new situations. From the perspective of comanagement and role management,[11,58] one possible interpretation of the situations described previously is that each person tried to manage the situation in her or his own way, but both were taking care of their own daily business without a dialogue with each other. Both persons might have had different understandings and expectations of their everyday activities and roles, but there was no dialogue about their perceptions of the changed situation and roles post-stroke. A dialogue between Betsy and Jan about the meaning of activities and finding a new balance in role management could have been a way to negotiate capabilities and expectations about future roles and the best ways to perform activities. Moreover, a dialogue could have helped them to tune in to each other instead of maintaining the current gap between them, and it could support both persons in doing the everyday activities they really wanted.[59,60] Concerning the cooking, Jan argued that it was best for both that he prepared everything, and that, at the end, Betsy could sit in the kitchen and check the potatoes and vegetables when they were boiling. Jan explained his internal conflict: he wanted to help Betsy, but he also wanted to prevent any mistakes she might make, as he experienced sadness when Betsy struggled in activities. Jan said, ''I sometimes have to leave the kitchen because I cannot stay close to Betsy and face her struggles during household activities''. To prevent that Betsy would struggle during household activities, he took over everything. If he felt too frustrated or sad, he sometimes left the kitchen and asked Betsy to do some easy activities herself. However, the	Lack of home support not open to negotiate changing roles<br><br>due to ability to effectively undertake activities<br><br>Partner not being equipped to manage change<br>Lack of communication between family members being a barrier to change<br><br>Hindering progress by helping and over cautious

	activities that Betsy was asked to do had no meaning for her. She explained, ‘‘Jan proposed that if he first prepares everything and puts it on the gas, I could sit in the kitchen beside the gas cooker and control the potatoes when they were boiling. But well, I can better read a newspaper. I stopped cooking’’. The consequence was that Betsy was not engaged in cooking anymore. The steps of the cooking that she was allowed to do were too simple and meaningless for her. Furthermore, it also resulted in a situation where Jan wished to see a ‘‘happy Betsy’’ who could do simple activities without mistakes, while Betsy said she was actually less happy because of his way of supporting her. She said she had accepted her mistakes while doing everyday activities. She just wanted to cook. Jan argued that he was critical regarding the way Betsy performed activities. He said he thought that the best way to help her was to give feedback about the failures and to structure or take over the activities when she was trying out everyday activities. The stories and actions of both Betsy and Jan can be interpreted in different ways. Based on Vik et al. [37] and Kessler, Dubouloz, Urbanowski and Egan,[61] who described the importance of developing a sense of agency and building competences in participation in meaningful occupation in the years following stroke, one possible interpretation is that a vicious cycle started. We imagined how Jan, Betsy’s spouse, did not stimulate her to perform different activities or to relearn by doing. As a result, her sense of agency decreased. If we link this to the process of role management, Betsy is an active agent in the beginning of the post-stroke process where she did the shopping and cooking. Initially, she negotiated with Jan to do more everyday activities step by step. However, during the last encounters, Betsy was a more passive agent who was not creating new situations or activities. She rarely participated in meaningful activities like cooking. Consequently, she was not able to redevelop her competences in cooking in order to regain this important role. ‘‘I need him, but without him I could do more.’’<br>Almost a year after the stroke, Betsy said that she’d had a very good day when Jan was away for a full day with friends. She said, ‘‘We had made a list with different household activities that I could do. I was really happy at the end of the day. I had done all the household activities by myself, in my own way and own pace.’’ When Jan had returned home, she had shown him what she had achieved at home. Betsy later told the researcher how she wanted to share her success of completing the household activities by herself and had hoped that she could do this	Not being able to completed meaningful activities of daily living<br><br>Lack of understanding between partners regarding desires and expectation<br><br>Being an active agent to facilitate change and role<br><br>Being a passive agent and loosing desire to develop more abilities<br><br>Success by goal setting and completing meaningful activities

	again, but Jan had responded stoically to her achievement. Ricoeur [43,44] described that, when performing everyday activities, people interpret their experiences and produce images that are connected to past or future events. By doing this, people create possible storylines or scenarios that are used in their negotiations about future situations. Related to this, role management and Betsy's different actions as her own agent can then be seen as a negotiation between Betsy and Jan about future activities and each other's roles. Moreover, role management was also Betsy's internal dialogue about her future scenario, her ''self'', and her identity and about the roles she had and which roles she was able to regain.[35,62,63] In this case, she might have interpreted her recent experiences as not only being able to do the household activities, but also as a possibility to negotiate with her spouse about doing more activities in the future.[43,44] Betsy was also engaged in activities outside her home in the community. She repeatedly talked about her visits to an African family to coach them in the Dutch language and culture and her time at the local activity centre where she helped to serve lunch once each week. She explained, ''The people at the activity centre take the time for me. I know that it costs them time, but they tell me when things are not going as it should go. I know that I'm not so quick. I can only serve two meals per time with my walker, but that is okay for them''. Betsy said that she really liked to go there, that she had accepted that she made little mistakes, but she could let it go. Helping other people in the activity centre was more important for her. In general, we have interpreted how cooking and household activities, even when these went differently before the stroke, were possibilities for Betsy to reconnect to her important pre-stroke roles. Betsy had accepted that trial and error were part of the process of self-management. For her, the experience of mastery in activities was more linked to the meaning of an activity and the satisfaction of the ''self'' [64] (chapter 6). Cooking and household chores were her pre-stroke roles, and starting to do them again was a way to regain these roles as well as a sense of self and identity.[2,35,63] Betsy's self-management was embedded in the management of the ''self'', roles and identity, which we have interpreted as role management.[11,24]<br>Martin: ''I can still orchestrate my life.''<br>Orchestrating the people around him to reconnect to roles	Eliciting desires to regain sense of identity and self thus trying<br><br>Creating an identity and motivating self to claim this<br><br>Public acceptance for limitations thus aiding motivation to continue<br><br>Success was achieved by completing meaningful activities related to identity

	Martin was sitting in his wheelchair during the first encounter. He told the researcher that even after his retirement he had been working as an ambulance nurse and community nurse. He shared pictures of the time he had worked as an instructor in the army before the stroke and showed how he still could track the ambulances on his computer when they had to go to an accident. Martin talked mainly about work-related issues and not about his stroke. His wife said during the first encounter that work was ‘‘his everything’’. When the researcher asked Martin 21 months later about the biggest changes post-stroke, he responded immediately, ‘‘Not being able to work again. That is the biggest change!’’ However, in the various encounters, Martin often told how he had visited some people in the flat he knew from the time he worked as a community nurse. He said, ‘‘Of course, I was not visiting them as a nurse, but I can still have a talk with them’’. He had also become a member of the residents’ association and had recently organized an event for the residents of the flat where his old ambulance mates had given a presentation about first aid for elderly. One possible interpretation of Martin’s story is that he tried to reconnect through these activities with his prestroke role as nurse.[8,65] Although he was not a professional nurse, being with others and talking and giving advice to others was still meaningful for him. Martin tried to reconnect with this through new activities in and around his building in order to reconstruct his sense of self and continuity of his roles.[65] When he performed these activities, he might have created possible future storylines; [43,47] these storylines could have helped him in the negotiation with and reinvention of his social self, his roles and his identity.[62,63] During the third encounter, Martin wanted to go to the park to take the dog out for a walk. His wife, Annie, helped him with his jacket and put the dog on a leash. Once in the garage of the flat, Martin made the transfer from his wheelchair to the mobility scooter, and Annie connected the dog leash to the mobility scooter. Martin and the researcher went to the park and a conversation began about the possibilities for him to go out independently. Martin said he had to get used to the fact that his wife had to help him with dressing and washing, but that it felt normal now. Later on, in the conversation, he expressed that he needed her and that she was really important for him because Annie’s help made it possible for him to go out to see his friends or visit people in the flat. For Annie, it was normal to do this. She said, ‘‘In the period that I was ill, he helped me so much. It is normal to do this for him now’’. She also said Martin had not	Identity base on previous work self pre stroke being important so attempting to reclaim this<br><br>Keeping social relationship with people who knew him as the identity he wants to be<br><br>Having a sense of purpose and meaning<br><br>Accepting support and limitations<br><br>Being an active agent by using what he has and people around him

	changed after the stroke: ‘‘He is still orchestrating the people around him. He has people for all kinds of activities and knows how he can approach them’’. An example was given in another encounter where Martin told how he had asked a friend for help because he wanted to create a flower box on the balcony. Martin had phoned his friend, and his friend bought pots, sand and flowers for the flower box. Martin showed the researcher his balcony and how he could water the little plants. He said, ‘‘You know, I cannot sit the whole day inside my house. I want to go out or sit on the balcony’’. From an agentic perspective, we have interpreted Martin in these situations as an active agent. He was the one who had the lead, but was able to ask other persons for assistance when he needed it. He used the capacity of others in order to control and manage the situation with others, which can be viewed as proxyagency.[33,42] Role management for Martin was to organize people around him to help him and fulfil his role. Role management was situated and coconstructed by Martin through everyday activities, which is referred to in other studies as comanagement.[11,58] Moreover, role management for Martin meant considering different scenarios with regard to future roles and possible adjustments he had to make. Another situation where Martin organized services in order to be able to go out and fulfil his social roles was when he made a phone call with someone from the municipality during the first encounter. Martin explained on the phone that his current mobility scooter was not good enough to drive into the hilly area of the city where he lived and that he could not go out with his dog or meet other people. He expressed to the researcher that arranging things like phoning people from official bodies and arranging services for himself was not difficult for him. His wife said later, ‘‘That is typically Martin. He knows how to do this. He has always done this!’’ One possible interpretation is that Martin was an active agent and a good medical manager, as he arranged the care and resources in order to deal with the consequences of the stroke.[8,32] We have interpreted this as an action of a role manager, meaning that Martin managed his surrounding situations in order to be able to perform his social roles.[8,32] Having a better mobility scooter would mean that he could go out with his dog and play his desired role in his social network. We saw a person who was able to negotiate with people to create situations as he wanted them to be. Moreover, Martin’s wife trusted him and gave him space to explore possibilities. Although Martin was dependent	to facilitate coping and to achieve<br><br>Understanding limitations and using what they have<br>Remaining in control of future<br><br>Planning as a means of coping<br><br>Being an active agent and a role manager of self-destiny

	on his wife and other persons, he was in charge. He was orchestrating the people around him in order to reconnect to and fulfil the roles as he wanted to fulfil the roles.<br>''There is still a life with only one hand.''<br>In various encounters, Martin often reflected on everyday activities. He tested future possibilities with his wife and the researcher. One example is how Martin talked about his physiotherapist. He often said that she was a good professional who challenged him, knew how to handle him and trusted him. On a certain moment, almost one year after the stroke, Martin talked about the moment he had walked a few metres with the physiotherapist. He explained how good it felt to take the first steps, and how it gave him hope about his possibilities for the future. He said, ''Well you know, if I can walk again, I might also be able to drive a car again, go to the shop myself, or visit people outside my own village''. One possible interpretation here is that the experiences he had and the meaning he gave to the first steps with his physiotherapist were much more than a step with a walking aid. From a narrative perspective, where the past and present can shape future storylines,[43,47] Martin could have created different scenarios around the possibilities of walking again or even driving a car. Through Martin's positive experiences of relative mastery while walking,[64] he gained access to the world of ''as if'',[43] which enabled him to continue stories such as walking and driving a car. Driving a car would not just be ''driving a car'', it would open up new possibilities and allow him to regain old roles. A similar interpretation can made about the following situation: Martin, who had just gotten his driver's license back, had just bought an adapted car, and he and Annie had been driving in the city. When the researcher had coffee with the two of them, Annie said, ''He drives safer than me!'' A bit later, Martin started to joke about travelling by car to a fisher's village the next weekend. He referred to the delicious fish they had always bought there in the past. Suddenly, he said, ''And if we go to the fishers village, we might also go to [our son] Jeroen'' His wife laughed and said, ''Well, if you have something on your mind, it will happen''. One possible interpretation of this situation is that the positive experience of driving the car in the city had opened up the idea of going to a fisher's village and buying some fish. It was something they had done in the past, and the idea and possibility had come alive again. Martin said that he always got his first ideas when he was doing activities. We interpreted that, through the experience of relative mastery in everyday activities, Martin created images of	Attempting and trying new things to understand what could and couldn't be done<br>Trust in health care professionals to challenge and aid recovery<br><br>Hope created though experience of achievement and success<br>Achieving gain closer access to previous roles<br><br>Having goals to achieve facilitating change

	future activities and ways to fulfil roles. He tested the possibilities of future activity and negotiated with his wife. Through the activities, either by doing or by talking about them, Martin tested new scenarios. He talked about his dreams and considered possibilities of how to manage, continue and reconnect to old roles. We interpreted Martin as an agent who had sufficient confidence to take action, to negotiate with others and to manage his old and new roles. Although he had limitations, he was an agent with the ability to create meaning in life post-stroke. As he said, ''there is also a life with only one hand''.	Comanagement between partners to problem solve together
17. Shannon, Forster and Hawkins, (2016)	Initial questioning about problems revealed that the meaning of a 'problem' varied between individuals and issues, and some rejected the word 'problem', therefore, it has been termed as 'issues'. A broad range of issues were reported while some were described as impairments (e.g. limb weakness, fatigue, difficulty remembering) and others were depicted as the impact of impairment (e.g. on social, domestic or leisure activities). These issues were understood as being related to stroke, comorbidities or ageing, and impacted on their daily lives causing some frustration due to limited or inability to carry out tasks or activities. One participant identified a potential future need (moving home) and others indicated changeable scenarios (partner becoming too unwell to continue providing support, icy weather reducing mobility). Other than contact with their Family Doctor, participants were not receiving formal health or rehabilitation support at the time of interview. However, all participants received some form of support from others, and often relied on this. Primarily this was from their partner if they had one (nine participants), and/or help from others including family, friend or neighbour (six participants), charity (three participants), or paid services (two participants). Support from charities included providing information, emotional support, and services for small domestic repairs. Paid services included use of a gardener and a cleaner. Support could be informational (e.g. knowledge about stroke), emotional (e.g. offering conversation) and practical (e.g. personal care, shopping, cleaning). In addition to talking about support, a minority of participants also discussed a number of practical strategies they had developed for managing regular difficulties, such as taking their time, keeping things close to hand or using specific tools or everyday objects as an aid. All participants interviewed reported living with current impairments or limitations; and were thus not 'problem free'. Despite this, participants did not report having unmet need. Four main themes were identified, which help inform the self-	Support network, having to rely on someone<br><br>Support from charities on information regarding coping<br>Pacing strategies as a means of coping<br>Adapting the environment around to manage coping

	reporting of low or no unmet needs on the LUNS questionnaire: level of acceptance, relative situation, presentation of character, and expectations and experiences of services.<br>Level of acceptance<br>Most participants described acceptance of the issues identified through a process of adjustment, coming to terms with their health and life changes, or a focus on things they can do (rather than those they cannot). They were also able to provide a positive interpretation of the situation, such as talking about not having to worry, overall contentment, the ability to maintain a good quality of life, noting improvements they had experienced since their stroke, and referring to those they are hopeful for in the future. Participants could also actively engage with their difficulties, for example, acknowledging and facing up to things which have changed, or talking about doing what you can and accepting things you cannot do:<br>I still want to do the things that I did previously, I don't find them as easy to do, but if I can do it I will do it, even if it takes me longer, which it very often does [laughs] .... I just have to accept it, if I can't do it, well I've just got to accept it ...I think learning to accept what you can't do is the main thing. (Joan).<br>Gradual changes prior to stroke (rather than sudden change) were sometimes noted, such as retirement, transient ischaemic attacks, other health changes or ageing. Because their health and lives were already changing prior to their stroke, people had begun to adjust their expectations, daily lives and perception of what might be considered a problem or unmet need:<br>You've got to sort of face up to the fact that you're not going to just manage things as easily .... My life's changed obviously, I mean, life has changed totally but I think I'm adjusting. (Pauline)<br>Two participants did not demonstrate the high level of acceptance seen in the majority of the sample. Both had experienced significant changes in roles, routines and activities important to them, perceived these as a negative interruption, and were focussed on returning to pre-stroke activities. When asked what effect the changes had on James' life, he replied:<br>The way I feel, drastic. It really had, it's, it's just ruined my life not being able to get out and do me gardens and that.<br>Relative situation	Recognising acceptance influencing adjustments and to what they can achieve rather than what they cannot for better QOL<br>Keeping track of improvements recognising hope<br>Understanding limitations and moving forward<br>Pacing and Humour as a means of coping<br><br>Accepting that change will occur gradually<br><br>Barrier to acceptance is inability to undertake pre-stroke activities

	Participants commonly viewed their lives and issues relative to other people and other circumstances. Being fortunate was reported, that is, feeling contented about how things are compared to how they could be. For example, good stroke recovery, being financially comfortable, and having family nearby. Participants made comparisons to other people, other times, and other outcomes, for example, in the case below, Joan compares her situation to a poorer outcome:<br>Everybody says I've done very well, and I really do feel I have, and I'm very pleased about that, could have been a lot worse off ... I've been very fortunate, it hasn't affected my speech or it hasn't affected my memory or anything like that, so I've been very lucky really.<br>Participants received information from peers and family, and this appears to shape expectations about their own lives. By talking with others, they ascertain what is likely to happen to them, what difficulties are normal for someone at their age, or for someone who has had a stroke. Participants gather and use information to reflect on their own situation.<br>Considering his own life in the context of others who have had strokes made George feel better about his own situation, and influenced his response on the questionnaire identifying unmet need:<br>I've been to the Stroke Club, and you see people with arms that don't work down there in different forms and some can't speak properly, so I was pleased with that, so that's why I ticked things on the form.<br>Presentation of character<br>Some participants presented their character in a positive or active light, as a way of explaining how they manage their daily lives and difficulties. Determination, pride, and/or independence were noted. Determination at achieving post-stroke recovery and being an active 'doer' was reported, referred to by Charles' wife as his ''sheer guts and determination''. Another interviewee, Pauline reported:<br>I think you've got to have the willpower. It would be easy I think to sit back and say 'Oh I can't do this, I can't do that, I can't do the other', you've got to be willing to meet it, if you know what I mean, you've got to be willing to meet these things and do what you can.<br>Pride was identified when talking about good poststroke recovery:	support network<br>No financial burden aided mental health<br><br>Comparing self to others and being grateful and pleased with situation- good recovery<br><br>Coping through shared experience<br>Understanding what is normal and how to cope<br><br>Same as light blue above<br><br>Having a positive attitude and great work ethic for trying things<br><br>Having pride in progress

	I’ve always been able, since I’ve come back from hospital, to walk upright and keep my own balance .... Dave next door is a professor, and he says from time to time ‘’He is a miracle’’. (Charles).<br>Independence (being independent from the help of others) was reported by some who did not want to seek or receive help:<br>I’m not bothered about anybody helping me. I want to do it on my own. (James).<br>The desire to present themselves as having pride, independence or determination helps to explain how participants manage their identified issues, and why no or low unmet need is therefore reported.<br>Expectations and experiences of services<br>Expectations and experiences of services appear important to participants in explaining whether they perceive their issues as unmet needs. Three elements of this were un-treatability, rationing and current contact with services. Un-treatability describes participant perception that their issue is unlikely to be helped by contacting services and an inability to envisage what help could be offered. For example, David received Speech and Language Therapy but still has communication and memory impairment, he therefore tends to think that further help may not be effective, and this influences his responses when asked whether he would like help:<br>The speech lady came, didn’t she? ... She had a nice sense of humour and all this. And I liked her but she didn’t help. I was sorry when she stopped coming but ... she didn’t help, and I thought all these, ‘would you like help with this and that and the other’ ...I couldn’t do it, she couldn’t help, though she wanted to very much and I wanted to be helped but I could tell it wasn’t going to do it for me.<br>Participants also described an awareness of limited resources or rationing, frequently perceiving that people other than themselves will be more in need. When reaching the end of community physiotherapy and making the decision whether or not to continue, two participants questioned what more the therapists could do to help, considered that the issue might be untreatable, and knew that other people may benefit more:<br>Much as I would have liked for them to carry on, I had to accept that they had other patients that were more of a priority than me, so we accepted that they weren’t coming. (Charles)	Trying harder to regain identity<br><br>Barrier in Believing nothing will help so do not try<br><br>Trying not to be a burden on services as a barrier to therapy

	Perceptions of un-treatability or rationing illustrate that participants are unsure whether pursuing treatment is worthwhile or justified, perhaps making them less likely to report unmet needs. Many participants were currently in touch with services and were content with this method of managing their issues, resulting in some needs being met. Family Doctors appeared to be the first point of contact, with participants recently having visited, or intending to visit, their Family Doctor about an identified issue:<br>The thing is that this has only just started has this pain since the Physios stopped coming, and I've arranged to go and see my doctor on Thursday to talk about it. (Brian)<br>In contrast, one participant was concerned that he had received no follow up from a stroke specialist since discharge from hospital and felt his Family Doctor did not understand his stroke-related impairments. Despite this, he intended to continue visiting his Family Doctor, perhaps because this is the only option he has.	Dependence on healthcare practitioners to facilitate recovery
18. Young et al., (2013)	It was evident in the discourse with each patient that fatigue was a salient concern. After several readings, often requiring recoding of the patient statements, a consistent coding (subthemes) was determined by the interviewer. These were subsequently confirmed by 2 of the authors, and the results were then organized by agreement into 6 themes (see Figure 1). The first 4 themes related to the experience of fatigue and its psychological impact. The final 2 themes related more to the experience of dealing with fatigue (see Table 1).<br>Theme 1: Tiredness/sleep The overwhelming experience of all patients was tiredness. This was a prominent theme amalgamating a number of similarly coded subthemes, reflecting analogous ideas expressed in differing ways. Exhaustion (subtheme): "I feel exhausted, even after sleep … feel raving tired"; "I feel tired, I get worn out" (patient 6). These feelings included mental as well as physical tiredness: "I feel too tired, I just can't really concentrate…I get worn out" (patient 8); "You're trying to work it so much but it's [the brain] just not doing anything" (patient 9). All patients reported that fatigue had caused them to need more sleep and rest and it was clear that patients felt they spent considerably more time resting, in bed, and sleeping, than they had before their stroke. Sleep (subtheme): "Whenever I have a second I go to sleep" (patient 1); "I've got to have sleep… I didn't sleep much at all before, now I can sleep… much longer" (patient 2); "I've got to have a sleep" (patient 6); "I'm always asleep, always in bed" (patient 5). Tiredness (subtheme): Sometimes patients reported that they fell	Post-stroke fatigue playing a role as a barrier towards recovery

	asleep despite their best effort not to do so and that such sleep may or may not be refreshing: "I still feel tired, even after sleep" (patient 1).<br>Theme 2: Restriction Several of the patients reported that fatigue restricted them in various ways, even sometimes in basic self-care like washing and dressing. The main theme emerged from a number of initial subtheme codes. Limitations on activities (subtheme): Patients felt that because of their fatigue they could do nothing they considered worthwhile or things they wanted to do. One patient for example, noted, "You can't talk to people for long" (patient 9).<br>Theme 3: Frustration Although patients described frustration in varied ways, it was apparent that all patients were frustrated by their feelings of fatigue. Subthemes coded from the transcripts were as follows: Poor coping (subtheme): Patients found it hard to accept their condition and the change in their perception of self. They found it "hard to accept myself … I need to give myself a slap" (patient 1). Boredom (subtheme): In some circumstance the frustration was derived from the fatigue directly: "There's just nothing to do … Yeah I'm frustrated" (patient 1). Lack of control (subtheme): Sometimes this was directly expressed with emotional emphasis: "I find it frustrating to be dependent on my partner" (patient 2); "Even simple things are difficult to do… it's a constant effort" (patient 3); "You're trying to work at it so much but it's just not doing anything… it makes me feel annoyed and I start thinking can I get better" (patient 9).<br>Theme 4: Depression/motivation Many of the patients expressed signs of depression, feeling "down" or helplessness. In a few cases, the depression or low mood was pervasive and present for a large part of their time and, in that regard, qualified as clinical case status. Mood swing (subthemes): In the minority of patients, the mood state varied: "sometimes upset," "fed-up" (patient 1); "I resent it" (patient 6); "have become more moody and I can snap like that" (patient 9). Poor motivation (subtheme): One patient suggested that he slept not only because of fatigue but as "an escape…if I could fall asleep I could forget what condition I was in" (patient 3); "feel less like doing things really" (patient 8); "I generally don't feel like going if I have to wait" (patient 7). Doing nothing (subtheme): "couldn't be bothered" (patient 1). Depression (subtheme): One patient stated, "Depression is a big thing, yes it's affected everyone that's had a stroke. It has to, otherwise they're lying" (patient 2). Sometimes the	Fatigue being a barrier towards completing meaningful activities<br><br>Poor coping due to problems accepting limitations<br>Boredom causing frustration<br>Frustration due to reliance on family members – lack of control<br>Failure causing frustration and lack of belief<br>Mental state and depression affecting motivation and mood<br><br>Lack of motivation and wiliness to do things as a barrier

	emphasis was on helplessness rather than mood per se: "It's a feeling you can't do anything" (patient 4).<br>Theme 5: Determination/coping Despite their feeling of fatigue, many patients felt a determination not to give in to such feelings. They wanted to feel independent, to push themselves when feeling fatigued, and to exert control and get things done.<br>Achievement (subtheme): several patients engaged in behaviours that provided a positive attitude; they felt a sense of achievement from such activities: "I always try to do what I can within reason …I enjoy the challenge" (patient 6). Goal setting (subtheme): This was often related to the previous subtheme, but the objectives were more specific: "I'll try to set goals, because that's what I'm like" (patient 10). Control (subtheme): This was reflected in statements such as, "I push myself to go out…I won't give in to it. I'm quite determined that if I want something, there's a way around it; I never really give up" (patient 9). The majority of patients had found some way to manage their fatigue: "It helps me cope better if I have something to look forward to in the afternoon" (patient 7).<br>Theme 6: Support In some form, all patients referred to support. Sometimes it related to professional services and other times to the wider social context of partners, family, or friends. Informal support took varied forms, such as visiting patients, taking them on trips, providing meals, even sharing hobbies. This may have led to direct tangible support, but patients also recognized that it distracted them from the consequences of stroke, including their fatigue. This provided enjoyment and an antidote to negative emotions. Patients had attended physiotherapy and occupational therapy sessions, and these improved physical symptoms and provided alternative ways of doing things. In many cases, support helped provide a sense of achievement. Mood (subtheme): Visits to and from friends could reduce negative mood states – "felt less bored and down" (patient 7) – and improve positive mood status – "felt refreshed" (patient 4). Advice (subtheme): This was frequently from professional services: "It's helping me… and you feel as if you have achieved something…rather than by going for something huge and getting even more frustrated because it can't be fi nished" (patient 1). Saturation in the subthemes was reached by patient 8. No new subthemes were added, although the experiences of each patient remained unique.	Coping by pushing self to achieve and gain control<br>Positive characteristics to willingness<br><br>Trying to take back control by goal setting and trying<br>Making plans to give meaning and purpose to trying<br><br>Distraction as a means of coping<br>Sense of achievement form healthcare assistance to motivate recovery<br>Maintaining social identity and social connections improved mood<br>Advice to understand what is normal and what has been achieve and can be

Mind mapping –

**Meaningful Activities of daily living**	**Social Identity**	**Awareness of progress**	**Level of independence**
Achieving activities which are purposeful	Identified by what they can achieve	Acceptance	Gained independence by doing thing by themselves
Physical recovery defined successful living	Achieving a sense of purpose	Recognising doing nothing will cause regression	Ability to mobilise somewhere (travel of functionally)
Adapting the world around them	Role within the family (negative and positive)	Progression is gradual process (positive recipient/ frustration)	Overcoming barriers
Completing/ adapting old activities	Communication	Where they were compared to where they are now	motivated/ hardworking/ positive "I Can" attitude facilitating recovery
Risk taking	Meta-perception. Ways in which other see them (disabled/ disabling self by thinking this)	Denial and uncertainty	Family/ support systems reducing independence
Goal setting/ Achieving milestones (good and if unrealistic =bad)	Work identity	Realising limitations and capabilities, finding a balance of can and cannot	Unsupported achievement of activities of daily living
Pride in doing/ purpose	Recognising no longer old self but instead a new identity	Loss of hope due to inability	
Recognising success then tackling new challenges	Barrier due to comparing old self to new self	Change in behaviour – to see the world differently	
Developing routine and forward planning	Meaningful social relationships	Due to what is achieved with Therapists	
	Isolation/ social inclusion	Self-reflection – rethinking priorities	
		Awareness of condition – taking control	

Thematic Development

Theme	Subtheme	Code	Unit
**Meaningful Activities of daily living**	Achieving activities which are purposeful	Definition:<br>As survivors reengaged in meaningful activities, their experience of quality of life changed over time (1)<br>“Be involved with everything.” “Have a hobby.” “Live as much as you can; do as much as you can.” These participant statements illustrate clearly one predominant theme that emerged from the data: the central role of “doing things” as a core component of living successfully (2)<br>“Doing little was described as counteractive to living” – activity has to have a purpose (2)<br>Time spent on hobbies or leisure activities offered participants time for relaxation or solitude: “Time for myself (referring to photo of meditation CDs)”, “This is releasation [relaxation] (describing photo of dart-board)”; enjoyment: “It’s fun. It’s fun”; and for maintaining a positive mood: “Makes me happy (discussing photo of gardening)”, “Keep you—spirits up—yeah.” (2)<br>Books were important for her ongoing sense of recovery and quality of life (8)<br>in terms of participation, the amount of participation in activities was less crucial than the degree to which participation was achieved with a sense of worth. (11)<br>For example, Teilhard particularly enjoys the process of developing his own film, and he figured out a modification that would substitute for his left hand to allow him to move the film to a developing reel in his darkroom. (14)<br>Because of his ability to identify and solve problems, the support of his wife, who facilitated his “controlled adventuring”, and social support from friends, colleagues ,and his minister, Teilhard related, “I was really able to get back to a lot of things that were important to me”. (14)<br>However, the activities that Betsy was asked to do had no meaning for her. She explained, ‘‘Jan proposed that if he first prepares everything and puts it on the gas, I could sit in the kitchen beside the gas cooker and control the potatoes	

		when they were boiling. But well, I can better read a newspaper. I stopped cooking'' (16)	
	Physical recovery defined successful living	Definition:<br>Her experience of recovery as she regained physical skills enabled her sense of "human-ness" to remerge. As activities were resumed or replaced, survivors also regained a sense of their self-confidence, competence, personal influence, and meaning. (1)<br>: "I am so tired, the fatigue is getting better, I am no spring chick any longer … really tired me out visiting family." Feelings of unhappiness resulted from the lack of ability to participate in family gatherings (4)<br>They also described limited options in terms of places they could visit, for example, not being able to go further than the local shop. (4)<br>There is a constant tension in my leg, so it is also very swollen and sometimes I cannot really be anywhere because I simply feel pain in the entire arm and the entire leg. (6)<br>We find that Mette constructs her ABI primarily at a physical level. (6)<br>Men in this study measured their recovery by the extent of physical recovery attained (7)<br>Paul's confidence with swimming, or Wilfred's growing confidence in using his left hand.(8)<br>"[laugh] Start with this [gestures everything][laughs]".<br>The view that aphasia adversely affects all aspects of life was reported by the participants without aphasia, a kind of "fragmentation" in opposition to the movement towards "wholeness" (11)<br>Patients had attended physiotherapy and occupational therapy sessions, and these improved physical symptoms and provided alternative ways of doing things. (18)	
	Adapting the world around them	Definition:<br>Finally, a number of other participants discussed how they had adapted by trying something completely new and different: "I have to [change my] ideas of life—	

		I'll have to do something else, instead of what—I was a truck driver . . . that's out. Do something with myself . . . like forestry . . . camp[ing]"; "I wouldn't have thought about floristry until after my stroke . . . I was . . .working in the office before my stroke (referring to photo showing a flower arrangement)." (2)<br>Aphasia - adapted their environments to assist understanding—for example, captions on the TV, or "big books [large print books](description of photo taken at local library)". (2)<br>Reduced energy levels, tiring easily during group meetings and struggling to follow group conversations resulted in a preference for meeting friends one-to-one for some participants (4)<br>The two themes of participation and sense of worth were integral to the consideration of QOL in terms of adjustment processes. (11)<br>Because of the uncertainties John experienced, he decided to move to a single-storey apartment (12)<br>Well, in shops with a lift, I take the lift. Because people are used to walking on the right-hand side of the stairs. I can use my right hand, but it drags a lot. So I really have to try and get my arm up onto the railing and raise my leg as well, which is very difficult. (12)<br>. She also said Martin had not changed after the stroke: ''He is still orchestrating the people around him. He has people for all kinds of activities and knows how he can approach them'' (16)<br>discussed a number of practical strategies they had developed for managing regular difficulties, such as taking their time, keeping things close to hand or using specific tools or everyday objects as an aid (17)	
	Completing/ adapting old activities	Definition:<br>"You didn't think about it before you had the stroke, but, now, after the stroke, it means a lot to be able to do the things you could do before."(1)<br>Participants described continuing activities from before their stroke, adapting old activities to enable continued participation, and finding new interests and activities. (2)	

		"I'm back to doing everything I did before. I guess it feeds into sustaining your hope." Personal progress was a crucial factor in sustaining hope. (3)<br>Still being able to participate in previously enjoyed activities, even if done differently, and to feel valued, (5)<br>One man remarked " I will not be getting any better" and noted that instead of looking for changes "I am learning to adapt (7)<br>He procured a computer from the hospital and began playing solitaire. He used this activity to train himself to look and pay attention to his left side as he recognized early on that he had to address this issue in order to get back to other occupations, such as driving (14)<br>Talking about doing what you can and accepting things you cannot do:<br>I still want to do the things that I did previously, I don't find them as easy to do, but if I can do it I will do it, even if it takes me longer, which it very often does [laughs] .... I just have to accept it, if I can't do it, well I've just got to accept it ...I think learning to accept what you can't do is the main thing (17)	
	Risk taking	Definition:<br>Tring new things and do it," one participant suggested. Another said, "I have to take risks so I can get further on" (2)<br>The adjustment processes therefore concerned increasing independence (linked with the personal processes of increasing confidence and self-determination), trying "new things", and ultimately modifying environments. (11)<br>". She recognized his limitations and strove to set him up for success by providing the just right challenge, urging him to get out and do things. (14)<br>After Jan responded surprised that she had bought too many vegetables, she said, ''But I can help you with cooking! Maybe we can use the wok''. (16)	
	Goal setting/ Achieving milestones (good and if unrealistic =bad)	Definition:<br>A real sense of achievement was evident for participants in describing these milestones: "I got the job . . . I accomplished something that I didn't think I'd be able to do"; "That's an award (referring to photo of a certificate) I got for . . . volunteer[ing] . . . I do my best—quite [an] achievement . . . (laughs)." (2)	

		will set a goal in hospital and it may be ''I'd like to get independently to the hairdresser'', (10)<br>So every, it started at the beginning and I said, right, I'm going to encourage you every day to be what you can be.... day by day, like we set wee goals. When he came out, we set goals. (10)<br>it's difficult when you are living with someone to see any real progress. So I would write down each week things she was doing and compare that with previous weeks and it's amazing actually what the difference there actually is (10)<br>I get them to use a calendar, get them to tick the days they exercise, so in the week, they become focused because some of them are kind of housebound (10)<br>priorities in their mobility practices, to achieve satisfactory bodily, family, working, and community lives, to let go of particular pre-stroke pleasures, and to reformulate goals every day and everywhere, every time and again. (12)<br>Teilhard had an ability to see what needed to be done in a given situation, identify solutions, and set goals for himself. (14)	
	Pride in doing/ purpose	Definition:<br>. One participant described a self-portrait taken for the photo interview: "There's me. And I am a success with aphasia." (2)<br>She was proud about her achievements in the Art exhibition (Interview 3: "a little bit heaven"), and hopeful at being "a little bit better"(Interview3)and" along time yet ...each time a bit more"(Interview4). (11)<br>This [picture] also impresses me. Interviewer: Why does this photo impress you? Lastri: Because I can help my children by finding firewood. It means I can already make an effort (13)<br>Teilhard used several words to describe himself including stubborn, tenacious, over-achieving, and "type A-ing" it. He describes himself this way both before and after his stroke, indicating his continuity of identity (14)	

		Relationship- G: We are more, as I said before we have always been close, very close and we are well known among friends and family, they say with a couple like you, one doesn't fight very often (15)<br>Pride was identified when talking about good poststroke recovery:<br>I've always been able, since I've come back from hospital, to walk upright and keep my own balance .... Dave next door is a professor, and he says from time to time ''He is a miracle''. (Charles). (17)	
	Recognising success then tackling new challenges	Definition:<br>It's getting confident to do things. I find the first time you do it, you are a wee bit apprehensive, but then you've done it. You've achieved it and the feeling is brilliant, so you don't think twice about doing it again. (10)<br>Moreover, the participants identified benefits in "getting around" impairment, such as setting compensatory goals in the "What Next?" groups, and practising new strategies in both "safe" environments and with others with aphasia (peer support and sheltered courses). (11)<br>Martin often reflected on everyday activities. He tested future possibilities with his wife and the researcher (16)<br>activities: "I always try to do what I can within reason ...I enjoy the challenge" (patient 6). (18)	
	Developing routine and forward planning	Definition:<br>Many wife-caregivers also expressed a loss of independence and autonomy, as they restructured their daily routines to accommodate the presence of the husband.<br>"I find he stays home more, and this is stressful because it changes my routine." (7)<br>After sometime, the changes in marital life became routine. Couples did not reflect much on the past; rather, they wanted to look forward (9)<br>Ben explained that although he did go out, he sometimes struggled if something unexpected happened, such as having to go to the second floor of a shop without a lift (12)	

		. Many of our participants avoided public places, or when they had to go out, they planned carefully how, when and with whom they would go (12)<br>She also had to plan almost all her activities in advance, such as shopping. (12)<br>When I go shopping I always prepare a shopping list the day before. I know exactly where everything is in the shop. So I write my list based on the route I take through the shop. (12)<br>The majority of patients had found some way to manage their fatigue: "It helps me cope better if I have something to look forward to in the afternoon" (patient 7). (18)	
**Social Identity**	Identified by what they can achieve	Definition:<br>Karen felt influential in her new job: "I think I've made a positive contribution to the company as a whole."(5)<br>Through personal, social and environmental aspects. This concept included a sense of self (knowing and being known) and a sense of purpose (where activity had personal, religious or community meaning (11)<br>Ramelan, for example, had been well known in the community for his ability to call people to prayer (adzan). His stroke had initially rendered him too weak to continue this role, which had clear social consequences, (13)<br>For her, the experience of mastery in activities was more linked to the meaning of an activity and the satisfaction of the "self" [64] (chapter 6). Cooking and household chores were her pre-stroke roles, and starting to do them again was a way to regain these roles as well as a sense of self and identity	
	Achieving a sense of purpose	Definition:<br>." Mimi was not just saying that caring for her family makes her happy, although she stated that it is "the greatest joy" she has. She was making the point that caregiving makes her feel healthy, capable, and accomplished. Her quality of life is enhanced" (1)<br>Definition:<br>Social activities to help others.	

		"Oh yeah and of course you are not thinking of [yourself]... you are not pitying yourself. You are out there helping." (3)<br>My son Is a little spoiled, but my only job now is to be a good mother and that has to be done well of course I felt that I had abandoned him [he was 8 years old then] (9)<br>I'm lucky I've got children and they want a mum so you've just got to get on with it.... (10)<br>Well...one of the psychological things in my brain, was I need to be, get better for my wife and my family 'cause they need me...I want to see my grandchildren growing up, you know that was a real driving force for me [Male Survivor, 65 years old] (10)<br>Faisyal had previously run an informal group for religious education. After he had made some recovery from his stroke he had chosen to restart this as a priority, albeit on a smaller scale than previously and with support of his wife: It's very meaningful to me; it's a service that I could give to people. And it also is a communication between me and people around my life. (13)<br>She repeatedly talked about her visits to an African family to coach them in the Dutch language and culture and her time at the local activity centre where she helped to serve lunch once each week (16)<br>Although he was not a professional nurse, being with others and talking and giving advice to others was still meaningful for him. (16)	
	Role within the family (negative and positive)	Definition:<br><br>A photo of her putting on make-up: "I'm learning how . . . want to have my face done . . . very important. Because he's [my husband's] good. But he's not a woman. So I want to do myself. Very important." (2)<br>. An example is Emily, living with her husband and child. Prior to the stroke, she was the one mainly responsible for her 3-year-old's upbringing. Post stroke, her husband became the main carer. "Up until now, he [her son] was truly a mama's boy [...] Now [husband] is the playmate, the reader, the explain it all person." (4)	

		. Andrew felt he had lost his role as a man and a husband within the family, and was worried his wife might leave:“Well, it certainly changes things in terms of your role within the family because certainly now I’m dependent now [rather] than a partner. “What sort of a husband am I?” It does worry. I do sometimes think to myself, “What’s my wife getting out of this relationship and how long are we going to be able to sustain it?” (5)<br>She repeatedly presented herself as a capable, committed single mother who was striving to cope simultaneously with her impairment and motherhood. (5)<br>“It is really, really big psychologically.” Becoming a mother and having an ABI was especially very difficult: Well, anxiety, bad conscience, bad conscience and bad conscience. (6)<br>the stroke has made me useless. I cant do things for my family like protect them. If an intruder came in I couldn’t even defend my family”. (7)<br>This is not only about me; it [the stroke] was destructive for my children My daughter moved to another county when she was only 16. I think she wanted to be far away think it was best for her [Rita, stroke survivor]. (9)<br>… If I was healthy I would get some money for the family … I have to be able to walk [to work (13)<br>She used all her seven selected photographs to discuss her relief that her capacity to be independent in some familial roles now facilitated her children’s independence (13)	
	Communication	Definition:<br>Humour - Participants commented: “And I laugh. I just laugh”; “You can sort of take funny—funny sides of it . . . make a joke out of it”; “But laugh. You’ve got to laugh.”<br>Striving for a positive (2)<br>Humour – One of the frustrating things about aphasia for me is the lost opportunities to make snide remarks. I’m too slow … I couldn’t say it (4)<br>communication acted as a significant barrier to living successfully with aphasia (2)	

		Humour - previous role as the quick-wit and raconteur, even though his physical and language impairments were, on the surface, relatively mild.<br>for example reading “newspapers” and “books”; being able to give a “talk” or “speech” at a church or community group; writing a signature “so that I can go shopping” or “at work . . . sending emails”. In many cases participants reported restrictions in being able to carry out activities because of communication difficulties (2)<br>“Now I can . . . talk . . . That’s a very important thing . . . Being able to talk is the main thing”; (2)<br>example Beth, whose aphasia was initially severe, avoided friends as she felt uncomfortable socialising: “I was embarrassed by my speech [...] I hid from friends while healing”. (4)<br>The worst thing is that I cannot have a conversation and discuss things with him. it is difficult so we turn on the TV. Aphasia is the worst thing! (9)<br>. Jan said they had stopped talking about it with each other and that they both tried the best they could in their new situations. From the perspective of comanagement and role management,one possible interpretation of the situations described previously is that each person tried to manage the situation in her or his own way, but both were taking care of their own daily business without a dialogue with each other. (16)	
	Meta-perception. Ways in which other see them (disabled/ disabling self by thinking this)	Definition:<br>Monday afternoon I went to see a second grandson play tennis...and as I approached every eye is on me, and here I am walking with a limp, and have to be helped to my seat, and uh, and suddenly I just have the feeling that all of them said, “look at that man who is cripple, who is lame and cannot do for himself,” and then I start crying. (1)<br>Jess described her difficulty participating in a game due to fatigue, leading others to assume she was not intelligent which she found upsetting: “And the FRUSTRATING thing is that is that people think it’s because you have a low IQ, which isn’t the case (4)	

		R: Hm. PWA2: And they say's "you're drunk". R: Ohh. PWA2: And I says "I'm not drunk" and it was a Saturday afternoon, Saturday 2 o'clock afternoon. R: Hmm. PWA2: I says "I'm not drunk", you know, you get people come in here at 2 o'clock in the morning [unintelligible] you know and em, I got me stroke card out and they says "I don't care if you've had a stroke" and I got me aphasia card out as well [gestures putting card down on table]. (11)	
	Work identity	Definition:<br>"Doing things for one participant, highlighted the role of her work activities in her self-identity: "that's important because that's what I do . . . pertinent to me." (2)<br>For some, loss of work also entailed loss of the social contact which went with the work environment. New and positive social interactions post stroke (4)<br>A selfemployed farmer expressed his concerns about his ability to return to farming if his persistent difficulties with his walking/ hand and arm strength did not improve. (7)<br>"I want to work, to be perfectly honest with you but I'm kind of (.)I'm just getting through the summer …. (11)<br>Agus, a married father of two children below the age of four years, had been a cart vendor prior to his stroke as well as tending to crops, jobs he could no longer do "… I, of course, cannot work." (13)<br>Martin talked mainly about work-related issues and not about his stroke. His wife said during the first encounter that work was ''his everything''. When the researcher asked Martin 21 months later about the biggest changes post-stroke, he responded immediately, ''Not being able to work again. That is the biggest change (16)	
	Recognising no longer old self but instead a new identity	Definition:<br>I'm trying to . . . get as close as I [can to what I was]—before the stroke . . . I know that that's not possible, but . . . as good as I can get and I'm happy." (2)	

		. It's as if I had one life and it ended when I had the stroke, and I now have a completely new life. . . I've had to claw my way back to health as I knew it before. (5)<br>"What do we do, to uphold the quality of life that we had before? Then we just have to compensate." (6)<br>I am entering a transitional period, where I will have to learn to get over my past perceptions of self-image. Perhaps it is time to focus on other things. (7)<br>I am in a transitional period… the arrogance that I had before, that I was fit and masculine and therefore immune to disease is gone now. Better QoL and has taught me the importance of moderation and balance. (7)<br>PWA3:"Hmhm. Well you can't, it's different altogether. You know, you feel a different person altogether [gesture to self]". (11)<br>Moreover, the task of "Looking back, around and forward" was facilitated by an integrated consideration of all aspects of life), and to envisage these aspects together when looking forward was part of reforging links between the different aspects of self and the reworking of self-image. (11)<br>While she was unable to return to previous role, she had a number of children who had, over time, taken on the task. This allowed her to spend more time on other household jobs which she was able to manage (13)	
	Barrier due to comparing old self to new self	Definition:<br>Whereas before, you know when getting up, you know and you get ready, take a shower and you get gone. And that's what I miss. Um, that was good. That since it's now, and just here, and that's it. There's nothing … everything is the same nothing (1)<br>"There's more to me than what you see now. I used to get out, could outrun anyone, most of the average person, I played ball and I was a typical teenager, and I was such a vibrant person.". (1)<br>They all had extreme difficulties adjusting to their losses, as illustrated by Jenny's description of the impact on her and her husband's life:	

		"Both of us have found the effect of the stroke absolutely devastating because we were, you know, we were starting to do things. Like we'd started going on walking and dancing holidays and we were starting to do things like that that we found really, really enjoyable, and I suddenly can't do them anymore." (5)<br>You see, I am the social Mette, who has always been "the clown," who got totally wasted at the disco, right. [...] And now, I cannot even consider having one drink (6)<br>Loss. Referred to elements of their lives, physical emotional or social that had been lost or altered by the minor stroke event. "it's a struggle to accept the fact that im not the person I used to be, that I can't contribute like I used to" (7)<br>Frustration for PWA2 concerned his inability to work as a head chef, difficulty getting words out (Interview1), and at fatigue after talking (Interview 2). (11)<br>Looking back" comprised reminiscence and reflection including reassessments of past life, remembering the recovery sequence in relation to the length of time since the stroke, and in relation to loss.(11)<br>The way I feel, drastic. It really had, it's, it's just ruined my life not being able to get out and do me gardens and that. (17)	
	Meaningful social relationships	Definition:<br>Factors in their lives were seen as contributing to living successfully with aphasia (e.g., "The most important thing is my partner"). (2)<br>Meaningful relationships: "It's really people." A second theme identified by participants as central to living successfully with aphasia was meaningful relationships. As one participant stated: "I think it's really people . . . that make you feel successful." (2)<br>. Friends were referred to with great appreciation: "They are my rock and my true north" (4)<br>"He [her husband] fell in love with a Mette who is not there anymore. And how does that affect his love for me?" Mette preferably wants to position herself in a way that would help her maintain her social and personal relations (6)	

		relationships built up over time were viewed as the key to overcoming isolation rather than simply "getting out (of the house)", (11)<br>However, in the various encounters, Martin often told how he had visited some people in the flat he knew from the time he worked as a community nurse. He said, ''Of course, I was not visiting them as a nurse, but I can still have a talk with them''. (16)<br>Visits to and from friends could reduce negative mood states – "felt less bored and down" (18)	
	Isolation/ social exclusion	Definition:<br>Due to communication for example, "talk[ing] on the telephone . . . [to] old friends . . . that's very important" or "I don't write to Mum . . . being able to write letters would be [good]." In particular, the strong impact of communication on the loss of friendships was evident, with one participant commenting on the loss of conversation with friends, "Even if it's friends coming here. You don't know how to (indicates to mouth) . . . they're coming and say hello [but not the same conversation]." (2)<br>The changes in social interaction caused by the aphasia led many participants to feel they had lost a part of their former selves. They could feel unsociable, anxious and inclined to avoid crowds (4)<br>. Rather than go out and engage in his former hobbies, Nick described how post stroke he preferred to spend time alone. Environmental factors, such as using public transport and bathrooms and eating and drinking in public, were also perceived as barriers (4)<br>about meeting the maternity group, and it has been difficult for some (of the other mothers from this group) to understand, that I did not go out with them. So it has been really hard going from being very, very social to living in a box. (6)<br>"In contrast, others felt there were no options for follow-up. Yvonne thought that if she rang to request more therapy for John then she would be refused and she was not prepared to accept group work as an alternative:"|I just feel she would say ''no'' (8)	

		He cannot start a conversation; sometimes, when we have visitors, he leaves the room He refuses to visit our friends So it is he who withdraws, not them [Charlotte, spouse]. (9)<br>You do lose quite a few friends because you can't do everything independently any more (12)	
**Awareness of progress**	Acceptance	Definition:<br>– Acceptance: "I've got to accept it." For many participants acceptance was also a component of striving for a positive way of life. (for example, driving: "I can't drive . . . To me that's a big negative. [But] It's not negative thinking. It's . . . accepting I suppose"); (2)<br>Moving ahead, getting on with and enjoying life: "Look forward." Following on from acceptance, participants also described a mind-set of "moving ahead," "getting on with life (2)"I am happy as can be. There is nothing more that I can do so I put up and enjoy life.""I guess I just accepted it, because you cannot do anything about it, I guess I had to accept it." (3)<br>This thing has happened to me. . . . I just want to get on with my life and . . . I can't turn the clock back. I've got what I've got, go and deal with it and run my life accordingly. (5)<br>"Oh you've got to realize," she said, "your age and you see, you were very fortunate up to now. You've never had any serious illness up to now and plenty of people had." "Well," I said, "that's true enough," I said, "I can't grumble." I didn't have, and at my age I've been very fortunate. It's the only way to look at it. (Helen) (5)<br>Sense of self. Gradually began to restore balance in their lives. This new balance was achieved by an appreciation and acceptance of what had transpired in their lives. Current reality (7)<br>The progression included a variety of positive outcomes (themes of increased independence, enjoyment, hope, looking to the future and aspirations, openness and understanding (of others), finding a sense of worth, and crusade) coinciding with increasing acceptance. (11)	

		, 'There a lot a people 'alf our age who're worse off than us, ain't they, you know what I mean' (15)<br>Martin said he had to get used to the fact that his wife had to help him with dressing and washing, but that it felt normal now. (16)<br>Most participants described acceptance of the issues identified through a process of adjustment, (17)<br>Everybody says I've done very well, and I really do feel I have, and I'm very pleased about that, could have been a lot worse off ... I've been very fortunate (17)	
	Recognising doing nothing will cause regression	Definition:<br>"The worse thing they could do . . . You sit at home in a house . . . watch TV—no no. That's not right (laughs)"; "If there's most people around that say, oh I'll do nothing. They're going downhill" (2)<br>"[Hope has to be the backbone.] It has to be, because if you have a negative attitude you are not going to get better. You are just going to dwell on your problems and they will get worse." (3)	
	Progression is gradual process (positive recipient/ frustration)	Definition:<br>. Nina was aware of her limitations but was equally hopeful that her limitations would not last. As she was able to resume doll making, the presumption was that her quality of life would be affected. In her mind, she was not stuck at one place in time but rather was working along an ever-changing continuum toward a better quality of life. (1)<br>I think you've just got to . . . take every day after the next one. . . Sometimes . . . you take three forward and then you drop back two. But then you sort of pick it up again. So it is . . . an ongoing problem." (2)<br>The women expressed the importance of taking it day by day and being happy with any progress they were making.<br>"No, I just went day to day and did what I was told."<br>"I just take every day as it comes and I make the best of every day." (3)	

		"what is going to happen with my life," and "what will happen to me, and what will happen to us." In using these words, she accepts and actually takes on a more passive position of waiting and being in a standby position - a passive recipient (6)<br>Realization. There was also awareness about the potential long-term impact of the minor stroke at an individual and couple level. Recognition and adaptation would be a continuous process, perseverance and resiliency would be necessary in ensuring the family would remain intact. (7)<br>James had also invested a lot of effort in therapy and found it helpful to a degree. But by the time I spoke to him, over 2 years since his stroke, his frustration and anger existed alongside a resignation that he could no longer exert control over his recovery: (8)<br>Participants, all with chronic aphasia, still hoped for improvement in their aphasia and talked of "getting better" despite little or no change in their aphasia severity during the intervention period (11)<br>"everything is changing all the time". (11)<br>You've got to sort of face up to the fact that you're not going to just manage things as easily .... My life's changed obviously, I mean, life has changed totally but I think I'm adjusting. (Pauline) (17)	
	Where they were compared to where they are now	Definition:<br>"Just gradually changing and gradually getting better." (2)<br>"When I had my stroke—two years [ago]—I can see how far I've come . . . a gauge . . . I had no voice and couldn't speak. Two years—I can talk properly"; "I feel quite . . . successful from where it started [(2)<br>By reflecting on the beginning stages post stroke and progress they had made, participants were hopeful for continued recovery in the future. Some described the importance of small improvements. (3)<br>There's things I can't do, I'll never be able to do again, I know that. But I look upon it, I was lying in my bed after my stroke, the list of things that I could not do was a great big list and the list of things I could still do was very, very small.	

		So over the last year, I've transferred things over from "can't do" to "can do," and the lists are beginning to settle up. (5)<br>noting improvements they had experienced since their stroke, and referring to those they are hopeful for in the future. (17)	
	Denial and uncertainty	Definition:<br>"I can't be successful . . . Doesn't matter which . . . [person with aphasia] you're speaking to. They can never be successful . . . Impossible. Nobody can . . . It will always be there in the back of their mind" (2)<br>However, by constructing such a hope of going back, she also constructs a possibility of being the same and, thereby, being part of the same social environment that she once belonged to. (6)<br>I: Okay, is there anything in this short rehabilitation […] that has given you any new positive experiences?<br>M: No. […]. But it is also difficult, because you see, I am not affected that affected physically. It is really, really big psychologically.<br>I: But because you are not affected physically, that much-<br>M: Then they could not really help me. (6)<br>Michael, a 69-year-old farmer, described his prognosis as ''unbeknown'' (8)<br>She was 61 years old and felt that she was too young to be seen somewhere for therapy which was associated with more elderly and disabled people. (8)<br>Most references to anger included the term "frustration", the term used in the grief model, and the theme off ear was very closely linked with sub-themes about uncertainty (especially about the future) and vulnerability (11)<br>. For example, denial was manifested as disengagement, withdrawal or avoidance as a means of coping with the fear of social contact (11)<br>Denial was also manifested as over-preoccupation, rigidity or misdirected activity (which may be aligned with anger) to avoid positive adjustment (11)<br>Patients found it hard to accept their condition and the change in their perception of self. They found it "hard to accept myself … I need to give myself a slap" (18)	

	Realising limitations and capabilities, finding a balance of can and cannot	Definition:<br>I can't do whatever I used to do. And I would—I feel that I'm useless . . . [But] I'm not depressed and . . . I laugh . . . And I am finding that I am living successfully with the stroke. Yes . . . I go for a walk. I ride the bike (indicates to exercise bike in lounge) . . . go out shopping with my wife. And go for an overseas trip. And I feel alright—yes. (2)<br>Acceptance of impairments, looking for new occupations or interests when old ones could no longer be sustained, and/or finding new ways of doing tasks. "So I try to see that I still contribute. I do not go to so many meetings. I only go to the meetings where I could contribute something. I don't go just because my attendance has been ticked." (5)<br>"I had a belief that I am cured. . . . I'm better now. I could say one hundred percent better now." His considerable recovery, his limited day-to-day activities, and the fact that he had lived with the impacts of stroke for 7 years could explain why he felt recovered despite continuing limitations (5)<br>I just don't have energy; I just don't seem to have any. I don't have the inclination either you know. Aye, yes, I am fine lying in my bed the plans I have and what I am going to do and what I'm not going to do and it never materialises. [Male Survivor, 83 years old] (10)<br>. Mike was able to adjust his pace and the way he tackled the stairs, depending on the situation, which shows considerable flexibility. (12)<br>She had to think consciously about every push on the pedals to prevent herself from falling. Despite this, she developed strategies that enabled her to enjoy cycling again:<br>Mary: I don't take the time to look around and think, hey this is lovely … No, you think gosh I'm tired or I have to sit for a bit. … Then I find a bench and I go and sit down.(12)<br>creative in dealing with the ambivalences and tensions they face in regaining mobility in home and community life. It is this process of endless tinkering,	

		weighing, adjusting, and coordinating mobility practices in each situation anew, that needs to be acknowledged by the rehabilitation team (12)<br>She allowed him to engage in “controlled adventuring” by protecting him from situations she knew would be too frustrating for him or from taking on too much (14)<br>So I think it was a trade-off of resourcefulness for denial, which is a good trade any day. (14)<br>. He was the one who had the lead, but was able to ask other persons for assistance when he needed it. He used the capacity of others in order to control and manage the situation with others, which can be viewed as proxyagency (16)<br>coming to terms with their health and life changes, or a focus on things they can do (rather than those they cannot).(17)	
	Loss of hope due to inability, loss of motivation – do nothing	Definition:<br><br>: “Because I can’t get out there and do what I want to do. So—just got to stay here to do nothing.” (2)<br>“You lose hope because you can’t get better. You know you can’t. So it goes by the board.” (3)<br>“If you don’t have hope, there is no life.”<br>“If you don’t have hope you have nothing, it just gets hold of you and that’s it. You wouldn’t have anything to fight for.<br>“What would you do without hope? It’s a thing that you live with.” (3)<br>Yes, I thought I could go to the gym and surely I would get better there. ‘‘No’’. So I then bought me a Wii...I couldn’t do it and I did it all wrong and I went and hurt myself on it. I hurt my back. So that was the end of the Wii.... I can’t do it (10)<br>You get certain individuals that are completely motivated and will go to any length to get to where they want to go and that’s great, and then others, it’s maybe their motivation slips and they just see barriers (10)<br>This frequently resulted in a tension between accepting impairment and maintaining hope (11)	

		The paving stones are hopeless, you soon discover that if you can't walk properly, all paved paths are crooked. That means that I go out less often than I could. (12)<br>But as the months passed and no functionally significant improvement was made, this energy, time commitment and financial investment was reduced (13)<br>Melati: I cannot work, no, I cannot, I cannot even cook anymore. (13)<br>The speech lady came, didn't she? ... She had a nice sense of humour and all this. And I liked her but she didn't help. I was sorry when she stopped coming but ... she didn't help, and I thought all these, 'would you like help with this and that and the other' ...I couldn't do it, she couldn't help, though she wanted to very much and I wanted to be helped but I could tell it wasn't going to do it for me (17)<br>Limitations on activities (subtheme): Patients felt that because of their fatigue they could do nothing they considered worthwhile or things they wanted to do<br>"I feel exhausted, even after sleep … feel raving tired"; "I feel tired, I get worn out" (patient 6). (18)<br>Even simple things are difficult to do… it's a constant effort" (patient 3); "You're trying to work at it so much but it's just not doing anything… it makes me feel annoyed and I start thinking can I get better (18)<br>); "feel less like doing things really" (patient 8); "I generally don't feel like going if I have to wait" (patient 7). Doing nothing (subtheme): "couldn't be bothered (18)	
	Change in behaviour – to see the world differently	Definition:<br>"I would sit out there on the patio…and I would look at the air and the trees and the and the birds and the sky, whereas before I was too busy to do all this" (1)<br>"I have a better attitude towards myself and other people, and um, you know, for that I'm thankful. I guess I just pay attention to more details and the quality of things" (1)	

		), "disability evokes spiritual searching and reflection [and] it confronts people's own humanity and mortality, as it may arouse doubts in our assumptions about the essential goodness of the world". (14)<br>A forsythia was in blossom ...and ... I was thinking theologically about ... the nature of God. Did God inflict this stroke on me? Is that the kind of God there is? And then I said to myself, but still there is beauty [emphasis added]. And the fact that there's beauty says I think God is loving and wants to give us pleasure (14)	
	Due to what is achieved with Therapists	Definition:<br>"Every time I came away from a session I always felt better."<br>"I learned [through] going to therapy...that you have to keep going. You can't give up or...you'll just waste your life." (3)<br>"Then they [professionals] could not really help me. (6)<br>Doreen reported that Wilfred's therapist had admitted that ''he may never talk ... but then again, there is a chance it just could happen'' cause confusion, lacked clarity (8)<br>more speech therapy.<br>People tried to deal with uncertainty by obtaining advice or snippets of information from others, both professional and lay (8)<br>Discussions about therapy always included equal mention of the therapists themselves, their personality, style, and therapeutic relationship. "The happiest possible experience for me was to have Hazel as not only a professional helping Matthew but it was like a sister and a mother and a very good friend propping me up as well. She was all encompassing as far as I was concerned. She was wonderful" (8)<br>Despite this, many people were positive about therapy and found it worthwhile, devoting a great deal of effort to it. For some, the exercises themselves were perceived as the main reason for their improvement (8)<br>Discharge from therapy was particularly significant for those people who felt it left a gap or who felt disturbed by the uncertainty of knowing if they could have improved further. (8)	

		I've actually gone backwards because I move a lot less now, only when I have to. The therapy (at Beatrixoord) was like a big stick to keep me moving. (12)	
	Self-reflection – rethinking priorities	Definition:<br>"I'm still alive" : "Organising priorities in life"; "Before my stroke . . . deadline . . . quickly. And after my stroke . . . slow down . . . good." (2)<br>. These included expressing gratitude to still be alive and still able to "kiss my family, children and boyfriend." (4)<br>Over time, many of the participants became self-reflective, refocused on the meaning of family and marital relationships and began rethinking priorities in life (7)<br>I value my partner more and other relationships more. I have stopped worrying about the little things. The stroke put my life into perspective. "greater appreciation of each otherand the roles we play in the family. (7)<br>We stay closer to each other. Now we talk more about our problems, we are more open towards each other. We have stuck together all these 30 years [Eva, stroke survivor]. (9)	
	Awareness of condition – taking control	Definition:<br>becoming a member of an aphasia group enabled her to develop new friendships: "After just one session with her we understand that we are going to become best friends." (4)<br>the lesion itself. You can't change that but extrinsically, the care they receive, support from caregivers, how motivated that individual is, maybe their behaviour, their emotional state. (10)<br>Being able to express and discuss fear and anger (to professionals but also to others in a similar situation) was identified as part of the shift towards taking control and reaching positive outcomes. (11)<br>. He used the attitude "challenges bring out the best in you "to work through the physical and emotional issues that presented themselves following his stroke, as he and his wife viewed the stroke and its effects as a challenge. (14)	

		. His wife said later, ''That is typically Martin. He knows how to do this. He has always done this!'' One possible interpretation is that Martin was an active agent and a good medical manager, as he arranged the care and resources in order to deal with the consequences of the stroke.[8,32] We have interpreted this as an action of a role manager (16)	
**Level of independence**	Gained independence by doing thing by themselves	Definition:<br>Doing things – Independence: "By myself." The sense of independence gained from being able to do things "by myself", independence in ordering in a restaurant: "If you're going out for dinner . . . make sure that you are . . . you do it. With yourself," (2)<br>Lack of independence. Vulnerability due to the need to accommodate current functional limitations into daily life activities and feeling insecure about the ability to do so. (7)<br>Exercise<br>Yes, I really feel it [exercise] helps me and its an accomplishment that you're doing something you know.... because I don't want to be regarded as a disabled person, I think it's just basically you want to be what you were before and its an ambition (10)<br>Many participants felt that they had to work out by themselves what aids they needed, and how to get them. (12)<br>, ''We had made a list with different household activities that I could do. I was really happy at the end of the day. I had done all the household activities by myself, in my own way and own pace.'' When Jan had returned home, she had shown him what she had achieved at home (16)	
	Ability to mobilise somewhere (travel of functionally)	Definition:<br>. Staying busy and active, getting out of the house, and simply having the option to come and go as they pleased were other important aspects of quality of life. For these participants, the freedom to move around their community independently gave them<br>control over their day-to-day lives.	

		, "That I can if I want to. That I can go to the store if I want to, that I can go to church if I want to, that I can do the things I want to." (1)<br>Well, in our case, A could really just walk and we went out every day and it was finding places to walk.... anything to keep walking and keep mobile and do as much as he could...in the end we travelled all over [Female Carer, 80 years old (10)<br>. John did get a mobility scooter in the end, which enabled him to engage in activities such as grocery shopping (12)<br>Both Tom and his wife found the small mobility scooter and the parking permit ideal for their community mobility (12)<br>He received advice about traveling and since then "has put over 175,000 miles on [his] cane (14)<br>Gretal, a stroke survivor, described how she could go out of their high rise flat only if her husband (Vas) was with her due to her risk of falling. Rather than talking about being dependent on her husband she presented the two of them as 'a team'(15)<br>He said, ''Well you know, if I can walk again, I might also be able to drive a car again, go to the shop myself, or visit people outside my own village". Driving a car would not just be ''driving a car'', it would open up new possibilities and allow him to regain old roles' (16)	
	Overcoming barriers	Definition:	
	motivated/ hardworking/ positive "I Can" attitude facilitating recovery	Definition:<br>"I can." Another feeling engendered by participants engaging in "doing things" was a sense of ability and achievement. A strong focus on "I can" statements in participant transcripts highlighted the importance of recapturing feelings of ability for living successfully with aphasia: "I can do that"; "There's heaps of things round the place I can do"; "That's being able to do things . . . so I can—yeah." (2)	

		: “You gotta have an interest like carving or reading . . . it gives you something to work for. It gives something to try and succeed with something . . . You got to work towards something.” (2)<br>, “I think that—important thing with any sort of disability is your attitude towards it”; “Have a positive attitude”; “It’s simple—it’s be positive.” (2)<br>Sharon, who was only 44 when she had her stroke, viewed recovery as dependent on solid work. She told me ‘‘if you don’t work on it, it’s not going to happen for you’’. (8)<br>think ‘‘Well, I could have been away, how am I going to improve my life, really?’’ It frightened the life out of me....‘‘Oh, hey, come on. Shake up and do something, improve what you did before, improve your fitness, improve your lifestyle’’ (10)<br>Because I want to get better and I think that’s where the determination comes from, the fact you want to get better so you do things you’re supposed to do in hoping they will work. (10)<br>e evidence from the narratives suggested that the adjustment processes involved an emotional shift from negative to more positive emotions, (11)<br>expressions of fear throughout but more positive emotional reactions increasingly voiced: Fear (1 to 4)→Tearfulness (1)→Verbalisation & rationalisation (2 and 3)→Enjoyment, Hope & Increased independence (3 and 4). (11)<br>:“Em, I’ve got to be positive ...and just move on, do you know what I mean? It’s like er, I’ve always been a positive person but I’ve got to be more positive”. (11)<br>‘‘And if we go to the fishers village, we might also go to [our son] Jeroen’’ His wife laughed and said, ‘‘Well, if you have something on your mind, it will happen’’. (16)<br>. Determination at achieving post-stroke recovery and being an active ‘doer’ was reported, referred to by Charles’ wife as his ‘‘sheer guts and determination’’. (17)	

		coping Despite their feeling of fatigue, many patients felt a determination not to give in to such feelings. They wanted to feel independent, to push themselves when feeling fatigued, and to exert control and get things done. (18)	
	Family/ support systems – effect on independence	Definition:<br>This often meant that another person would have to accompany them for a significant part of the day. They could therefore feel that they were losing their independence.<br>As I'm not 'allowed' out alone (could you imagine that?—I wonder what they think I'm going to do?). (4)<br>I would not have managed without my wife. She has pushed me all the time, Without her, I would have stayed in the wheelchair and lived in a nursing home [Harald, stroke survivor]. (9)<br>The relationship was strengthened as they grieved the losses and searched for information together. Co-management (9)<br>As I said to you they either wrap you in cotton wool which is no a good thing, but that way you, you become an invalid then don't you, and the last thing I wanted to be was an invalid. [Male stroke survivor, 72 years old] (10)<br>. I tend to avoid it, escalators and that sort of thing … In my case, my husband also needs to feel like going out with me. Because Sarah was afraid of falling in public, she became dependent on her husband's support (12)<br>There were many things [she couldn't do], shopping, couldn't do gardening, cooking, she was just able to sleep. Even to eat was difficult. We had to help her eat … We had to help her to do everything, she can't lift her arm … she had to ask for help even to eat. (13)<br>, but the burden it put on others to assist with this essential task (13)<br>However, in some narratives the notion of a spouse as carer was an image that dominated the couples' co-presentation (15)<br>G: He have to do everything, he have to put my clothes on for me. So it is really bad. Really, he, he wash me. So I am really bad. I'm not hiding it and I can't, God give me him for a reason. (15)	

		However, she also said that he was too careful with her and in some ways even limited her. Two situations will be further described with possible explanations. "I need him, but he always cooks the same!" (16)<br>. Jan explained his internal conflict: he wanted to help Betsy, but he also wanted to prevent any mistakes she might make, as he experienced sadness when Betsy struggled in activities. (16)	
	Unsupported achievement of activities of daily living	Definition:<br>managing household jobs: "I can shop by myself (comment referring to photo of a shopping centre)"; and in travelling locally: "I get a bus to my stop and then I have ownership of it . . . But I do it myself." (2)<br>I told my wife: im going to move to another county and try to manage on my own give me two years[I I supposed if I had chosen to live with her, I would have made her do things for me now living on my own I cannot depend on her [Gunnar, stroke survivor]. (9)<br>The carer has to take a back step, quite often. Stand back, yeah, keep an eye on them without any accidents. It gives them a bit of confidence they've done. [Male Carer, 73 years] (10)<br>I'm not bothered about anybody helping me. I want to do it on my own. (James). (17)	

Stage b

Inital of Mind map –

Mind mapping –

**Meaningful Activities of daily living**	**Social Identity**	**Awareness of progress**	**Level of independence**
Achieving activities which are purposeful	Identified by what they can achieve	Acceptance	Gained independence by doing thing by themselves
Physical recovery defined successful living	Achieving a sense of purpose	Recognising doing nothing will cause regression	Ability to mobilise somewhere (travel of functionally)
Adapting the world around them	Role within the family (negative and positive)	Progression is gradual process (positive recipient/ frustration)	Overcoming barriers
Completing/ adapting old activities	Communication	Where they were compared to where they are now	motivated/ hardworking/ positive "I Can" attitude facilitating recovery
Risk taking	Meta-perception. Ways in which other see them (disabled/ disabling self by thinking this)	Denial and uncertainty	Family/ support systems reducing independence
Goal setting/ Achieving milestones (good and if unrealistic =bad)	Work identity	Realising limitations and capabilities, finding a balance of can and cannot	Unsupported achievement of activities of daily living
Pride in doing/ purpose	Recognising no longer old self but instead a new identity	Loss of hope due to inability	
Recognising success then tackling new challenges	Barrier due to comparing old self to new self	Change in behaviour – to see the world differently	
Developing routine and forward planning	Meaningful social relationships	Due to what is achieved with Therapists	
	Isolation/ social inclusion	Self-reflection – rethinking priorities	
		Awareness of condition – taking control	

Mind Map with reorganisation of themes

**Barriers and facilitators**	**Risk taking**	**Achievement of meaningful activities or interactions**	**Social Identity (past, present, future)**
Social<br>• Family/ support systems<br>• Meaningful social relationships<br>• What is achieved with Therapists<br>• Isolation and inclusion	Recognising doing nothing will cause regression	Achieving purposeful activities	Re-evaluation of social identity<br>• Recognising progress and goal setting<br>• Self-reflection and rethinking priorities

Physical<br>• Physical recovery defining hope<br>• Mobilisation status	Adapting the world and changing behaviour	Recognising success then tackling new challenges	Present social identity and adapted self<br>• Identified by what they can achieve<br>• Role within the family/ Work identity
Internal<br>• Acceptance<br>• Motivated and attitude<br>• Hope in possibility	Hope as a paradox, realise limitations and capabilities	Independence achieved by doing	**Future and possible social identity**<br>• Completing/ adapting old activities<br>• Achieving a sense of purpose and Pride<br>• Developing routine and forward planning<br>• Progression is a gradual process

Final Reduction and Reorganisation of themes

Theme	Subtheme	Code	Studies that supported
**Barriers and facilitators**	Social<br>• Family/ support systems<br>• Meaningful social relationships<br>• Due to what is achieved with Therapists<br>• Isolation and inclusion	**Definition**: This theme was defined as the collective impact of others on the individual<br><br>Family/ support systems:<br>This often meant that another person would have to accompany them for a significant part of the day. They could therefore feel that they were losing their independence. "As I'm not 'allowed' out alone" "they wrap you in cotton wool... last thing I wanted is to be an invalid" (4)(10)(16)<br>I would not have managed without my wife. She has pushed me all the time, without her, I would have stayed in the wheelchair and lived in a nursing home [Harald, stroke survivor]. (9)(15)<br>The relationship was strengthened as they grieved the losses and searched for information together. Co-management (9)(15) (16)<br>There were many things she couldn't do so she became dependent on her husband's support (12)(13)<br><br>Meaningful social relationships:<br>Defined as relationships which were built overtime upon a mutual understanding.<br>Factors in their lives were seen as contributing to living successfully with aphasia (e.g., "The most important thing is my partner"). (2)(6)<br>As one participant stated: "I think it's really people . . . that make you feel successful." (2)<br>Friends were referred to with great appreciation: "They are my rock and my true north" (4)<br>relationships built up over time were viewed as the key to overcoming isolation rather than simply "getting out (of the house)", (11)(16)<br>Visits to and from friends could reduce negative mood states – "felt less bored and down" (18)<br><br>Due to what is achieved with Therapists:	(ID:1, 2, 3, 4, 6, 8, 10, 11, 12, 13, 15, 16, 17, 18)<br>Total: 14/18

		Definition: recognising what is achieved due to the progress made with therapists. This was displayed<br>“Every time I came away from a session I always felt better.”<br>“I learned [through] going to therapy…that you have to keep going. You can’t give up or…you’ll just waste your life. “Therapy was like a big stick to keep me moving”. (12)(3)(8)<br>“Then they [professionals] could not really help me. (6)(8)(17)<br>“The happiest possible experience for me was to have Hazel as not only a professional helping Matthew but it was like a sister and a mother and a very good friend propping me up as well. She was all encompassing as far as I was concerned. She was wonderful” (8)<br><br>Isolation and inclusion Mete perception:<br><br>Definition: This was often due to the individual’s meta-perception that they were less-able rather than the true reality and their ability to communicate.<br><br>In particular, the strong impact of communication on the loss of friendships was evident “Even if it’s friends coming here. You don’t know how to (indicates to mouth) . . . they’re coming and say hello [but not the same conversation].” “He cannot start a conversation; sometimes, when we have visitors, he leaves the room “(2) (9)(4)<br>They could feel unsociable, anxious, inclined to avoid crowds and preferred to spend time alone. (4) (6)<br>You do lose quite a few friends because you can’t do everything independently any more (12)<br>“I went to see a second grandson play tennis…and as I approached every eye is on me, and here I am walking with a limp, and have to be helped to my seat, I just have the feeling that all of them said, “look at that man who is cripple, who is lame and cannot do for himself,” and then I start crying. (1)<br>Jess described her difficulty participating in a game due to fatigue, leading others to assume she was not intelligent which she found upsetting: “And the FRUSTRATING thing is that is that people think it’s because you have a low IQ, which isn’t the case (4) (11)<br>Communication:	

		Humour - Participants commented: "And I laugh. I just laugh"; "You can sort of take funny—funny sides of it . . . make a joke out of it"; "But laugh. You've got to laugh." (2)<br>Humour – One of the frustrating things about aphasia for me is the lost opportunities to make snide remarks. I'm too slow … I couldn't say it (4)<br>Humour - previous role as the quick-wit and raconteur, even though his physical and language impairments were, on the surface, relatively mild. (2)<br>for example reading "newspapers" and "books"; being able to give a "talk" or "speech" at a church or community group; writing a signature "so that I can go shopping" or "at work . . . sending emails". In many cases participants reported restrictions in being able to carry out activities because of communication difficulties (2)	
	Physical<br>• Physical recovery defining hope<br>• Mobilisation status	Physical recovery defined successful living:<br>As activities were resumed or replaced, survivors also regained a sense of their self-confidence, competence, personal influence, wholeness and meaning. (1)(8)(11)<br>and provided alternative ways of doing things. (18)<br>"I am so tired, the fatigue is getting better, I am no spring chick any longer … really tired me out visiting family." Feelings of unhappiness resulted from the lack of ability to participate in family gatherings (4)(6)(18)<br>We find that Mette constructs her ABI primarily at a physical level. (6)<br>Men in this study measured their recovery by the extent of physical recovery attained (7)<br>Loss of hope due to inability<br>Even simple things are difficult to do… it's a constant effort" (18)<br>"You lose hope because you can't get better. You know you can't. So it goes by the board" "as the months passed and no functionally significant improvement was made, this energy, time commitment reduced" (3)(13)<br>"If you don't have hope you have nothing, it just gets hold of you and that's it. You wouldn't have anything to fight for (3)<br>This frequently resulted in a tension between accepting impairment and maintaining hope (11)<br>The paving stones are hopeless, you soon discover that if you can't walk properly, all paved paths are crooked. That means that I go out less often than I could. (12)	ID: 1,2, 3, 4, 6, 7, 8, 10, 11, 12, 13, 14, 16, 18<br><br>Total: 14/18

		Mobilisation status<br>Defined as the ability to travel with confidently to a destination<br>They also described limited options in terms of places they could visit, for example, not being able to go further than the local shop. (4)(1)<br>“Because I can’t get out there and do what I want to do. So—just got to stay here to do nothing.” (2)(10)(18)<br>(mobility) would open up new possibilities and allow him to regain old roles’ (16)<br>Freedom to move around the community independently gave control over their day-to-day lives (1)<br>“Well, in our case, A could really just walk and we went out every day and it was finding places to walk, keep mobile and do as much as we could” (10)<br>He received advice about traveling and since then “has put over 175,000 miles on [his] cane (14)<br>Some patients did get a mobility scooter in the end, which enabled him to engage in activities such as grocery shopping (12) (14)	
	Internal<br>• Acceptance<br>• Motivated/ hardworking/ positive “I Can” attitude<br>• Comparing old self to new self /Denial and hope in possibility	Acceptance:<br>This was defined as coming to terms with what had happened and having the option to change behaviour.<br>“I’ve got to accept it.” “moving ahead and enjoying life” For many participants acceptance was also a component of striving for a positive way of life. (for example, driving: “I can’t drive . . . To me that’s a big negative. [But] It’s not negative thinking. It’s . . . accepting I suppose”); (2)(16)<br>“I guess I just accepted it, because you cannot do anything about it, I guess I had to accept it.” (3)(5) (7) The progression included a variety of positive outcomes (themes of increased independence, enjoyment, hope, looking to the future and aspirations, openness and understanding (of others), finding a sense of worth, and crusade) coinciding with increasing acceptance. (11)<br>, ‘There a lot a people ‘alf our age who’re worse off than us, ain’t they, you know what I mean’ (15)<br>Most participants described acceptance of the issues identified through a process of adjustment (17)	ID: 1, 2, 3, 5, 6, 7, 8, 10, 11, 15, 16, 17, 18<br><br>Total: 13/18

		Some patients found it hard to accept their condition and the change in their perception of self. They found it “hard to accept myself … I need to give myself a slap” (18)<br><br>Motivated/ hardworking/ positive “I Can” attitude<br>Defined as an internal motivation to defy the odds to exert control over living successfully.<br>“I can do that” Another feeling engendered by participants engaging in “doing things” was a sense of ability and achievement. A strong focus on “I can” statements in participant transcripts highlighted for determination to exert control and to live successfully (2) (17) (18)<br>“I think that—important thing with any sort of disability is your attitude towards it”; “Have a positive” attitude. (2) “if you don’t work on it, it’s not going to happen for you’’ (8) (10) (11)<br>Adjustment processes involved an emotional shift from negative to more positive emotions, (11)<br>‘‘And if we go to the fishers village, we might also go to [our son] Jeroen’’ His wife laughed and said, ‘‘Well, if you have something on your mind, it will happen’’ (16)<br>(17)<br><br>Comparing old self to new self /Denial and hope in possibility:<br>Choosing to compare the old identity to the new identity or embracing the new identity defined denial or hope. This conflict of previous identity was present throughout many of the __ studies (ID) associated with this code stating “There’s more to me than what you see. I was such a vibrant person… Now there’s nothing.”(1,2) and “I was the clown, who went to disco’s, now I can’t have one drink” (6). These feeling were followed by anger and vulnerability for the future (11).	

		"There's more to me than what you see now. I used to get out, could outrun anyone, most of the average person, I played ball and I was a typical teenager, and I was such a vibrant person… There's nothing." (1)(2)<br>"Both of us have found the effect of the stroke absolutely devastating because we were, you know, we were starting to do things. Like we'd started going on walking and dancing holidays and we were starting to do things like that that we found really, really enjoyable, and I suddenly can't do them anymore." (5) (7)<br>You see, I am the social Mette, who has always been "the clown," who got totally wasted at the disco, right. […] And now, I cannot even consider having one drink (6)<br>Michael, a 69-year-old farmer, described his prognosis as ''unbeknown'' (8)<br>She was 61 years old and felt that she was too young to be seen somewhere for therapy which was associated with more elderly and disabled people. (8)<br>Most references to anger included the term "frustration" and the theme was closely linked with uncertainty (about the future) and vulnerability (11)	
**Risk Taking**	Recognising doing nothing will cause regression	Definition: This sub-theme was defined as understanding that changing nothing may cause no change "The worse thing they could do . . . You sit at home in a house . . . watch TV—no no. That's not right (laughs)"; "If there's most people around that say, oh I'll do nothing. They're going downhill" (2)<br>"[Hope has to be the backbone.] It has to be, because if you have a negative attitude you are not going to get better. You are just going to dwell on your problems and they will get worse." (3) (14)<br>"Trying new things and do it," one participant suggested. Another said, "I have to take risks so I can get further on" (2) (16)<br>The adjustment processes therefore concerned increasing independence (linked with the personal processes of increasing confidence and self-determination), trying "new things", and ultimately modifying environments. (11)	ID: 2, 3, 11, 14, 16<br><br>Total: 5/18
	Adapting to the world around them	Defined as the ability to develop specific coping strategies by adjusting the environment around them.	ID: 2, 4, 11, 12, 16, 17

		Adapted by trying something completely new and different: “I wouldn’t have thought about floristry until after my stroke . . . I was . . .working in the office before my stroke (referring to photo showing a flower arrangement).” (2) (11)<br>Aphasia - adapted their environments to assist understanding—for example, captions on the TV, or “big books [large print books] (2) (17)<br>Struggling to follow group conversations resulted in a preference for meeting friends one-to-one for some participants (4)<br>Because of the uncertainties John experienced, he decided to move to a single-storey apartment (12)<br>He used others in order to control and manage the situation - which can be viewed as proxyagency (16)	Total:<br>6/18
	Hope as a paradox to realise limitations and capabilities	This theme was defined by simultaneously accepting and wanting to defy to understand potential. “I had a belief that I am cured. . . . I’m better now. I could say one hundred percent better now.” His considerable recovery, his limited day-to-day activities, and the fact that he had lived with the impacts of stroke for 7 years could explain why he felt recovered despite continuing limitations (5) “And I am finding that I am living successfully with the stroke (2)<br>“So I try to see that I still contribute. I do not go to so many meetings. I only go to the meetings where I could contribute something” (5)<br>“Yes, I am fine lying in my bed the plans I have and what I am going to do and what I’m not going to do” (10)<br>Mike was able to adjust his pace and the way he tackled the stairs, depending on the situation, which shows considerable flexibility. (12)<br>She had to think consciously about every push on the pedals to prevent herself from falling. Despite this, she developed strategies that enabled her to enjoy cycling again, pacing strategies (12)<br>“It is a process of endless tinkering, weighing, adjusting, and coordinating mobility practices in each situation anew” this needs to be acknowledged by the rehabilitation team (12)<br>She allowed him to engage in “controlled adventuring” by protecting him from situations she knew would be too frustrating for him or from taking on too much (14)	ID: 2, 5, 10, 12, 14, 16, 17<br><br>Total:<br>7/18

		coming to terms with their health and life changes, or a focus on things they can do (rather than those they cannot). (17)<br>She also said Martin had not changed after the stroke: ‘‘He is still orchestrating the people around him. He has people for all kinds of activities” (16)	
**Achievement of meaningful activities or interactions**	Achieving activities which are purposeful	This sub-theme was defined as the ability to undertaking tasks which had a specific purpose to the individual. As survivors reengaged in meaningful activities, their experience of quality of life changed over time (1)<br>“Be involved with everything.” “Have a hobby.” “Keep you—spirits up—yeah.” “Live as much as you can; do as much as you can” “doing things” as a core component of living successfully (2)<br>“Doing little was described as counteractive to living” – activity has to have a purpose (2)<br>achieved a sense of worth (11)<br>“You gotta have an interest like carving or reading . . . it gives you something to work for. It gives something to try and succeed with something . . . You got to work towards something.” (2) (8) (17)<br>For example, Teilhard particularly enjoys the process of developing his own film, and he figured out a modification that would substitute for his left hand to allow him to move the film to a developing reel in his darkroom. “I was really able to get back to a lot of things that were important to me”. (14)<br>‘‘Jan proposed that if he first prepares everything and puts it on the gas, I could sit in the kitchen beside the gas cooker and control the potatoes when they were boiling. But well, I can better read a newspaper. I stopped cooking’’ (16)	ID: 1, 2, 8, 11, 14, 16, 17<br><br>Total: 7/18
	Recognising success then tackling new challenges	Definition: The sub-theme is defined as using success as a springboard for greater risk-taking<br>It’s getting confident to do things. I find the first time you do it, you are a wee bit apprehensive, but then you’ve done it. You’ve achieved it and feel brilliant, so don’t think twice about doing it again. (10)<br>Participants identified benefits in “getting around” impairment, such as setting compensatory goals in the “What Next?” group’s, and practising new strategies in “safe” environments (11)<br>Martin often reflected on everyday activities testing future possibilities with his wife and researcher (16)<br>“I always try to do what I can within reason …I enjoy the challenge”. (18)	ID: 10, 11, 16, 18<br><br>Total: 4/18

	Gained independence by doing thing/ unsupported achievement of activities of daily living	Achievement of unsupported activities of daily living defined with meaning defined this sub-theme Doing things “By myself.” The sense of independence gained e.g. ordering in a restaurant: “If you’re going out for dinner . . . make sure that you are . . . you do it yourself,” (2) carers taking a step back (10)<br>Lack of independence. Vulnerability due to accommodating limitations in daily activities and feeling insecure about this (7)<br>Exercise: “Yes, I really feel it, helps me and its an accomplishment that you’re doing something for yourself.... because I don’t want to be regarded as a disabled person, you want to be what you were before (10)<br>‘‘We made a list with different household activities I could do. I was really happy at the end of the day. I had done all the household activities by myself, in my own way and own pace.’’ When Jan had returned home, she had shown him what she had achieved at home (16)<br>Managing home jobs: “I get a bus to my stop and then I have ownership of it . . . But I do it myself.” (2)<br>“I told my wife: I’m going to move to another county and try to manage on my own give me two years [I supposed if I had chosen to live with her, I would have made her do things for me” (9)<br>“I’m not bothered about anybody helping me. I want to do it on my own” (17)	ID: 2, 7, 9, 10, 16, 17<br><br>Total 6/18
**Social identity (past, present, future)**	Re-evaluation of social identity<br>• Recognising progress and goal setting<br>• Self-reflection of identity and rethinking priorities	Definition: goal setting<br><br>A real sense of achievement was evident for participants in describing these milestones: “I got the job . . . I accomplished something that I didn’t think I’d be able to do” (2)<br>Setting goals ‘‘I’d like to get independently to the hairdresser’’ “when he came out, we set goals” (10) to let go of particular pre-stroke pleasures, and see what was needed to reformulate goals daily (12) (14)<br>Where they were compared to where they are now:<br>Just gradually changing and gradually getting better.” (2)<br>“When I had my stroke—two years [ago]—I can see how far I’ve come . . . . I had no voice and couldn’t speak. Now I can talk properly”; “I feel quite . . . successful (2)	ID: 2, 4, 5, 6, 7, 9, 10, 11, 12, 13, 14, 17<br><br>Total: 12/18

		By reflecting on the beginning stages post stroke and progress they had made, participants were hopeful for continued recovery in the future. (3)<br>“I would write down each week things she was doing and compare that with previous weeks, it’s amazing actually what difference there is” (10) (17)<br>There’s things I can’t do, the list of things that I could not do was a great and the list of things I could was very small. So over the last year, I’ve transferred things over from “can’t do” to “can do,” and the lists are beginning to settle up. (5)<br><br>Recognising no longer old self but now a new self:<br>“I’m trying to . . . get as close as I [can to what I was]—before the stroke . . . I know that that’s not possible, but . . . as good as I can get and I’m happy.” (2) “we just have to compensate” (6)<br>“It’s as if I had one life and it ended when I had the stroke, and I now have a completely new life” (5)<br>“I am entering a transitional period, where I’ll have to learn to get over my past perceptions of self-image. Perhaps it is time to focus on other things. (7) “you feel a different person altogether” (11)<br>Moreover, the task of “Looking back, around and forward” was facilitated by an integrated consideration of all aspects of life, and to envisage these aspects together when looking forward was part of reforging links between the different aspects of self and the reworking of self-image. (11)<br>Self-reflection and reprioritisation:<br>I’m still alive: “Organising priorities in life”; “Before my stroke . . . deadline . . . quickly. And after my stroke . . . slow down . . . good.” (2) (13)<br>Gratitude to still be alive and still able to “kiss my family, children and boyfriend.” (4) (7)<br>“Now we talk more about our problems, we are more open towards each other” (9)	
	Present social identity and adapted self	Identified by what they can achieve:<br>Karen felt influential in her new job: “I think I’ve made a positive contribution to the company as a whole” (5)(13) A sense of purpose (where activity had personal, religious or community meaning) (11)	ID: 2, 4, 5,6, 7, 9, 11, 13, 16

	• Identified by what they can achieve<br>• Role within the family /Work identity	A stroke survivor had been well known in the community for his ability to call people to prayer. His stroke left him too weak to continue this role, which had clear social consequences, (13)<br>Cooking and household chores were her pre-stroke roles, and doing them again was a way to regain these roles as well as a sense of self and identity (16)<br><br>Role within the family/ work identity<br>A photo of her putting on make-up: “I’m learning how. . . very important. Because my husband’s not a woman. So, I want to do myself. Very important” (2)<br>Emily prior to stroke was responsible for her 3-year-old’s upbringing. Post stroke, her husband became the main carer. “Up until now, he was truly a mama’s boy, Now, husband is the playmate, the reader, the explain it all person.” (4) (5) (6)<br>Andrew felt he had lost his role as a man and a husband within the family and was worried his wife might leave: “Well, it certainly changes things in terms of your role within the family because certainly “now I’m dependent now rather than a partner. What sort of a husband am I” (5)<br>“the stroke has made me useless. I can’t do things for my family like protect them” (7)(13)<br>“The stroke was destructive, my daughter moved out at age 16. I think she wanted to be far away” (9)<br>For some, loss of work entailed loss of the social contact which went with the work environment. (4)(7)<br>When the researcher asked Martin 21 months later about the biggest changes, he responded ‘‘Not being able to work. That is the biggest change” His wife said that work was ‘‘his everything’’ (16)(11)	Total:<br>9/18
	**Future and Possible social identity**<br>• Completing/ adapting old activities	Definition: completing and adapting old activities<br>“You didn’t think about it before you had the stroke, but, now, after the stroke, it means a lot to be able to do the things you could do before.”(1)(5)<br>Participants continued activities from before their stroke, adapting old activities to enable participation, and finding new interests and activities. (2)(3) doing what you can, accepting things you cannot do (17)	ID: 1, 2, 3, 5, 7, 8, 9, 10, 11, 12, 13, 14, 16 17, 18<br><br>Total:<br>15/18

	• Achieving a sense of pride and purpose<br>• Developing routine and forward planning<br>• Progression is gradual process (positive recipient/ frustration)	"i will not be getting any better" noting that instead of looking for changes "I am learning to adapt" (7)<br>By playing solitaire he used this to train himself to pay attention to his left side as he recognized early on that he had to address this issue in order to get back to other occupations, such as driving (14)<br><br>Definition: Achieving a sense of purpose<br>"Mimi was not just saying that caring for her family makes her happy, although she stated that it is "the greatest joy" she has. She was making the point that caregiving makes her feel healthy, capable, and accomplished. Her quality of life is enhanced" (1)(3)<br>"My son's a little spoiled, but my only job is to be a good mother, or I'll feel I have abandoned him" (9)<br>"I need to be better for my family 'cause they need me, I want to see my grandchildren growing up" (10)<br>She repeatedly talked about her visits to an African family to coach them in Dutch language and culture and her time at the local activity centre where she helped to serve lunch once each week (16)(13)<br>Pride in doing:<br>One participant described a self-portrait taken for the photo interview: "There's me. And I am a success with aphasia." (2) Dave next door is a professor, and he says from time to time ''He is a miracle'' (17)<br>She was proud about her achievements in the Art exhibition, hopeful at being "a little bit better. (11)<br>"This picture impresses me because I can help my children by finding firewood. It means I can already make an effort" (13)<br>Teilhard used several words to describe himself including stubborn, tenacious, over-achieving. He describes himself this way both before and after his stroke, indicating his continuity of identity (14)<br><br>Definition: developing routine and forward planning:	

		After some time, the changes in marital life became routine. Couples did not reflect much on the past; rather, they wanted to look forward (9)(18)<br>Ben explained that although he did go out, he sometimes struggled if something unexpected happened, like a second floor of a shop without a lift. They planned how, when and with whom they would go (12)<br>“When I go shopping I always prepare a shopping list the day before. I know exactly where everything is in the shop. So I write my list based on the route I take through the shop” (12)<br><br>Progress is gradual<br>Nina was aware of her limitations but was hopeful that her limitations would not last. As she was able to resume doll making, In her mind, she was not stuck at one place in time but rather was working along an ever-changing continuum toward a better quality of life.(1) “everything is changing all the time”. (11)<br>I think you’ve just got to . . . take every day after the next one. . . Sometimes . . . you take three forward and then you drop back two. But then you sort of pick it up again. So, it is an ongoing problem.” (2) (3)<br>Recognition and adaptation would be a continuous process, perseverance and resiliency would be necessary in ensuring the family would remain intact. (7)<br>James had invested lots of effort in therapy founding it helpful to a degree. Over 2 years since his stroke, frustration and anger existed alongside a resignation that he no longer had control over his recovery: (8)<br>You’ve got to sort of face up to the fact that you’re not going to just manage things as easily ....<br>My life’s changed obviously, I mean, life has changed totally but I think I’m adjusting. (17)	

Note.: 1=Bourland, Neville and Pickens.,(2011),2=Brown et al.,(2010),3=Cross & Schneider.,(2010),4=Fotiadou et al.,(2014),5=France et al.,(2013),6=Glintborg & Krogh.,(2015),7=Green & King.,(2009),8=Hersh.,(2009),9=Kitzmüller, Asplund and Häggström., (2012),10=Morris et al.,(2014),11=Mumby & Whitworth.,(2013),12=Nanninga et al.,(2017)13=Norris, Allotey and Barrett.,(2011),14=Price et al.,(2010),15=Radcliffe, Lowton and Morgan.,(2013),16=Satink et al.,(2016),17=Shannon, Forster and Hawkins.,(2016),18=Young et al..,(2013)

Presentation of findings from table

3.4. Major Theme 1: Barriers and Facilitators

This first major theme identified three sub-themes: (a)Social, (b)Physical and (c)Internal.

3.4.1. Sub-theme (a): Social

This sub-theme was defined as the collective impact of others on the individual. This sub-theme had support from 14/18 studies (ID:1,2,3,4,6,8,10,11,12,13,15,16,17,18) and is divided into four codes.

3.4.1.1. Code 1: Family support systems

This code was defined as the description and context for how relationships can be valuable but also challenging. This code was supported by 7 studies (ID: 4,9,10,12,13,15,16). The value of family support was that people could access more activities of daily living and had increased motivation to engage with meaningful activities and interactions. An individual in study 4 said "*without her (his wife), I would have stayed in the wheelchair"*. The challenge of family support was that people felt they had lost their choice and independence due to the interference of others for instance a participant in study 10 stated: "*they wrap you in cotton wool, last thing I wanted is to be an invalid"*. These findings suggest that although positive reinforcement is important, family support may become too over-protective, preventing progress for the individual by not allowing them to attempt activities.

3.4.1.2. Code 2: Meaningful social relationships

Defined as relationships which were built overtime upon a mutual understanding, this code was supported by 6 studies (ID: 2,4,6,11,16,18). This included not just "*getting out (of the house*)" as stated by an individual in study 11, but instead visiting friends or old work colleagues to recapture previous identity (6). This was linked to improving individuals mental and social wellbeing (18).

3.4.1.3. Code 3: What is achieved with Therapists

This code was defined as recognising what is achieved due to the physical recovery within rehabilitation with therapists. This was supported by 5 studies (ID:3,6,8,12,17) and encompassed many positive experiences. For instance, an individual in 12 stated that "*Therapy was like a big stick to keep me moving*". Some studies had contrasting opinions of what was possible from therapy including studies 6,8 and 17 stated that individuals believed therapists couldn't help them. Furthermore, once therapy was taken away post-discharge it could leave individuals unsure of what change was possible. For instance, one study stated that individuals "*felt it left a gap or uncertainty of further improvements*" (8).

3.4.1.4. Code 4: Isolation and inclusion

Isolation was defined as differing perceptions of reality regarding an individual's capability which included their meta-perception of being less-able rather than the true reality. This theme was present throughout 7 studies (ID: 1,2,4,6,9,11,12). A key finding was that loss of communication with others may isolate an individual (2,9,4). For instance, study 2 stated *"Even if it's friends coming here. You don't know how to (indicates to mouth)"*. This was a definitive barrier to inclusion with social situations, increasing frustration during social interactions. However, humour was identified as one way to overcome such experiences (2).

3.4.2. Sub-theme (b): Physical

This sub-theme describes that an individual's perceived ability to function physically was partly determined by how successfully they thought they were living post-stroke. Fourteen studies identified content for this sub-theme (ID:1,2,3,4,6,7,8,10,11,12,13,14,16,18) and divided into two codes.

3.4.2.1. Code 1: Physical recovery

Physical recovery is thought to be a key measure for recovery and defined living successfully post-stroke within the literature with 10 studies expanding on this (ID:1,3,4,6,7,8,11,12,13,18). Physical recovery impacts individuals differently with some regaining a sense of self-confidence and feeling human (1,8,11). Others feel more physically able to undertake activities within the family (4,6,18) and some are influenced by their perception of change to masculinity or feeling feminine (6,7). If the individual believes they cannot achieve these due to their inability. It was stated by one individual that "*You lose hope because you can't get better"* (18).

3.4.2.1. Code 2: Mobility status

This code can be defined as the ability to travel with confidence to a destination. Its supported by 8 studies (ID:1,2,4,10,12,14,16,18) elaborating that the distance travelled was not the defining factor of an individual's mobility status but instead what the journey meant and the context of the destination. For instance, one individual stated *"if we travel to the fishing village, we could see our son*" (16). For others, it allowed independence and freedom. This has the opposite effect when mobility wasn't achieved (2,10,18). One individual stated *"Because I can't get out there and do what I want. So—just got to stay here to do nothing*" (2).

3.4.3. Sub-theme (c): Internal

This sub-theme was defined by the emotional health of the individual and reflects the individual's character. This sub-theme had support from 13/18 studies (ID:1,2,3,5,6,7,8,10,11,15,16,17,18). Below are the three codes divided from this sub-theme.

3.4.3.1. Code 1: Acceptance

This was defined as coming to terms with what had happened and having the option to change behaviour. This code was shared with 9 studies (ID:2,3,5,7,11,15,16,17,18) with many positive perspectives stating "*I guess I just accepted it, because you cannot do anything about it*" (3,5,7). The option to change

behaviour allows individuals the choice to "*move ahead and enjoying life*" (2,6) or to defy this and still pursue their previous identity. Some individuals recognised their progress compared to other and gained acceptance from this (15).

3.4.3.2. Code 2: Motivation and attitude

Defined as an internal motivation to defy the odds to exert control over living successfully. This was supported by 7 studies (ID:2,8,10,11,16,17,18) finding that "Having a positive attitude"(2) and the willingness to engage in "doing things" (2,17,18) will allow for an emotional shift from negativity to positivity, facilitating hope.

3.4.3.3. Code 3: Hope in possibility

The ability to consider the past, present and future social identity could be met with contrasting reactions from denial towards hope as the ability to accept the potential change varied. This conflict was present throughout many of the 7 studies (ID:1,2,5,6,7,8,11). For instance, one individual stated "*There's more to me than what you see. I was such a vibrant person, Now there's nothing*." (1,2) and "*I was the clown, who went to disco's, now I can't have one drink*" (6). These feeling were followed by anger and vulnerability for the future (11).

3.5. Main theme 2: Risk taking

The second major theme identified three sub-themes: (a)Recognising doing nothing will cause regression, (b)adapting to the world and changing behaviour and (c)Hope as a paradox, realising limitations and capabilities.

3.5.1. Sub-theme (a): Recognising doing nothing will cause regression

This sub-theme was defined as understanding that changing nothing may cause no change and is supported by 5/18 studies (2,3,11,14,16). An individual in study 2 recognised that "*The worst thing you could do is sit around, you're going downhill*!" which is important as a facilitator towards risk taking. An individual from study 16 stated "*I have to take risks so I can get further*". This trial and error approach was identified as a way to take a step toward positive psychological adjustment and independence (11).

3.5.2. Sub-theme (b): adapting to the world and changing behaviour

This sub-theme was defined as the ability to adjust to the environment around them, enabling access to meaningful social roles, activities and interactions. Six studies (ID:2,4,11,12,16,17) supported this and suggest that adapting and attempting something different and taking risks may assist with transition to successful living (12). Study 2 and 17 supported this. One stroke survivor stated from study 2 "*I wouldn't have thought about floristry until after my stroke, I was working in the office*".

3.5.3. Sub-theme(c): Hope as a paradox, realising limitations and capabilities

This sub-theme defined that overtime through experience and information individuals were able to acknowledge what has happened and understand their capabilities and limitations. This is the critical difference from the above sub-theme, as due to their adapted behaviours, individuals now understand how to cope in daily life. This sub-theme had support from 7/18 studies (ID:2,5,10,12,14,16,17). An individual from study 12 stated "*It's a process of endless tinkering, weighing, adjusting, and coordinating mobility practices in each situation anew*" where this individual had learnt to constantly adjust and take further risks to realise capabilities on the edge of limitations. Even with high levels of impairment, some individuals still take risks and manage to orchestrate support around them (16) whilst other participate in family lead "*controlled adventuring*" (14).

3.6. Main theme 3: Achievement of meaningful activities or interactions

The third major theme identified three sub-themes: (a) Achieving purposeful activities, (b) Recognising success and tackling new challenges and (c) Independent achievements by doing.

3.6.1. Sub-theme (a): Achieving purposeful activities

This was defined as the ability to undertake tasks which had meaning to the individual and was supported by 7/18 studies (ID:1,2,8,11,14,16,17). The main finding from this was ensuring the activity was purposeful to achieve a sense of worth as doing little was described as counteractive to living (2,11). If this criteria wasn't fulfilled and activities are undertaken as a chore, then motivation and QOL will not improve over time (1). For instance, one individual stated that "*he suggested I sit and watch the potatoes boil, but well, I can better read a book. I stopped cooking''* (16).

3.6.2. Sub-theme (b): recognising success and tackling new challenges

The sub-theme is defined as using success as a springboard for greater risk-taking and is supported by 4/18 studies (ID:10,11,16,18). These studies recognised overcoming challenges allowed for the question "*what's next*" (11) and gave the individuals the insight into a potential future; *"I always try to do what I can within reason, I enjoy the challenge*" (18).

3.6.3. Sub-theme (c): Independent achievements by doing

Achievement of unsupported meaningful activities of daily living defined this sub-theme with 6/18 studies (ID:2,7,9,10,16,17) reporting this. By doing things autonomously, individuals gain a sense of purpose and begin to develop into identity roles by what is achieve. Study 16 states "*We made a list with different household activities I could do. I was really happy at the end of the day. I'd done all the household activities by myself, in my own way and own pace*". This example described the evolution from stroke survivors to independently achieving to gain a sense of not being a burden. However, adapting to independence may be difficult with some stating they felt "*vulnerable*" and "*feeling insecure*" when accommodating limitation to daily life (7).

3.7. Main theme 4: Social identity (past, present, future)

The fourth major theme identified three sub-themes: (a) Re-evaluation of social identity, (b) Present social identity and adapted self and (c) Future and possible social identity.

3.7.1. Sub-theme (a): Re-evaluation of social identity

This theme was defined as the ability to reflect and plan on present circumstance. This sub-theme had support from 12/18 studies (ID:2,4,5,6,7,9,10,11,12,13,14,17). This sub-theme was divided into two codes.

3.7.1.1. Code 1: Recognising progress and Goal setting

This was defined as the ability to measure the goals individuals achieve by comparing themselves at initial onset to present day. This was supported by 7 studies (ID:2,3,5,10,12,14,17) and identified how individuals are able to gain hope from realising that progression and recovery is occurring "*When I had my stroke two years ago, I can see how far I've come, I couldn't speak. Now I can talk. I feel quite successful*" (2). Others recognise this recovery by setting and measuring goals (10,17). This is crucial to the progression of identity, as seen from an individual in study 2 "*I got the job, I accomplished something that I didn't think I'd be able to do*".

3.7.1.2. Code 2: Self-reflection of identity and rethinking priorities

This is defined as the ability to recognise what is important in life and accepting the new identity as a result. With support from 8 studies (ID:2,4,5,6,7,9,11,13) recognising that the individual could no longer be associated with their previous social identities. Rather, they were required to develop new social identities as their life changes. One individual stated "*It's as if I had one life and it ended when I had the stroke, and I now have a completely new life*" (5). Study 11 discusses a strategy of looking back, around and forward to facilitate a consideration of all aspects of life and reworking self-image (11). Thus, this reflection of self allows the individual to re-evaluate their priorities which may be different to their previous identity (4,7).

3.7.2. Sub-theme (b): Present social identity and adapted self

This sub-theme was defined as the present understanding of the individual's perception of self and the adjustment to life. This sub-theme had support from 9/18 studies (ID:2,4,5,6,7,9,11,13,16) and was divided into two codes.

3.7.2.1. Code 1: Identified by what they can achieve

This is defined by what can be presently achieve and how this may change and individual's perception of self-identity. This is supported by 4 studies (ID:5,11,13,16) with study 13 discussing how one individual was identified pre-stroke within the community for his religious role. Post-stroke he couldn't continue this which resulted in social consequences and loss of identity (13). This may also cause loss of motivation resulting in slower recovery. Studies 5 and 13 however found individuals reported that their pre-stroke roles included *"Cooking and household chores and doing them again was a way to regain a sense of self and identity".*

3.7.2.2. Code 2: Role within the family /Work identity

Achievement as a means of role development within a social setting defined this code and was supported by 9 studies (ID:2,4,5,6,7,9,11,13,16). Within the literature there was many negative experiences regarding the impact to individuals and the collateral damage to the family as a unit "*The stroke was destructive, my daughter moved out at age 16. I think she wanted to be far away*" (9) or individuals who were previously identified by their role at work not being able to return (16,11). However, when some recovery was established, one woman in study 2 discussing the importance of applying make-up again to re-establish the role of wife and attraction to her husband.

3.7.3. Sub-theme (c): Future and possible social identity

This theme was defined as the individuals desired future to live successfully post-stroke. This sub-theme had support from 15/18 studies (ID:1,2,3,5,7,8,9,10,11,12,13,14,16,17,18) and is divided into four codes.

3.7.3.1. Code 1: Completing/ adapting old activities

The different compared to main theme 3 is that this code is defined by how much pre-stoke activities can be undertake or how they plan to adapt to complete these post-acceptances. This is supported by 7 studies (ID:1,2,3,5,7,14,17) and recognised the importance for old activities "*You didn't think about it before you had the stroke, but now, it means a lot to be able to do things you could do before*."(1,5). At this point individuals understand that they may not get better and accepting the need to adapt (7) and discuss finding new hobbies. For instance, one participant stated this as "*doing what you can, accepting what you cannot*" (7). This acceptance is thought to reduced anger and frustration resulting in increased QOL (2).

3.7.3.2. Code 2: Achieving a sense of purpose and Pride

This was defined as gaining purpose and pride in responsibilities to develop role and identity. Ten studies (ID:1,2,3,9,10,11,13,14,16,17) supporting this with study 1 and 3 both identifying that caring for others allowed for a sense of accomplishment, making individuals feel capable. Another study found being able to care for children gave pride in the identity of being a parent (9). Furthermore, one participant explained "I *need to be better for my family 'cause they need me*" (10) which suggests that working towards this sense of purpose may increase motivation for recovery.

3.7.3.3. Code 3: Developing routine and forward planning

This was defined as planning against unpredictability and creating an adapted world. This was supported by only 3 studies (ID:9,12,18) but highlighted the benefits of developing a routine and using foresight to prevent failure. A participant from study 12 stated "*When shopping, I always prepare a shopping list, I write my list based on the route I take through the shop*" (12) ensuring the individual has enough energy to complete the task successfully and shows the ability to adapt. However, a lack of spontaneity may have negatives as stated within study 9 discussing the strain on relationships *"After some time, the changes in marital life became routine*".

3.7.3.4. Code 4: Progression is gradual process

This was defined as the awareness of progress and being either a positive recipient or frustrated. This was supported by 7 studies (ID:1,2,3,7,8,11,17) suggesting that its key to live each day by day and to record progress. Study 1 explained "*As she was able to resume doll making, in her mind, she was not stuck at one place in time but rather was working along an ever-changing continuum toward a better QOL*". This encompasses the theory of being a passive recipient, however some participants within study 8 found slow progress as being negative and not recognising their recovery, preventing them from progressing.

Detail of model development.

1) Following the initial synthesis of data, Mind mapping was undertaken by highlighting key words from the data.

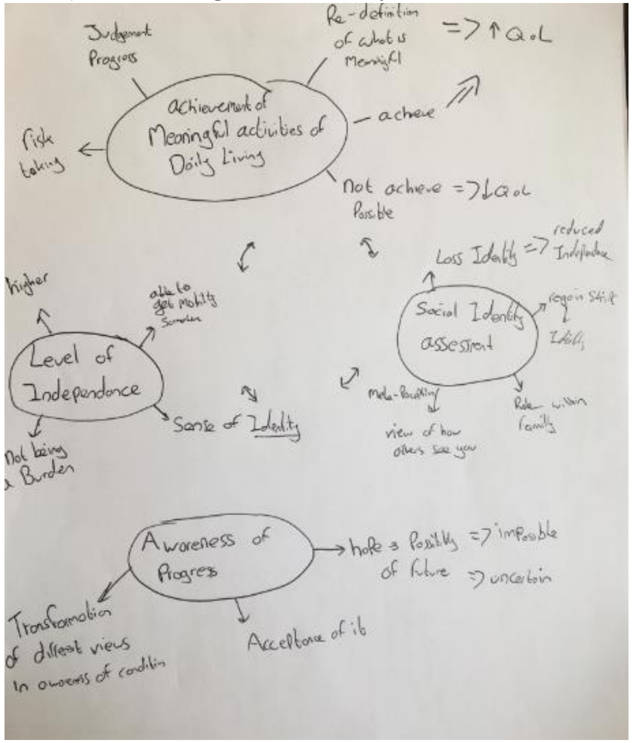

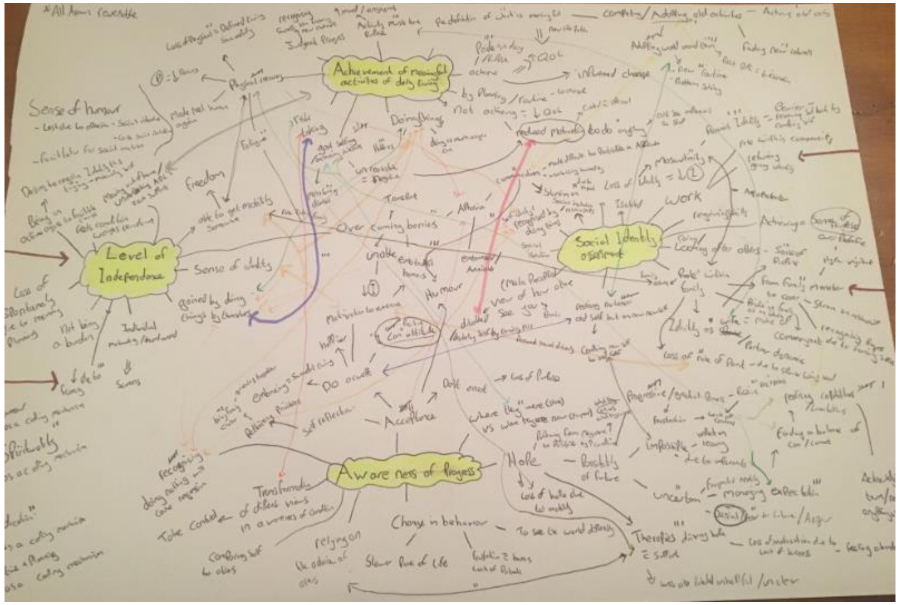

2) The results sections of all 18 studies were then synthesis and observations from the literature were recorded, expanding on the 4 main themes and visibly illustrating the connection between themes (Main themes highlighted in yellow):

3) Sub themes were then highlighted (Blue):

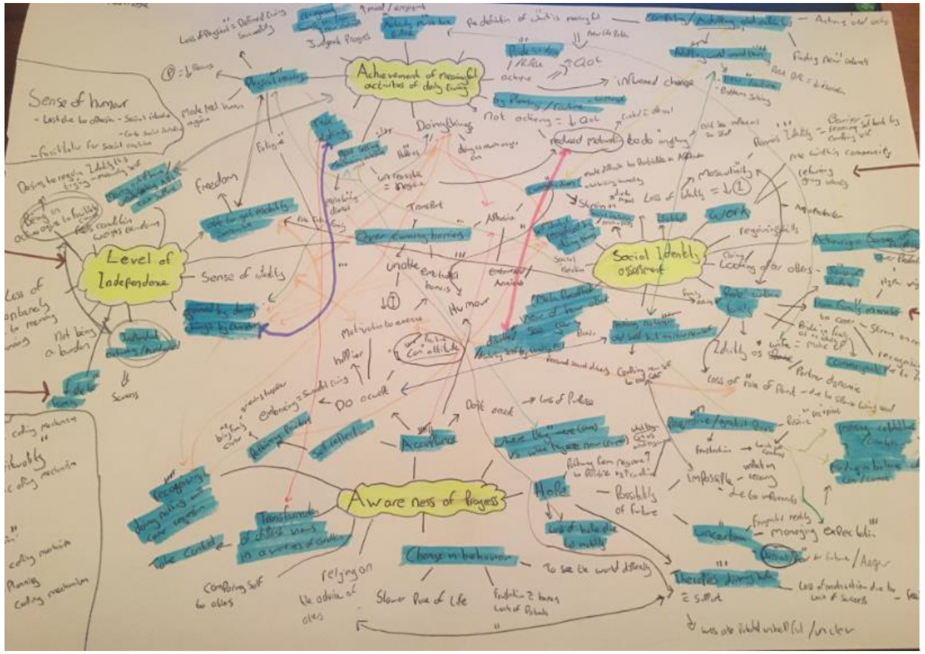

4) The main themes were identified and the sub themes were categories accordingly (Print screen mindmap table)

Mind mapping –

Meaningful Activities of daily living	Social Identity	Awareness of progress	Level of independence
Achieving activities which are purposeful	Identified by what they can achieve	Acceptance	Gained independence by doing thing by themselves
Physical recovery defined successful living	Achieving a sense of purpose	Recognising doing nothing will cause regression	Ability to mobilise somewhere (travel of functionally)
Adapting the world around them	Role within the family (negative and positive)	Progression is gradual process (positive recipient/ frustration)	Overcoming barriers
Completing/ adapting old activities	Communication	Where they were compared to where they are now	motivated/ hardworking/ positive "I Can" attitude facilitating recovery
Risk taking	Meta-perception. Ways in which other see them (disabled/ disabling self by thinking this)	Denial and uncertainty	Family/ support systems reducing independence
Goal setting/ Achieving milestones (good and if unrealistic =bad)	Work identity	Realising limitations and capabilities, finding a balance of can and cannot	Unsupported achievement of activities of daily living
Pride in doing/ purpose	Recognising no longer old self but instead a new identity	Loss of hope due to inability	
Recognising success then tackling new challenges	Barrier due to comparing old self to new self	Change in behaviour – to see the world differently	
Developing routine and forward planning	Meaningful social relationships	Due to what is achieved with Therapists	
	Isolation/ social inclusion	Self-reflection – rethinking priorities	
		Awareness of condition – taking control	

5) Following this a re-organisation of the themes was needed to display more accurately what was being produced within the literature

Final Reorganization of Mind mapping –

Barriers and facilitators	Risk taking	Achievement of meaningful activities or interactions	Social Identity (past, present, future)
Social<br>• Family/ support systems<br>• Meaningful social relationships<br>• What is achieved with Therapists<br>• Isolation and inclusion	Recognising doing nothing will cause regression	Achieving purposeful activities	Re-evaluation of social identity<br>• Recognising progress and goal setting<br>• Self-reflection and rethinking priorities
Physical<br>• Physical recovery defining hope<br>• Mobilisation status	Adapting the world and changing behaviour	Recognising success then tackling new challenges	Present social identity and adapted self<br>• Identified by what they can achieve<br>• Role within the family/ Work identity
Internal<br>• Acceptance<br>• Motivated and attitude<br>• Hope in possibility	Hope as a paradox, realise limitations and capabilities	Independence achieved by doing	**Future and possible social identity**<br>• Completing/ adapting old activities<br>• Achieving a sense of purpose and Pride<br>• Developing routine and forward planning<br>• Progression is a gradual process

6) Following this, a discussion with the second author (A.S) for focus on which key areas to display within the model took place. This led to the model drafting process seen below:

1st draft of Model

- This draft was missing the like that barriers and facilitator may both influence two separate sections of the model

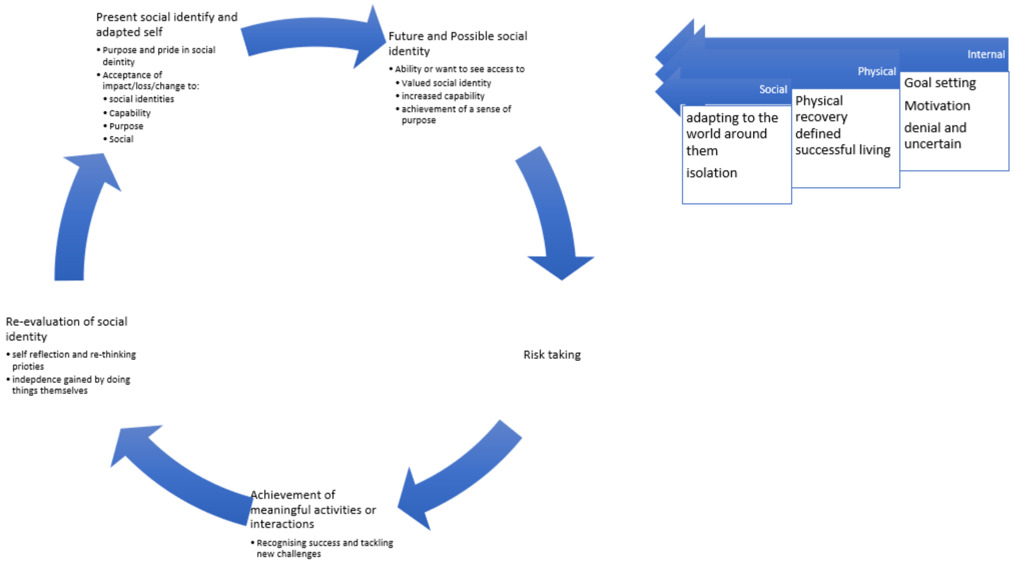

2nd draft of Model

- This draft now displays that barriers and facilitators may impact the cycle at separate points, however this needed to be more easily distinguishable
- More information was added also on each theme

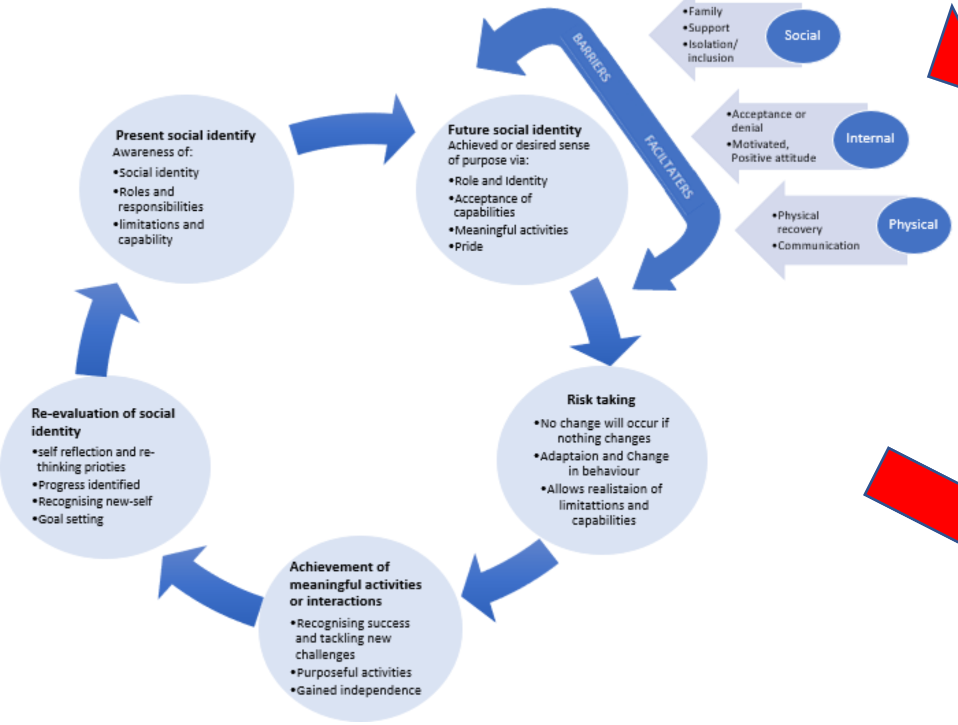

3rd draft of Model

1. This final draft now represents where barriers prevent the cycle going forward and how facilitators are directly linked with risk taking and continuing the cycle.

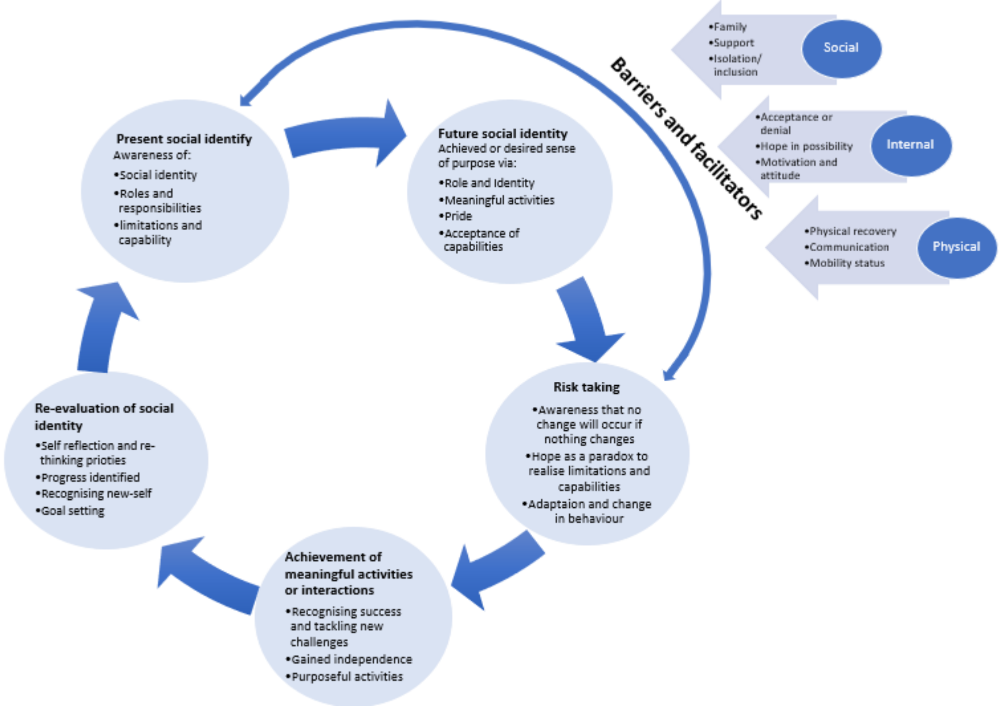

Supplement: Supplementary file 1 [file behavsci-10-00046-s001.pdf]
